# Acoustic Detection of Intracranial Cavitation Induced by Blunt Impacts in Polyacrylamide Human Head Models Across Varying Orientations

**DOI:** 10.1007/s10439-025-03895-9

**Published:** 2025-11-23

**Authors:** Eric J. Galindo, Michaelann S. Tartis

**Affiliations:** Department of Chemical Engineering, New Mexico Institute of Mining and Technology, Socorro, NM 87801 USA

**Keywords:** Traumatic brain injury, Plane wave imaging, Passive cavitation, Human head models, Polyacrylamide, Brain phantoms

## Abstract

**Purpose:**

Traumatic brain injury remains a major health concern among civilians and military personnel, with intracranial cavitation hypothesized as a damage mechanism during blunt impacts.

**Methods:**

This study examines cavitation bubble activity in simplified polyacrylamide human head models, focusing on different anatomical regions and imaging modalities. A drop tower setup with high-speed acoustic and optical imaging was used to characterize the onset, expansion, and collapse of bubbles and assess the impact orientation’s effects.

**Results:**

Acoustic plane wave imaging and passive cavitation detection captured emissions linked to bubble dynamics. Although plane wave imaging was affected by reflections, it detected bubble growth effectively. In contrast, passive cavitation detection showed greater sensitivity during collapse, with broadband spectral responses. Signal processing extracted relevant spectral features from both modalities, regardless of pre-existing bubble nuclei. Cavitation behavior varied across models, with impact angle influencing both timing and persistence, suggesting orientation affects injury mechanisms. When the head model was impacted at a 90° angle and observed along the central sulcus, cavitation onset occurred earliest with the strongest shockwave reflections, likely due to changes in wave travel distance between the coup and contrecoup sites. Head models with artificial dampeners showed that the scalp and dura mater layers reduced cavitation intensity, though cavitation remained detectable.

**Conclusion:**

This work supports the feasibility of acoustically detecting impact-induced cavitation as a standalone tool, informing strategies for transcranial monitoring and protective gear design in blunt trauma scenarios.

**Supplementary Information:**

The online version contains supplementary material available at 10.1007/s10439-025-03895-9.

## Introduction

Traumatic brain injuries (TBIs) are a leading cause of disability and death, affecting millions of civilians and military personnel of all ages worldwide [1–5]. In civilian populations, most reported TBIs result from blunt impacts commonly associated with everyday activities. Military personnel, however, are exposed to a broader range of blunt and blast impacts, often stemming from explosive devices encountered during training or combat scenarios [6–8]. In the United States, the majority of statistical data on military-related TBIs were collected during the Iraq–Afghanistan war, during which over one hundred thousand TBIs were reported [7–9]. Consequently, rehabilitation has remained a prolonged and ongoing process for many individuals, extending long after their medical discharge. Presently, awareness of TBIs has grown substantially in both civilian and military communities, primarily driven by advances in scientific research and data reporting. Although military-grade protective equipment continues to improve annually, the increased use of explosives in both battlefield operations and training environments since the Iraq–Afghanistan conflict has raised ongoing concerns about the long-term solutions for TBIs [10, 11]. Additionally, despite technological and clinical advancements, neurological research still lacks a comprehensive understanding of the underlying damage mechanisms and their roles in psycho, patho, and physiological disorders. This persistent knowledge gap limits the effectiveness of protective equipment development and hinders progress in the reliability and efficacy of clinical-based recovery programs [4, 5].

One of the most significant challenges in TBI research is the inability to directly monitor in vivo tissue deformation and pressure changes during impact in living systems [12, 13]. Overcoming this limitation could yield critical insights into potential injury mechanisms. Intracranial cavitation is widely suspected to contribute to damage, particularly in the tissue–fluid interfaces such as the sulcal, perivascular, and periventricular sites [14–17]. Cavitation is believed to occur at the contrecoup region of the human head during blunt or blast impacts, following the Friedlander waveform model [12, 16, 18, 19]. Impact-induced cavitation is generated through overpressure within the human head, thus accelerating cerebrospinal fluid (CSF) and reducing static pressure below the vapor pressure (typically below − 100 kPa at STP), thereby inducing cavitation under the pressure differential [19–21]. As bubble nuclei expand under vacuum, they collapse violently as static pressure rises, generating shockwaves and jetting phenomena [15, 17]. The longitudinal nature of these shockwaves induces shear deformation in brain tissue, resulting in localized shear strains as the waves propagate through the CSF into periventricular, perivascular, and cortical surfaces, led by high deformation strain rates [17, 19, 21–23]. However, no direct visual evidence confirms that cavitation occurs within the CSF during impact events in the in vivo human brain. Therefore, exploring and optimizing techniques to better mimic in vivo characterization is favorable; hence, acoustic imaging is a promising approach for detecting cavitation at different depths where other techniques are limited.

Acoustic and high-speed optical imaging are powerful techniques for observing cavitation bubble dynamics in soft matter materials and low-viscosity fluids [24–28]. Shadowgraph imaging visualizes cavitation and shock waves through changes in the refractive index; however, it is limited to transparent mediums and provides 2D planar images without depth information inside a 3D system [29–31]. In contrast, acoustic diagnostic imaging techniques are well established for detecting various forms of cavitation, such as stable and inertial types, through signal processing of harmonic or broadband signatures resulting from nonlinear oscillations and wave propagation at different depths [32, 33]. A high-speed variant of acoustic imaging, plane wave imaging (PWI), can insonify and detect transient events at several thousand frames per second (FPS) using a transmit plane wave generated through synchronous triggering of all piezoelectric transducer elements, from which echoes are captured [34, 35]. Another technique, passive cavitation detection (PCD), does not require a transmitted wave but instead receives acoustic emissions generated by cavitating bubbles. PCD is often used alongside passive cavitation mapping, in which cavitation sources’ spatial locations and energy are estimated, as demonstrated in medical diagnostic applications. One example is histotripsy, where bubble clouds are formed through focused ultrasound beams for therapeutic purposes [27, 33, 36]. Ultimately, PWI and PCD techniques can also be helpful when detecting intracranial cavitation in various blunt TBI scenarios in simplified human head models.

The Anthropomorphic Neurologic Gyrencephalic Unified Standard (ANGUS) surrogate is a human head model developed for experimental and in silico studies of blunt- and blast impact-induced cavitation [16, 19, 31, 37, 38]. Its geometry is derived from human anatomical components, and its material properties have been characterized through viscoelastic testing, making it a reasonable model for studying cavitation bubble dynamics [39, 40]. Its controlled design makes it well suited for high-speed optical and acoustic imaging studies of these phenomena. Linear kinematics of the head during impact have also been explored to inform chronic traumatic encephalopathy (CTE)–related research in military and sports contexts [16, 19]. This work builds upon this established model to extend application to orientation-dependent cavitation and impact studies.

In this study, PWI and PCD are employed to detect blunt-induced cavitation in simplified human head models subjected to blunt impacts. The focus is on tissue–fluid interfaces, such as the central sulcus, cortical sulci, and ventricles, where cavitation is thought to occur. The primary objective is to evaluate and compare the effectiveness of these techniques in detecting cavitation at depth, utilizing various acoustic imaging locations and impact orientations. Signal processing methods are applied to extract spectral features associated with cavitation. Additionally, PCD is investigated as a feasible approach for cavitation detection in a transcranial application through established cavitation thresholds. Ultimately, the outcome of this work advances the understanding of the role of cavitation in blunt trauma and informs the development of improved protective headgear designed to minimize pressure differentials and mitigate brain injury.

## Methods and Materials

### Fabrication of the ANGUS Phantom for Mimicking Simplified Anatomical and Shear Properties of Human Brain Tissue

**Fig. 1:**
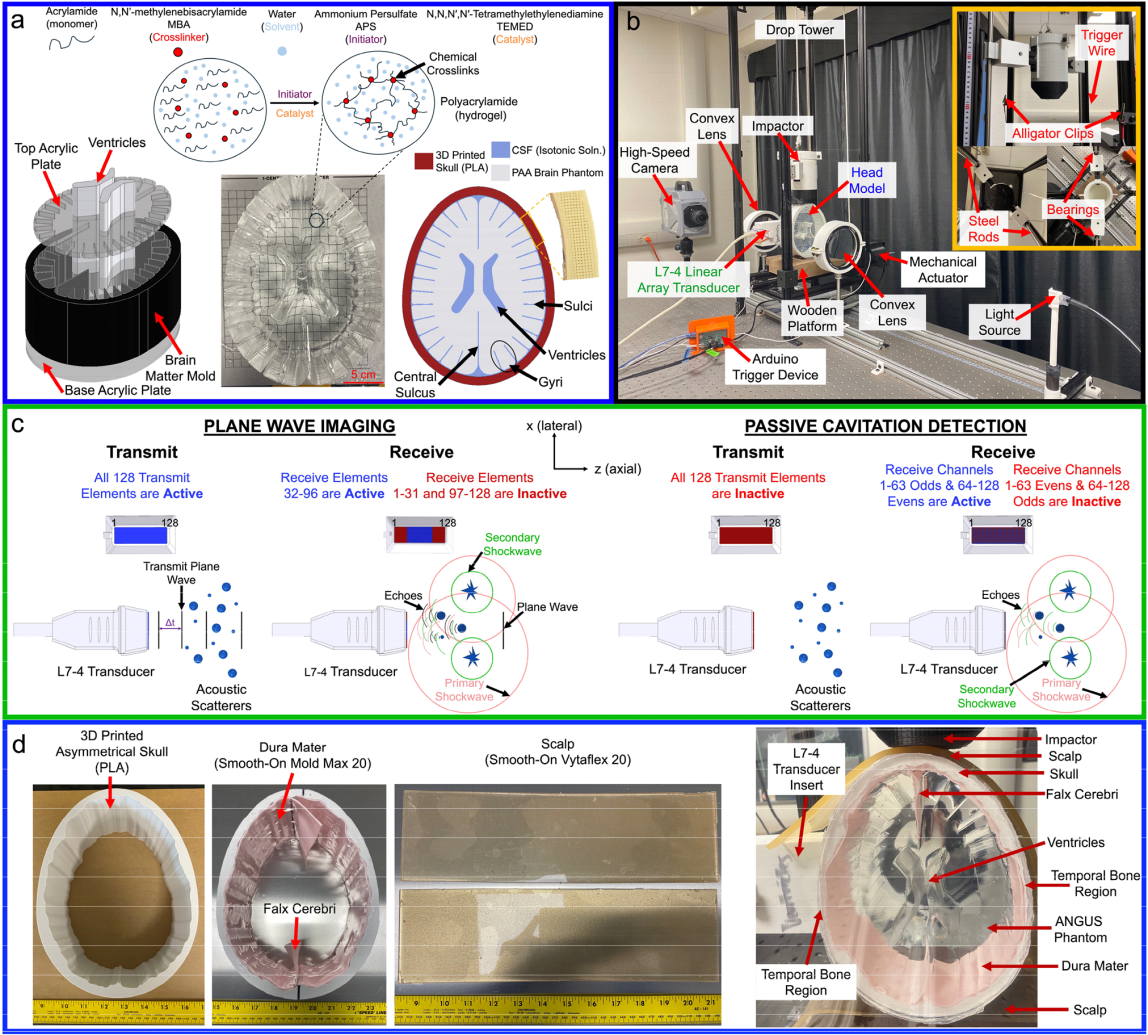
A schematic representation of the ANGUS brain phantom fabrication process **a**, which is made of polyacrylamide (PAA) and poured into 3D-printed molds to create simplified anatomical features. The shadowgraph imaging setup **b** has two convex lenses, a high-speed camera, and a light source. The drop tower and head model are positioned within the parallel light field to visualize cavitation bubble dynamics during impact. The drop tower incorporates an impactor, which is filled and raised to a specified mass and drop height, and a wooden platform. The impactor then linearly descends, breaking the trigger wire and initiating synchronous data acquisition between optical and acoustic imaging systems. Acoustic plane wave imaging **c** transmits a planar wave to an area of interest at a specific acquisition frame rate (Δt), where reflected echoes from bubble sources and shockwaves from bubble collapses are received with the central 64 elements. In contrast, passive cavitation detection does not induce a planar transmit wave, eliminating any image reconstruction and spectral effects caused by the plane wave. Instead, it receives rich information from alternating 64 channels, detecting cavitation bubble sources echoed from shockwave activity. Artificial geometries and dampeners **d** were integrated into the human head model through an asymmetrical 3D-printed skull, a dura mater layer with falx cerebri, and a scalp mimic. The linear array transducer detects cavitation through two scenarios: either a cutout region along the exterior of the skull or by integrating a temporal bone region for an acoustic transcranial approach

The fabrication process of the ANGUS phantom is similar to those previously reported for mimicking the shear mechanical properties of human brain tissue using polyacrylamide (PAA) while monitoring swelling behaviors to replicate ventricular, gyrus, and sulcal features [16, 31, 37–40]. The ANGUS phantom, Fig. 1a, is comprised of a 10% (w/v) 60-1 (monomer-to-crosslinker) PAA formulation to achieve the visual transparency needed for high-speed optical imaging. To fabricate PAA, a homogeneous mixture between degassed deionized (DI) water, monomer (acrylamide (purity ≥ 98% (gas chromatography))), and crosslinker (N’-Methlenbis(acrylamide)(MBA, purity 99%)) is first achieved. To initiate crosslinking between acrylamide chains, an initiator (ammonium persulfate (ACS Reagent, ≥ 98%)) is implemented and stirred alongside a catalyst (N,N,N’,N’-tetramethylethylendiamine (TEMED, ReagentPlus®, 99%)) to increase the reaction rate and polymerize the hydrogel exothermically. Once the catalyst is integrated, the PAA solution is poured into a 3D-printed brain mold and ventricles alongside acrylic plates to construct simplified anatomical features and smooth surfaces. Lastly, the phantom is left to polymerize at room temperature (20–23 °C), left to swell for 24 h in isotonic solution (ISOTON® II Diluent, Beckman Coulter), and sealed inside a 3D-printed human skull model, made out of polylactic acid (PLA), in preparation for blunt impact testing. All chemicals were purchased from Sigma Aldrich, USA.

### Modification of the ANGUS Head Model through the Integration of Asymmetrical Features and Artificial Dampeners

The integration of asymmetrical features into the 3D-printed cranium (Fig. 1d), with varying skull thicknesses (3.5–10 mm), is inspired by a trans-axial planar computed tomography (CT) scan of the human head [37, 41]. The scalp mimic is fabricated with a castable flexible urethane rubber, Smooth-On Vytaflex™ 20, which has similar mechanical properties to human scalp, where other researchers have previously incorporated it into head models intended for blunt TBI scenarios [42]. To construct the polymeric scalp, a homogeneous mixture of prepolymer and curing agent (1:1 v/v) is prepared. The solution is poured into rectangular acrylic molds (335 × 116.4 × ~ 3.5 mm) and left to cure for 24 h at room temperature (20–23 °C). Each scalp layer is placed between the impact contact points of the head model, such as the impactor and wooden base block. The dura mater and falx cerebri are fabricated with Smooth-On MoldMax™ 20, a tin-cured silicone rubber. To fabricate the dura mater, a homogeneous blend of base polymer and curing agent (100:10 w/w) is produced, with the solution periodically degassed for 15 min in a vacuum chamber (− 0.9 bar) to remove air bubbles. The silicone rubber is poured into rectangular molds (335 × 116.4 × ~ 1 mm) and left to cure for 24 h at room temperature (20–23 °C). Finally, the dura mater is manually adhered to the inner wall of the skull geometry with Smooth-On SilPoxy™, leaving a 40 mm overhang in the vertical midplane to simulate the falx cerebri.

### Assembly and Impact Orientations of ANGUS Head Models for Blunt Impact Testing

**Fig. 2:**
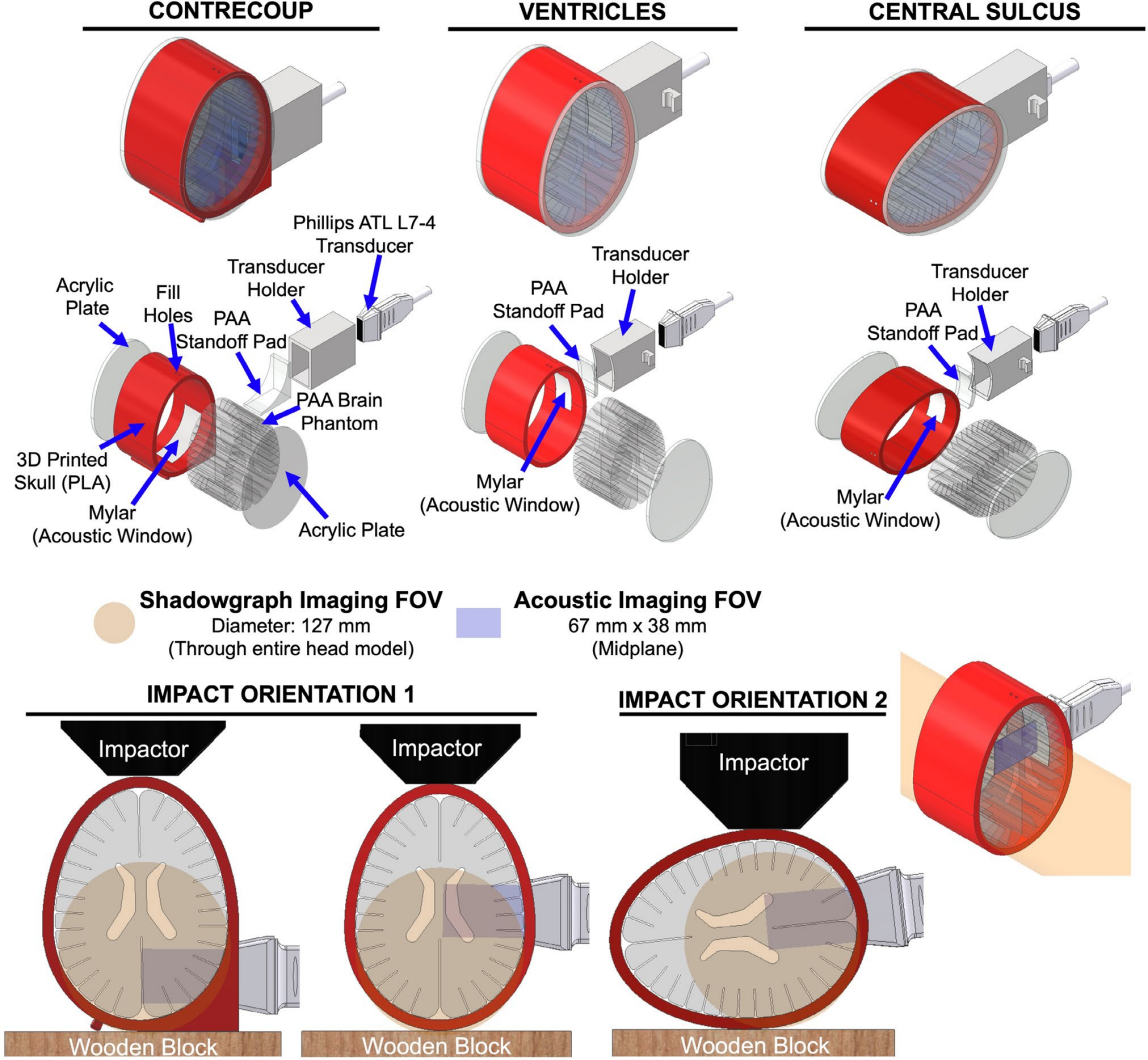
ANGUS head models were fabricated and assembled for various impact and imaging orientations. Each head model comprises an ANGUS brain phantom sealed in a 3D-printed skull with a window for acoustic observation, isotonic solution, and acrylic plates. The acoustic windows are filled with PAA to increase acoustic image quality and limit bubbles in the coupling gel. The first head model positions the transducer in the contrecoup, observing up to the central sulcus and adjacent gyral and sulcal regions. The second head model positions the transducer with the primary observation of the ventricles and cortical sulcal regions. The third head model is rotated 90° compared to the first and second head models, where the acoustic transducer observes along the axis of the central sulcus. The head models for observation in the contrecoup and ventricles are impacted from an upright position, whereas the third rotated head model is impacted at the apex of curvature. The acoustic transducer is positioned in the midplane of every head model imaging at depth (blue), where shadowgraph imaging visualizes cavitation events throughout the entire head model (orange)

The assembly of the ANGUS human head model (Fig. 2) follows a similar protocol to those previously reported for blunt impact testing [31, 37]. To begin, a skull geometry depicting an extruded trans-axial planar slice of the human head is 3D printed with PLA with a uniform thickness of approximately 8 mm. For acoustic imaging purposes, each skull geometry has a rectangular cutout (70 × 38 mm) through the midplane of a sidewall (at depth), acting as a window for acoustic data acquisition. The inner sidewall of the acoustic window is covered with a six-mil transparent mylar sheet and adhered with a thermoplastic adhesive to prevent leakage. Two 1/4”-thick acrylic plates are attached to the front and rear of the skull model to fully enclose the swollen ANGUS brain phantom with isotonic solution to mimic CSF, as shown in Fig. 1a [20]. The model is then sealed, and any air bubbles are removed through fill holes, which are covered with rubberized tape. When cavitation events are visualized only in the bulk fluid, without a brain phantom, DI water is used as a substitute. A 10% (w/v) 60-1 PAA solution is added to the acoustic window to reduce the need for coupling gel prone to large bubbles that obstruct imaging, which can introduce unwanted scattering in acoustic images. A 3D-printed transducer holder is mounted on the window to keep the transducer in place during impact, reinforced with foam and a strap.

Each head model is designed for distinct optical and acoustic observation of tissue-fluid interfaces during blunt impact. The first model places a linear array transducer at the contrecoup, enabling acoustic imaging through the PAA phantom and up to the central sulcus, including adjacent gyral and sulcal structures. The second model is optimized for observing events up to the ventricles and surrounding cortical structures. The third model is rotated 90° relative to the first two, positioning the transducer to image along the depth axis of the central sulcus. Shadowgraph imaging captures all cavitation events throughout the depth of the head model during impact, while acoustic imaging receives events in the midplane. Although both techniques detect bubble activity, the frames are collapsed into two-dimensional frames: shadowgraph imaging lacks depth resolution through the head model, and ultrasound does offer depth information but lacks lateral height and volume. Two primary impact orientations are employed. The first involves impacting the head models, for contrecoup and ventricular observations, in an upright position. The second orientation is implemented for the rotated model, designed to observe the central sulcus, with impact directed at the apex of curvature of the 3D-printed cranium.

### Drop Tower Assembly and Shadowgraph Imaging Setup for Intracranial Cavitation Production and Observation

A drop tower and shadowgraph imaging setup (Fig. 1b) were previously designed and characterized to study cavitation thresholds and visualize bubble dynamics in ANGUS human head models under blunt dynamic loading [31, 37]. The primary components of the drop tower include aluminum framing, a 3D-printed hollow planar impactor constructed from polylactic acid (PLA), linear rods for uniform descent, and a wooden base platform. To begin, the impactor is filled with a user-defined mass and raised to a specific drop height, held in place by an anchor hook and a mechanical actuator. An electronic button integrated into an Arduino UNO R3 is manually pressed, retracting the actuator and releasing the impactor. As the impactor descends linearly, the planar bottom edge disrupts a trigger wire, synchronously triggering optical and acoustic data acquisition hardware, and lastly impacting the head model placed on the wooden platform. The shadowgraph imaging setup involves two Wollensak MTD telescope convex lenses (diameter: 127 mm; focal length: 700 mm), a Photron SAZ high-speed camera, a Nikon AF-S DX Zoom Nikkor 18–55 mm zoom lens recording at 100,000 FPS with a shutter speed of 0.16 µsec, and a Sugar CUBE LED (Ultra White) illuminating the parallel light field. The drop tower and head model are positioned between both convex lenses where each head model was impacted with a mass of 4 kg and a drop height of 60 cm, known to induce reliable cavitation on a test-to-test basis [31, 37]. Cavitation bubble monitoring was performed qualitatively on groups of three to six bubbles, tracking them frame by frame to observe the timing of the initiation of bubble growth, maximum bubble size, and collapse events that generated shockwaves.

### High Frame Rate Plane Wave Imaging and Passive Cavitation Detection During Blunt Impact

A Verasonics Vantage 64 (Verasonics®, Inc.) ultrasound research scanner enables PWI and PCD with similar hardware and imaging parameters as previously reported [31, 37]. To perform acoustic data acquisition, a Philips ATL L7-4 linear array transducer is integrated with a center frequency of 5.20 MHz, an acquisition frame rate of 8620 FPS, and a transmit voltage of + 17.0 V. This setup establishes an axial and lateral field of view (FOV) of 67 and 38 mm, respectively. Figure 1c displays a schematic of the data acquisition sequence for both PWI and PCD imaging modalities, where each acquisition consists of a transmit and receive sequence. For PWI, the transmit sequence briefly transmits a planar wave to the region of interest, generated from all 128 transducer elements. Consequently, the receive sequence is limited to 64 user-defined elements, with the central elements (32–95) acquiring raw information from incoming echoes and shockwaves from cavitation bubble collapse. The passive cavitation imaging sequence is similar to that of PWI; however, the transmit is turned off, and the active 64 receive channels alternate along the transducer’s elemental array. Specifically, every odd channel from 1 to 63 and every even channel from 64 to 128 is selected. Translation from intensity to pixel data is processed through the Verasonics built-in image processing scripts, which remove contrast enhancers and minimize filtering. The raw radiofrequency (RF) data are translated and analyzed in the frequency domain using MATLAB’s Signal Processing Toolbox.

### Baseline Subtraction and Normalization of Acoustic Datasets for Scatter Reduction and Determination of Energy Differentials

**Fig. 3:**
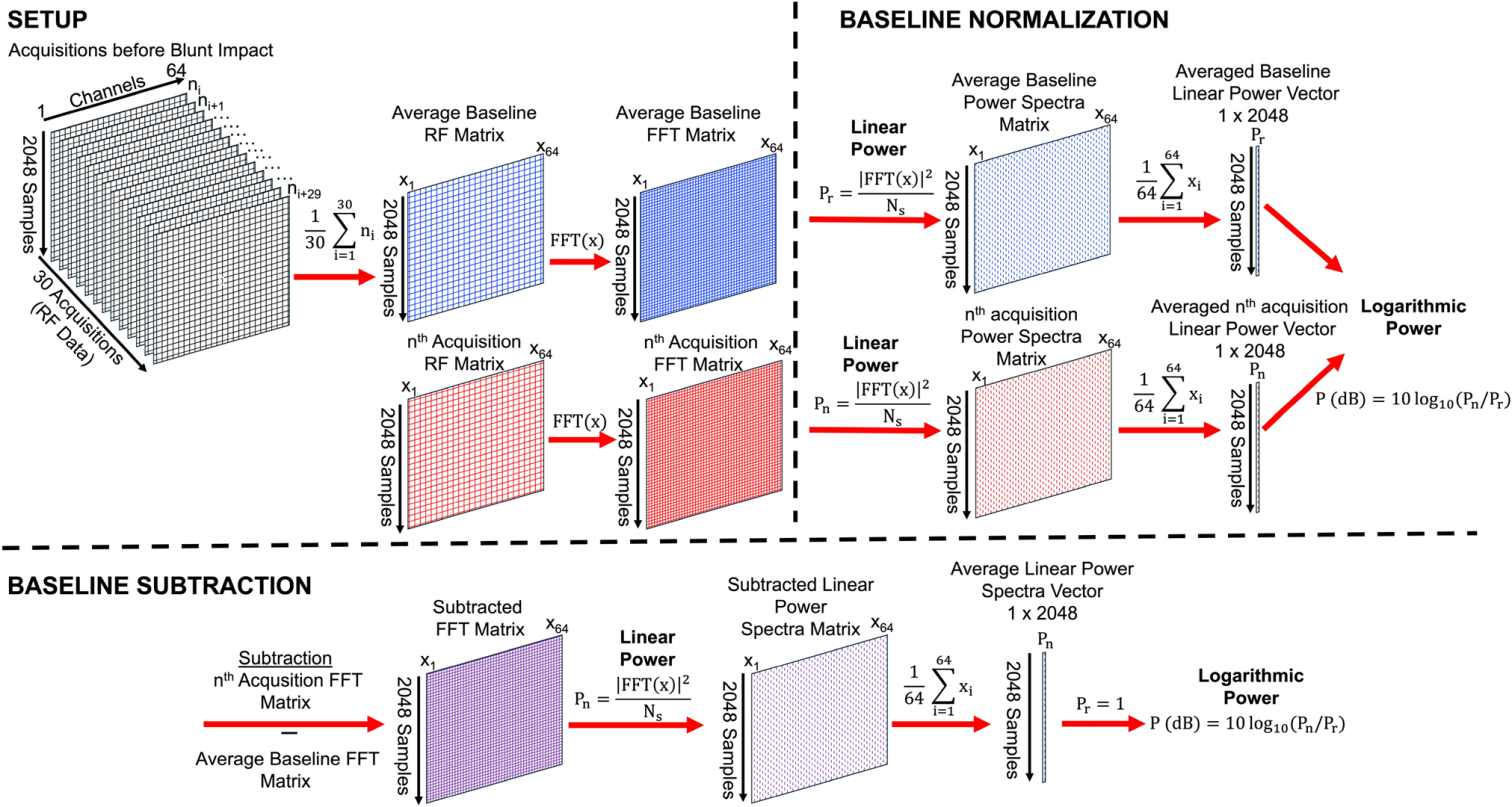
Schematic representation of baseline subtraction and normalization operations to minimize pre-existing scatterers and observe energy differentials caused by cavitation bubble dynamics during blunt impact. These methods are applied for plane wave imaging and passive cavitation detection to determine which operations best serve each technique.

The baseline subtraction and normalization methods (Fig. 3) follow a similar methodology to minimize and discern pre-existing scatterers and observe potential pressure differentials caused by cavitation bubble dynamics [37]. These techniques are applied to PWI and PCD datasets to understand which operations best serve each method. Before these operations, an average baseline and an acquisition of interest must be created and selected. To compute the averaged baseline, 30 frames of RF data from all 64 receive channels are chosen before blunt impact, which is then averaged into a single matrix of size 2048 × 64 (samples × channel number). Next, a user-defined acquisition of interest (*n*^th^) is selected, and both the *n*^th^ and averaged baseline matrices are translated into the frequency domain using MATLAB’s built-in discrete Fourier transform (FFT) function along each column vector for all 64 receive channels.

For baseline normalization, the frequency-dependent average baseline and *n*^th^ matrices are converted to a linear power spectra, where the data are averaged across all channels to create a single-column vector. To convert to a logarithmic power spectra, the acquisition (*P*_*n*_) is divided by the averaged baseline (*P*_*r*_) to extract the energy differences between cavitation and areas where no cavitation is known to occur before impact. The baseline subtraction method subtracts the frequency-dependent average baseline from the *n*^th^ matrix, which is then translated into a linear power spectra (*P*_*n*_). The matrix is then averaged across all channels to construct a single-column vector, which is then referenced to 1 dB (*P*_*r*_) to express the spectral behavior of cavitation events during blunt impact with the exclusion of pre-existing nuclei that grow in the head model. The exact process can be applied to a single channel of interest, from which a column vector can be extracted from the averaged baseline and *n*^th^ matrix.

## Results

### Impact Orientation Affects Bubble Onset Time, Location, and Persistence

**Fig. 4:**
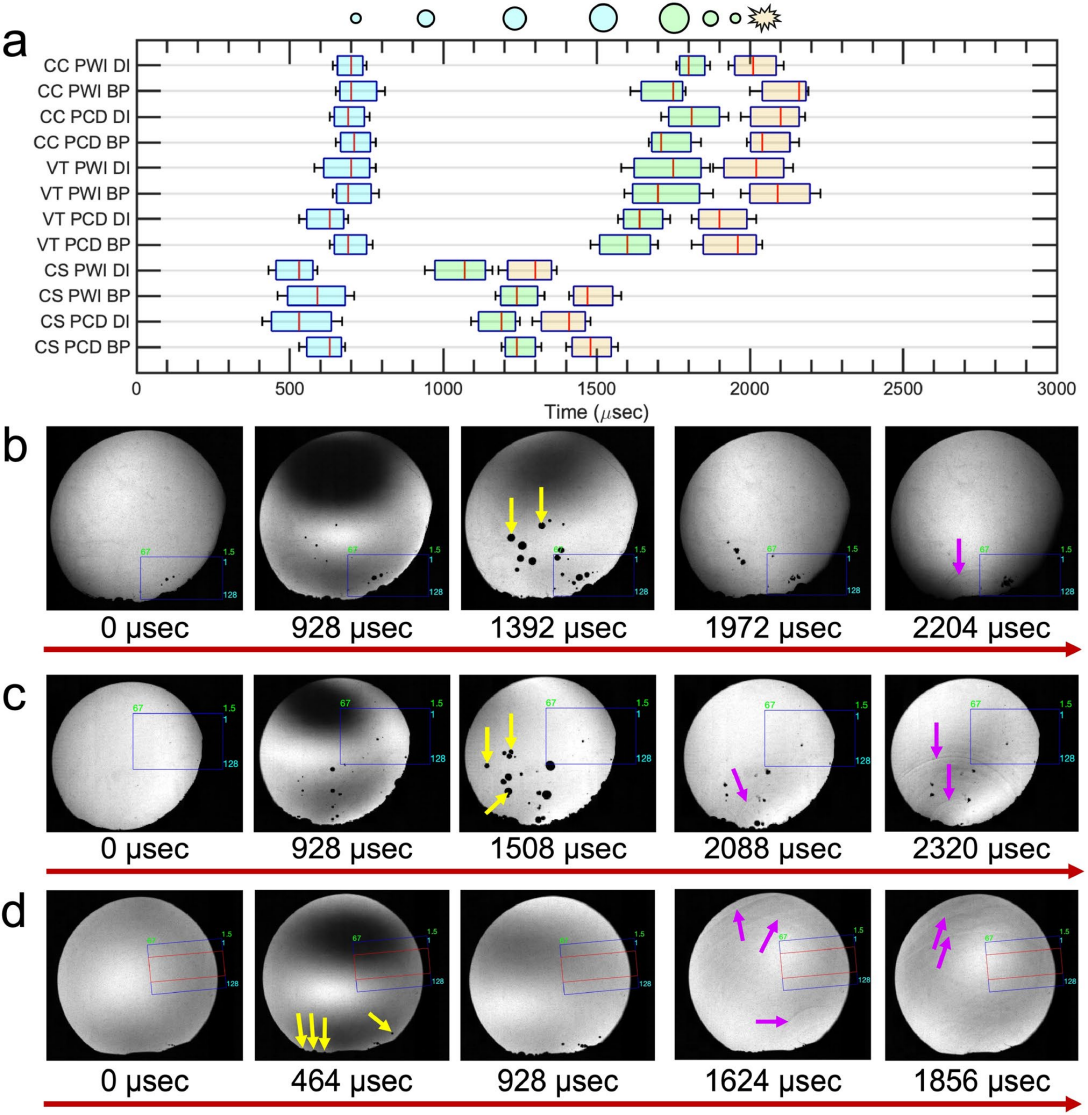
Comparison of cavitation bubble activity across all head models (*N* = 3) by monitoring **a** the onset of cavitation bubble growth (light blue box plot), maximum bubble size (light green box plot), and bubble collapse (light-orange box plot) in deionized (DI) water and ANGUS brain phantom (BP) mediums intended for plane wave imaging (PWI) and passive cavitation detection (PCD). Timelines of a representative test for head models for observation in **b** contrecoup (CC) and **c** ventricles (VT) in a PCD imaging setup filled with DI water. Observation of the **d** down the axis of the central sulcus (CS) is imaged in a PWI setup and filled with DI water. Yellow arrows indicate regions of cavitation bubbles, while magenta arrows highlight shockwave activity. The blue rectangular box overlaid onto the shadowgraph frames represents the projection of the 128 transmit channels, with an imaging depth of 1.5–67 mm, which is also used for the PCD receive sequence. The red box indicates the receive channels for PWI (channels 32–96), achieving the same imaging depth as the transmit sequence

All head models filled with DI water and ANGUS brain phantoms were evaluated to identify variances in cavitation bubble dynamics across different impact orientations and transducer locations. Figure 4a shows the time point when cavitation bubble nuclei, driven by the pressure differential upon blunt impact (0 µsec), begin to grow, as indicated by the light blue box plot. It also shows the onset of bubble collapse after the bubbles reach their maximum critical size, represented by the light green box plot. Finally, the yellow box plot reveals the complete collapse of the cavitation bubbles with shockwave generation. All box plots were validated through qualitative observations of shadowgraph frames, using a sample size of three head models that were impacted similarly. Head models associated with observations in the contrecoup and ventricles show similar timing for bubble growth in both DI water and brain phantom mediums, with growth occurring at 673 ± 74 and 709 ± 63 µsec, respectively. Their onset of bubble collapse times is also comparable, occurring at 1753 ± 114 and 1693 ± 113 µsec for DI water and brain phantom mediums, respectively. Notably, the model involving the ventricular window shows the start of bubble collapse about 149 ± 109 µsec faster than observing the contrecoup. When observing along the depth of the central sulcus, bubble behavior is consistent across all mediums. However, bubble growth begins between 563 ± 99 µsec, roughly 117 ± 97 µsec sooner than in the contrecoup and ventricles models. The onset of bubble collapse also occurs between 1183 ± 110 µsec, about 588 ± 147 and 493 ± 180 µsec earlier than in the contrecoup and ventricles, respectively, across all mediums. Complete bubble collapse and shockwave creation were prolonged with ANGUS brain phantoms compared to DI water alone. Specifically, bubble collapse from maximum critical size occurred approximately 15 ± 8, 88 ± 16, and 125 ± 80 µsec slower in the central sulcus, ventricles, and contrecoup regions, respectively, when compared to just DI water.

Visual inspection of the shadowgraph frames reveals distinct differences in bubble activity and shockwave behavior across all head models and mediums. Fig. 4b–d shows representative tests for observing the contrecoup, ventricles, and central sulcus using DI water to enhance visibility in both PWI and PCD imaging scenarios. Qualitatively, the contrecoup and ventricles models produce similarly sized cavitation bubbles (yellow arrows), most of which form near the center of each model within the bulk medium. After bubble collapse, shockwaves expand radially and reflect off the inner curved skull surface, traveling from contrecoup to coup, as indicated by the magenta arrows. In contrast, when observing the central sulcus, cavitation bubbles are smaller and collapse more rapidly than those in the other two models. This rapid collapse results in more shockwave reflections over time. Supplementary Videos 1, 2, and 3 further support and validate the observations presented in Fig. 4b–d, respectively. In particular, Supplementary Video 3 reveals the propagation of the transmit plane wave throughout the head model, demonstrating its periodic and coherent nature.

### Pre-existing Scatterers and Cavitation Reflections in Brain Models

**Fig. 5:**
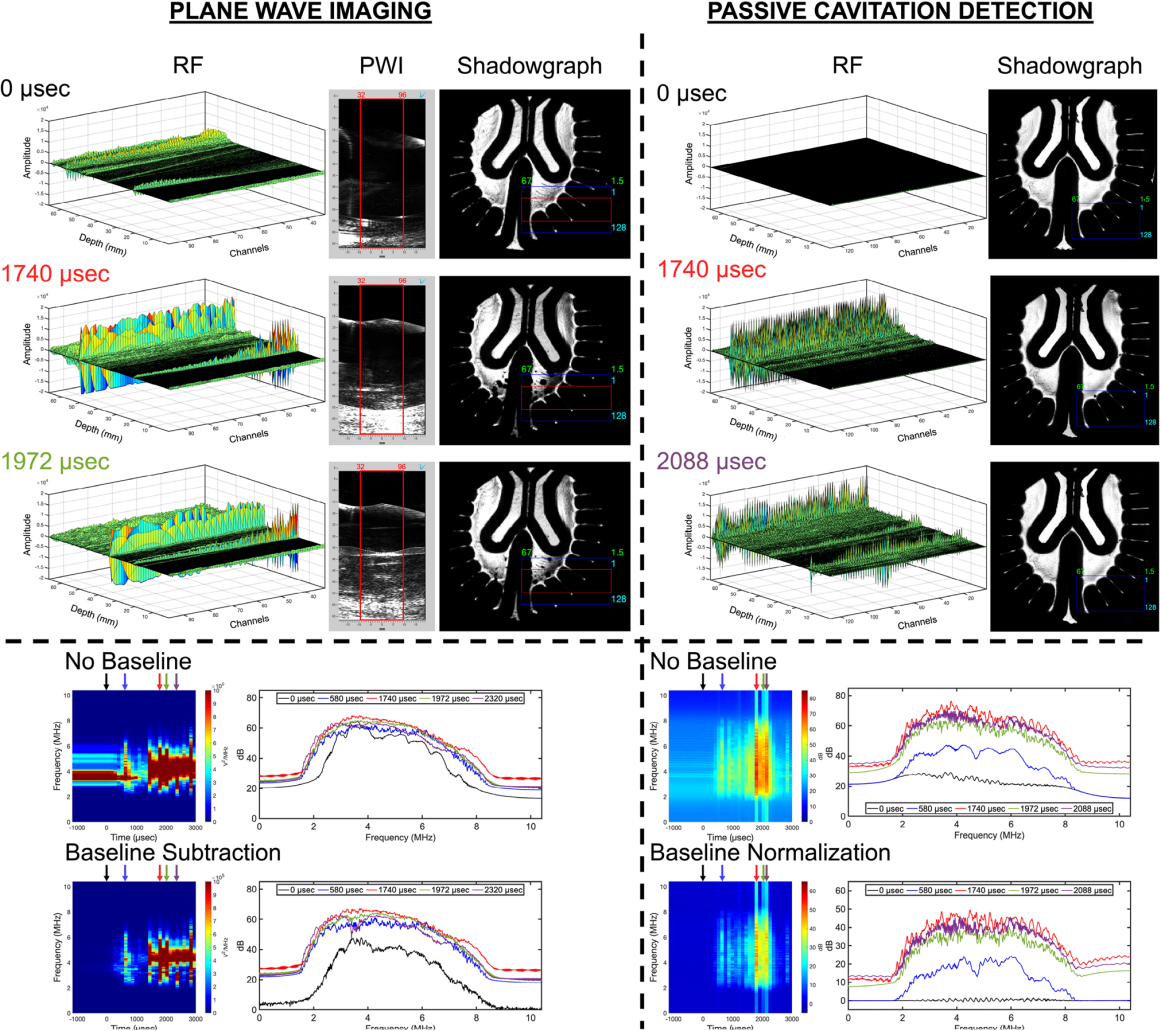
Comparison of plane wave imaging (PWI) and passive cavitation detection (PCD) for observations in the contrecoup. A 3D representation of the radiofrequency (RF) data is presented, along with acoustic and shadowgraph imaging frames. The signal processing pipeline includes raw transformation into the frequency domain, baseline subtraction, and baseline normalization

**Fig. 6:**
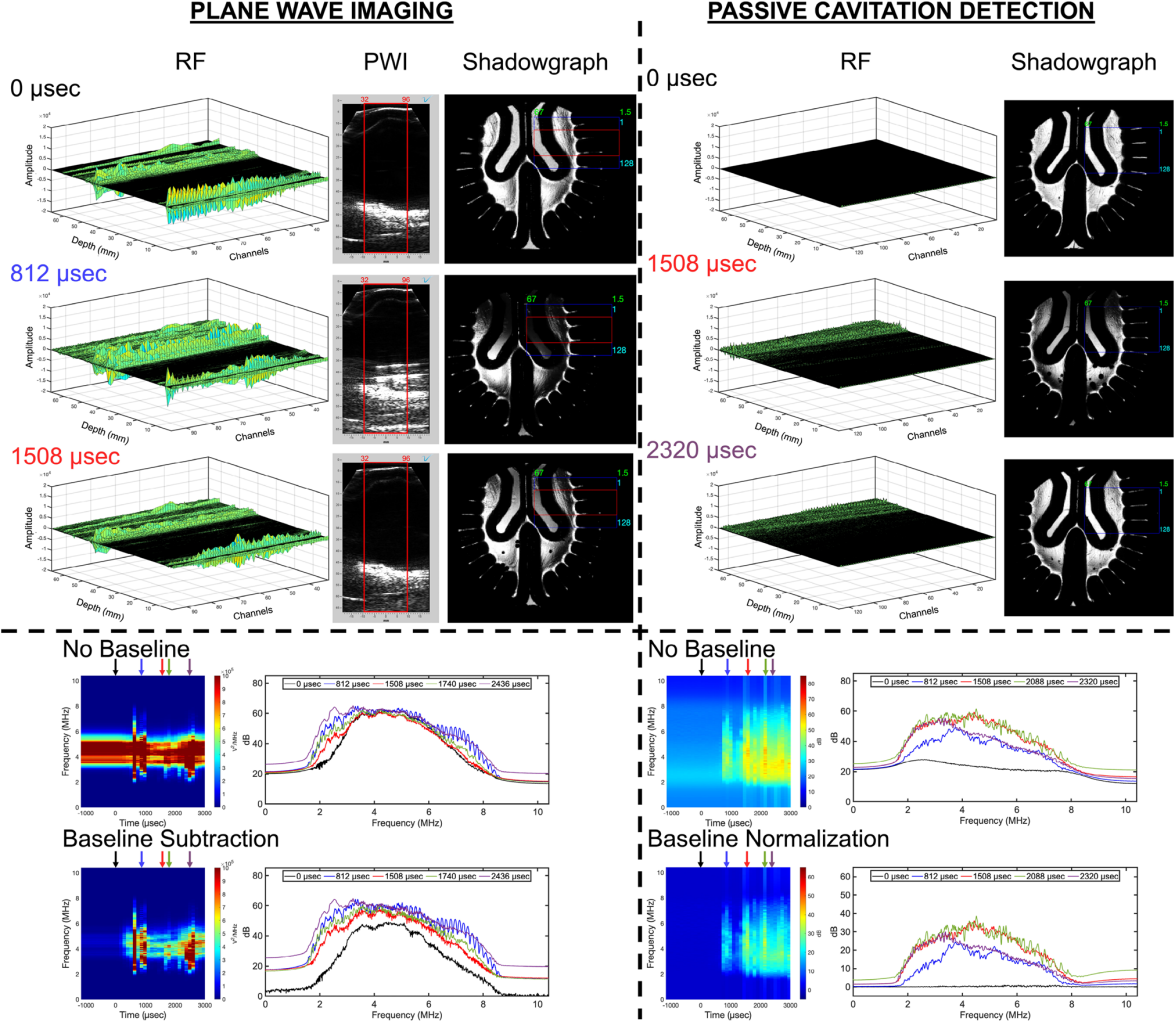
Comparison of plane wave imaging (PWI) and passive cavitation detection (PCD) for observations in the ventricles. A 3D representation of the radiofrequency (RF) data is presented, along with acoustic and shadowgraph imaging frames. The signal processing pipeline includes raw transformation into the frequency domain, baseline subtraction, and baseline normalization

**Fig. 7:**
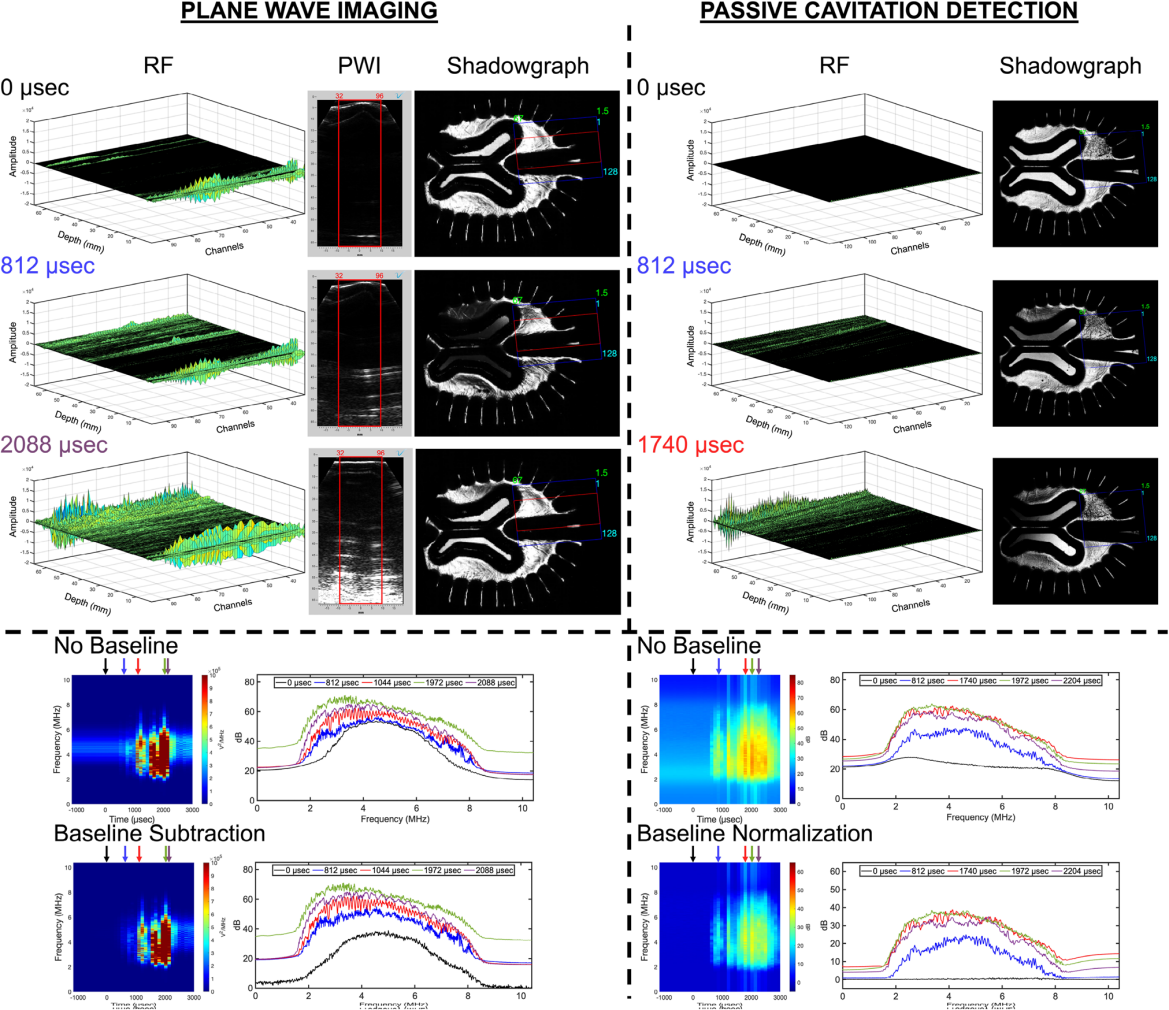
Comparison of plane wave imaging (PWI) and passive cavitation detection (PCD) for observations along the depth of the central sulcus. A 3D representation of the radiofrequency (RF) data is presented, along with acoustic and shadowgraph imaging frames. The signal processing pipeline includes transforming the raw data into the frequency domain, subtracting the baseline, and normalizing the baseline

When observing PWI and PCD, as shown in Figs. 5, 6, and 7, the RF data at the time of impact (0 µsec) reveal pre-existing scatterers within the head model, with amplitude variations occurring at different axial depths and lateral locations. In the contrecoup region, most of the reflections are observed at cortical sulci and near the central sulcus, suggesting the presence of pre-existing bubble nuclei. When observing the ventricles, reflections are primarily located at the tissue-fluid interface of the ventricular cavity. Lastly, minimal scatter is observed when viewing the central sulcus of the brain phantom, although some remains visible along its depth. All head models, however, exhibit pre-existing reflections at the interface between the transducer, coupling gel, and the standoff pad. All plane wave frames display high-intensity regions corresponding to acoustic scatter during bubble growth and collapse with reflections of the inner wall of the skull geometry. In Fig. 5, during areas of bubble growth shown in the shadowgraph (Supplementary Video 4), the RF data reveals planar wave fronts with amplification near the cortical sulci, while the plane wave frames further emphasize high-intensity regions associated with cavitation bubble growth and collapse, where acoustic artifacts are present. When examining the periventricular spaces (Supplementary Video 5), Figure 6 shows minimal changes in amplitude. However, it still contributes to high-intensity regions in the plane wave frames during limited bubble growth at the gel interface, where shockwave events are minimally detected due to the localization of bubble activity in the contrecoup region. Finally, when visualizing the central sulcus (Fig. 7 and Supplementary Video 6), high-intensity regions are observed during bubble collapse, particularly along the depth of the central sulcus extending to the ventricular cavity in both the hydrogel and free liquid, providing evidence of bubble growth. PCD follows a similar trend to PWI, except that there are no insonified pre-existing scatterers at 0 µsec. Additionally, the amplitude is reduced during bubble growth and collapse, compared to PWI, but remains more localized at depth.

### Plane Wave Imaging with Baseline Correction Highlights Spectral Changes

The PWI acoustic spectra reveal significant spectral changes in cavitation activity after baseline subtraction, highlighting features that were previously masked by pre-existing artifacts across all head models. The linear and logarithmic power spectra without baseline correction expose these scatterers, such as bubble sources trapped in the head model or the potential return trip of the transmitted plane wave, where energy is visible within ~3–6 MHz before impact. At first glance, the ventricles revealed the highest pre-existing content, which was followed by the contrecoup. The central sulcus is also evident through the RF plots. A transition between bubble collapse and shockwave generation becomes observable by selecting specific timestamps in the dB scale, validated through shadowgraph imaging. Notable jumps in energy occur starting from impact (0 µsec), particularly near 2.5–7.5 MHz and around the center where broadband signals increase across the entire frequency range. Baseline subtraction reduces the effects of pre-existing scatterers, making energy jumps more pronounced. Therefore, the contrecoup region shows the highest energy jumps, followed by the central sulcus and the ventricles when observing different frequencies during bubble growth and collapse, as shown in Table 1. Baseline normalization was performed on the PWI datasets (Supplementary Figures 7–12, 19–24, and 31–36) but was ultimately excluded, as it revealed reduced energy near the transducer’s center frequency, effectively suppressed by the normalization process, leaving energy only at 2 and 8 MHz across all head models.

**Table 1:** Cavitation thresholds based on plane wave imaging (PWI) baseline subtraction and passive cavitation detection (PCD) baseline normalization following blunt impact in ANGUS brain phantoms

Detection type	Cavitation phase	Frequency (MHz)	Contrecoup (dB)	Ventricles (dB)	Central sulcus (dB)
PWI		2.5	9.0 ± 15.5	22.7 ± 5.5	21.7 ± 9.5
	Growth	5.2	14.3 ± 9.5	12.3 ± 3.8	15.7 ± 4.7
		7.5	12.0 ± 8.7	14.7 ± 6.0	11.0 ± 6.9
		1.0	21.0 ± 4.4	20.7 ± 3.2	27.0 ± 7.8
	Collapse	5.2	22.3 ± 1.5	11.7 ± 2.1	26.0 ± 2.0
		9.0	23.7 ± 3.2	22.7 ± 4.5	24.0 ± 6.2
PCD		2.5	20.7 ± 10.0	16.0 ± 4.6	7.0 ± 8.7
	Growth	5.2	27.7 ± 8.1	19.7 ± 3.2	9.7 ± 12.5
		7.5	9.7 ± 9.0	4.3 ± 1.5	1.0 ± 1.7
		1.0	11.0 ± 3.0	6.7 ± 5.5	8.7 ± 2.1
	Collapse	5.2	43.7 ± 6.7	35.3 ± 10.2	34.3 ± 1.5
		9.0	17.0 ± 4.4	12.0 ± 9.5	11.3 ± 1.5

### Passive Cavitation Detection with Baseline Correction Highlights Broadband Energy

PCD demonstrated greater sensitivity in detecting changes in cavitation bubble dynamics, primarily broadband behavior, during the blunt impact test across all head models. Without baseline correction, the energy response at impact (0 µsec) appeared as near-uniform spectra, indicating minimal artifacts, as no transmit wave was present to insonify any pre-existing scatterer in the imaging field. Consequently, baseline subtraction was applied to the PCD datasets (Supplementary Figs. 1–6, 13–18, and 25–30) but was ultimately excluded, as it revealed minimal differences across all frequencies when compared to the non-baseline-corrected spectra. Therefore, baseline normalization was implemented to determine energy in bubble growth and collapse phases; however, broadband behavior was mainly observed during collapse, with minimal energy jumps during bubble growth. Table 1 presents the cavitation thresholds under baseline normalization using PCD, highlighting substantial energy changes in the contrecoup, ventricles, and the central sulcus. Among all head models, the contrecoup consistently exhibited the most significant energy differences, with similar findings observed when examining the ventricles and along the axis of the central sulcus.

### Spectral Analysis Allows Mapping of Cavitation Activity Through an STFT Approach

**Fig. 8:**
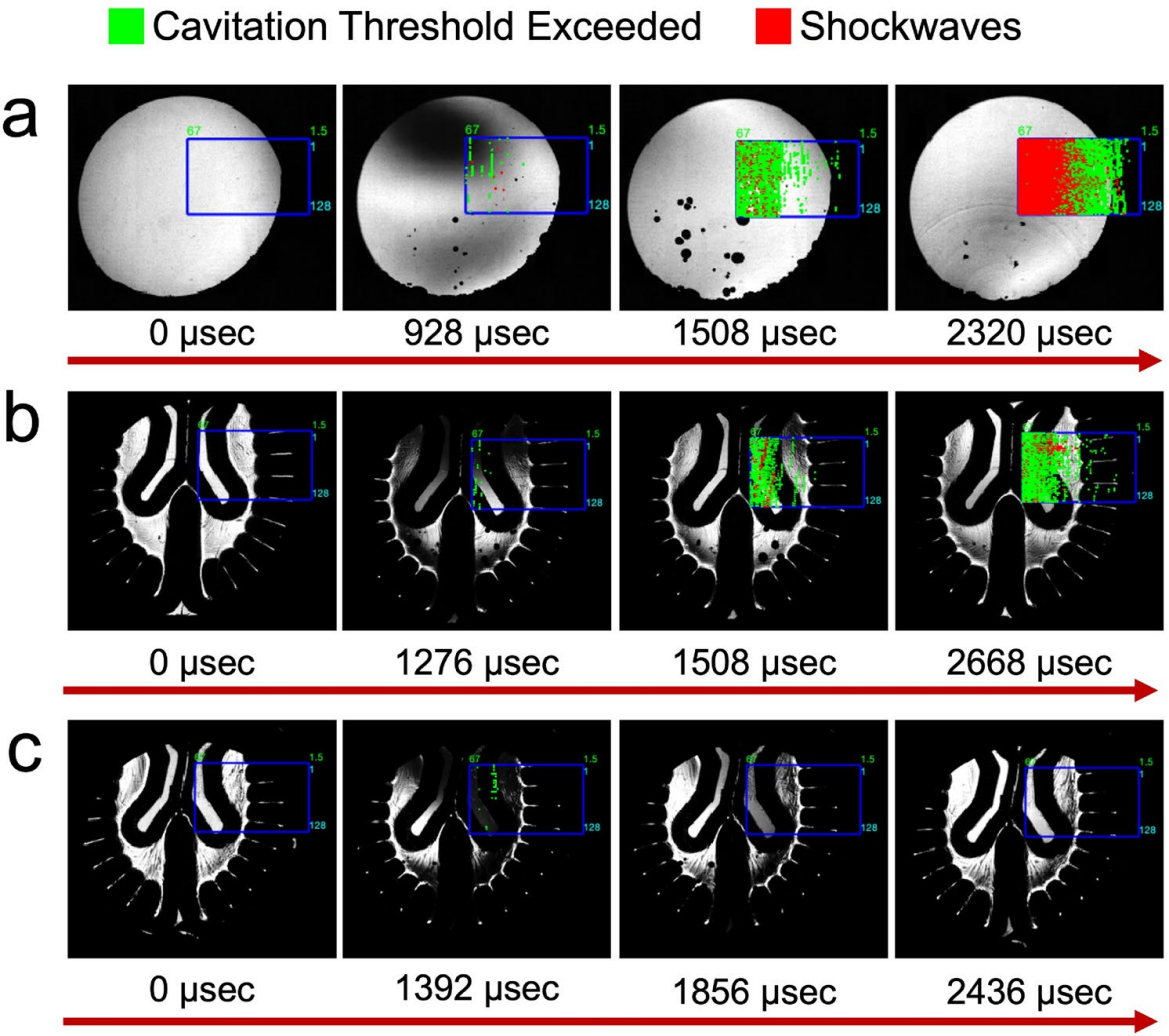
Cavitation mapping on shadowgraph frames was performed using a short-time Fourier transform (STFT) technique based on unique cavitation thresholds. Three head models were tested: **a** filled with deionized (DI) water, **b** an ANGUS brain phantom, and **c** an ANGUS brain phantom with a temporal bone skull geometry and all artificial dampeners. Green markers indicate cavitation activity, involving the general presence of bubbles, characterized by pressure differential fluctuations and energy spikes at frequencies (2.5, 5.2, and 7.5 MHz) within or near the transducer’s optimal receive bandwidth. In contrast, red markers represent shockwave emissions, which are evident with frequency components outside the acoustic transducers’ optimal receive bandwidth, specifically at 1.0 and 9.0 MHz, exceeding their respective thresholds

A short-time Fourier transform (STFT) was performed using previously defined parameters to map cavitation activity onto acoustic and optical frames as shown in Fig. 8 [31, 37]. The STFT was applied to the PCD datasets using the bubble growth and collapse thresholds listed in Table 1, specifically for ventricular observations. Green markers indicate cavitation activity, which involves the general presence of just bubble growth or a mixed phase of growth and collapse detected within or near the optimal transmit bandwidth of the transducer. A green marker appears when the 2.5, 5.2, and 7.5 MHz frequency components exceed their respective threshold values. In contrast, red markers signify more intense events, typically associated with shockwave emissions, where frequency components outside the optimal bandwidth, specifically at 1.0 and 9.0 MHz (with 5.2 MHz also considered), surpass their thresholds. Figure 8 illustrates a representative impact test showing these phenomena in head models filled with DI water and ANGUS brain phantoms. In the DI water model (Fig. 8a), green markers appear during the bubble growth phase, peaking at 928 and 1508 µsec, followed by a surge of red markers at 2320 µsec, indicating shockwave activity validated through the shadowgraph frames. A similar pattern is observed in the ANGUS phantom (Fig. 8b), where activity is concentrated around the ventricles, although fewer shockwaves are detected compared to the DI water model.

### Cavitation is Minimized with Integration of Artificial Dampeners

The design of the asymmetrical head model involved integrating artificial dampeners to replicate the behavior of human tissue under blunt impact conditions. To illustrate the contribution of each component, Supplementary Fig. 37 illustrates the effect of each dampener in DI water, allowing for a complete visualization of cavitation bubble dynamics. Supplementary Fig. 37a presents the 3D-printed asymmetrical skull geometry, where cavitation remains centralized within the head model; however, most bubbles are localized along the grooves, where shockwaves appear to propagate following the groove geometry. Supplementary Fig. 37b shows a similar test with the addition of a scalp mimic. While the cavitation pattern remains similar, the number of bubbles is reduced, and their radial expansion is less pronounced. Supplementary Fig. 37c demonstrates the further integration of a dura mater mimic, where bubbles are primarily observed along the surface of the dura, with minimal cavitation occurring in the bulk fluid. Supplementary Fig. 37d presents the integration of all dampeners onto the asymmetrical skull, which significantly reduces cavitation within the head model. Accordingly, Fig. 8c shows results for the same asymmetrical skull geometry but incorporating a temporal bone region, a dura mater mimic, a scalp layer, and an ANGUS brain phantom (Fig. 1d), demonstrating a transcranial approach to acoustic cavitation detection during impact. Notably, even with cavitation thresholds optimized for a head model with an acoustic window, cavitation is still detected to a limited extent at 1392 µsec (Supplementary Figs. 38 and 39), suggesting that the transcranial approach may be viable for real-world applications.

## Discussion

### Constraints in Modeling Anatomical and Mechanical Properties of the Human Head

All head models were previously designed and manufactured to replicate the geometric and anatomical characteristics of the human head in a simplified manner, inspired by a transcranial slice obtained through magnetic resonance imaging (MRI) [16, 31, 37]. The ANGUS brain phantom (Fig. 1a) features uniform gyri, ~ 1 mm sulcal widths, and ~ 1–5 mm spacing around the cranial model, forming an unconstrained structure with limited free-body motion. The ANGUS phantom is constructed from PAA, providing the optical transparency necessary for high-speed optical imaging, as well as a similar density (1.005 g/cc) and shear modulus comparable to that of human brain tissue. PAA has been previously characterized using parallel plate oscillatory rheometry and magnetic resonance elastography (MRE), both of which confirmed its linear viscoelastic behavior [37, 39, 40]. At 10 Hz, rheometry yielded a storage modulus of approximately 1800 Pa and a loss modulus of approximately 200 Pa, corresponding to stiffness and viscous values similar to ex vivo human brain tissue [43, 44]. However, MRE reveals a key limitation for the simplified phantom such that PAA shows lower attenuation at high shear rates compared to in vivo human brain tissue [39]. This limitation is significant because human brain tissue is heterogeneous due to its complex cellular and vascular matrix in both gray and white matter, which increases anisotropy and leads to a combined visco-poroelastic behavior under both low and high shear rates [45, 46]. Other brain phantoms are specifically engineered to replicate these visco-poroelastic characteristics by providing higher attenuation, thereby better mimicking tissue heterogeneity [47, 48]. However, this typically comes at the expense of optical transparency and the lack of both anatomical and geometrical features, making them unsuitable for cavitation visualization and validation at tissue-fluid interfaces.

The cranial geometry enables cavitation observation during impact, using different transducer ports to monitor tissue–fluid interfaces. The 3D-printed skull is a 2D extrusion of the human cranium, with volume (~1600 cc), density (1.25 g/cc), thickness (~8 mm), and flexural strength (~63 MPa) [31, 37] similar to human cranial bone. Although PLA can be used to achieve anatomical accuracy and mimic fracture patterns, it does not match the cranial bone’s flexural modulus or strain behavior under deformation, leading to different vibrational modes that may not replicate in vivo events [49, 50]. The drop tower apparatus has head models resting freely on a wooden block while receiving impacts of ~ 6.7 J. While these impacts are not intended to replicate any specific real-world scenario, this energy falls within the range of low-to-moderate blunt impacts, such as minor sports collisions, light falls, or small objects striking the head and matches previous blunt force studies [31, 37]. The blunt impact experiments are simplified such that the flat-bottom edge of the impactor strikes a small contact area of the curved skull geometry, no neck model is integrated to simulate realistic head motion, and the wooden block leads to unique skull displacements. The acoustic transducer is positioned to detect cavitation at the tissue-fluid interfaces. The first model receives acoustic emissions at the contrecoup, the most prominent suspected cavitation site [12, 16, 18, 19]. It includes features like the cortical gyrus and sulci, up to the central sulcus, where the linear array transducer is mounted at the midplane to avoid bubble responses that may pre-exist on the surfaces of the acrylic plates [31, 37]. The second model targets the periventricular spaces, as well as the associated cortical gyri and sulci, to determine if the initial impact pressure wave generates cavitation as it travels to the contrecoup, a region where cavitation is prone to occur with increasing degrees of overpressure [12, 19, 37]. Lastly, the third model is rotated 90° and impacted at its apex to visualize cavitation along the central sulcus depth, where optical observations serve as a comparison to the other head models impacted in a different orientation [16, 37].

### Optical Observations Highlight Effects of Impact Orientation on Cavitation Behavior

Blunt impacts on each head model revealed variations in pressure differentials and cavitation bubble activity. The models viewing the contrecoup and ventricles exhibited similar results, likely due to their comparable geometries and impact orientations. Although the placement of the transducer ports differed, these variations did not appear to affect the location and timing of cavitation bubble growth, as both models produced nearly identical outcomes. However, the onset of bubble collapse showed more variation, which can be attributed to differences in bubble quantity, expansion rates, and persistence. Including the ANGUS phantom did not significantly alter the timing of cavitation, suggesting minimal energy dissipation as the impact wave traveled through the head model and that the timing of the pressure differential remained unaffected; however, it did influence the time for complete bubble collapse and shock wave generation, indicating that the viscoelastic nature of the phantom prolonged the bubble collapse phase. While the contrecoup and ventricles showed similar results, they differed noticeably from the central sulcus model.

Observations in the central sulcus revealed altered pressure differentials and cavitation bubble activity as compared to the contrecoup and ventricles. The central sulcus was rotated 90° to explore the effects of an altered impact scenario, leading to the redefinition of the coup and contrecoup regions. This modification resulted in noticeable changes in both the timing and appearance of cavitation bubbles and shockwaves (Fig. 4d). These differences are likely due to the reduced travel time of the impact wave, which led to more rapid development of the pressure differential compared to the contrecoup and ventricles. Although the impact wave is not easily visualized in the shadowgraph images and a pressure transducer was not implemented to monitor the negative pressure profile, this claim can be supported through a simplified impact wave travel time calculation. Assuming the speed of sound in water (~1500 m/s) and an internal travel distance from coup to contrecoup of ~ 180 mm for the contrecoup and ventricles, and 135 mm for the central sulcus, the estimated impact wave travel times are ~ 120 and 90 µsec, respectively [51, 52]. A 30 µsec reduction in the central sulcus supports observing a more rapid pressure differential development and earlier onset of blunt-induced cavitation. Finally, while the timing of pressure differentials varies, the curvature of the head model geometry also influences how the pressure field is distributed within the bulk medium, further affecting cavitation dynamics.

The head models visualizing the contrecoup and ventricular cavity exhibit a smaller radius of curvature due to their concave nature, compared to the central sulcus orientation, which is impacted in a different orientation. This suggests that the negative pressure phase is amplified as the impact wave reflects off the skull’s curved interior, focusing and constructively interfering near the center of the head model, thereby inducing cavitation [53–55]. In contrast, the central sulcus experiences fewer reflections, resulting in a more diffuse negative pressure phase that leads to cavitation bubbles with reduced surface area and persistence. Similar to the impact wave, shockwaves generated by bubble collapse reflect and interfere in the same way. This phenomenon is particularly relevant in assessing brain injuries, where intensified local pressure regions can exacerbate neuro-cellular and vascular damage, leading to disruptions in motor function [12, 17]. However, these observations are challenging to validate using the ANGUS head model alone. Factors such as skull flexure, cranial morphology, and pre-existing bubble nuclei can lead to varied outcomes, requiring further experimentation [14, 31]. Additionally, the lack of depth perception in shadowgraph imaging complicates the distinction between surface-adhered bubbles and those within the bulk fluid. These limitations underscore the need to incorporate acoustic imaging techniques to verify and more accurately interpret cavitation behavior within the bulk fluid.

### Evaluating Acoustic Plane Wave and Passive Cavitation Methods for Cavitation Detection

An important limitation of this study is that intracranial pressure (ICP) was not directly measured within the phantom. Although these models reproduce relevant material properties and enable controlled cavitation experiments, their non-biological nature prevents them from capturing physiologically realistic ICP, which in vivo typically ranges from approximately 5–15 mmHg and can increase or decrease under pathological conditions over time, particularly following brain injury where ICP dynamics may change due to swelling, edema, or altered cerebral compliance [56, 57]. In recent work using the ANGUS phantom under similar impact loading, ICP was measured with a baseline (gauge) pressure of 0 mmHg, from which the incident and reflected shockwave profiles were recorded instantaneously, approximately 5–15 mmHg lower than physiological ICP [16]. This pressure difference may alter cavitation thresholds and should therefore be considered when translating these findings to in vivo, in vitro, or in silico environments. Pressurizing the head model to approximate in vivo ICP may be feasible; however, even if similar absolute ICP levels were achieved, the resulting pressure gradients would likely not accurately represent those in the human head, where ICP is regulated by venous flow, the incompressibility of brain tissue, and the rigid cranial boundary. This limitation underscores the importance of developing alternative measurement strategies to better approximate physiological conditions, particularly acoustic techniques that rely on detecting bubble presence driven by pressure gradients, rather than pressure magnitude alone.

In most instances, ICP can indicate the presence of injury; however, an acoustic approach offers a more localized assessment with advantages over traditional methods. In clinical settings, traditional techniques may be invasive, such as catheter-based monitoring, or noninvasive, such as imaging approaches like Doppler ultrasound or MRI [56, 57]. Moreover, most ex vivo or surrogate head models rely on noninvasive integration, which can complicate studies when measuring or monitoring ICP [16, 42]. Acoustic methods, by comparison, detect rapid, transient, and localized bubble expansions through harmonic and broadband spectral signatures that depend on cavitation bubble dynamics during blunt impact. Although both traditional pressure sensors and acoustic techniques provide enhanced spatial and temporal resolution for detecting localized mechanical events, key differences exist. Traditional pressure sensors measure overall pressure at discrete locations, with limited spatial coverage, slower temporal resolution, and no capacity to generate real-time accurate image profiles; they are also often invasive, further complicating experimental models. Acoustic methods such as PWI and PCD, however, can noninvasively capture microsecond-scale, spatially distributed cavitation events in real time, and PWI additionally enables real-time image reconstruction, providing spatial and temporal landmarks within the phantom’s anatomical structure that can be tracked. Each approach carries distinct advantages and challenges that must be carefully considered when interpreting signals and applying them to complex head models, as even acoustic techniques face limitations at highly localized scales.

PWI has limitations in detecting cavitation in human head models, which may pose challenges if integrated into an in vivo system. Firstly, PWI is unique in its ability to transmit a planar wave that insonifies bubble sources, with reflected echoes detected to confirm bubble presence through spectral signatures at relatively high frame rates. Although the received data are averaged, thereby diminishing the distinct spectral features of individual bubbles that would be more apparent in single-channel analysis, detecting energy changes across the full lateral width remains valuable, ultimately supporting the identification of cavitation events through energy differences during blunt impact. However, a drawback arises from the nature of the transmitted plane wave, which produces strong reflections and attenuation along the inner surface of the skull due to impedance mismatches [58, 59]. These reflections result in elevated energy concentrations before and during impact, manifesting as high-intensity regions in both the reconstructed frames and power spectra, thereby complicating the assessment of true cavitation events. These effects are particularly evident when observing the ventricles and the contrecoup region but are less pronounced in the central sulcus model, where the return trip of the wave may be missed due to the longer travel distance. Baseline subtraction helps reduce the influence of pre-existing scatterers insonified by the transmitted wave across all head models, allowing for the extraction of spectral features that might otherwise be masked. However, this approach introduces a key limitation such that it may overestimate the energy jumps between the bubble growth and collapse phases. This happens because baseline subtraction reduces the signal before impact but does not remove the actual pre-existing scatterers. As a result, the energy jumps shown in Table 1 may be larger than what would be seen in an in vivo system. Nonetheless, while the ANGUS head models have their complications, the human brain is suspected to contain small amounts of air within the CSF and cortical sulci, which may also contribute to cavitation and exhibit similar trends if PWI were applied to a realistic TBI scenario [12, 16]. In this context, PWI may offer a more viable path for in vivo applications.

PCD offers a more effective approach for detecting cavitation by addressing the limitations of PWI. However, because PCD does not rely on a transmitted plane wave, detecting pre-existing scatterers is challenging, particularly in human head models, where pre-existing bubble nuclei are not visible to the naked eye. Although, this limitation can be mitigated by integrating high-quality acoustic imaging before impact to identify potential scatterers. Moreover, passive cavitation mapping is not readily applicable to blunt impact-induced cavitation, as key parameters, such as the driving frequencies of the bubbles, are unknown, and the bubbles are not confined to specific depths where time-of-flight (TOF) calculations could approximate energy gradients. Instead, bubbles are sporadically insonified by nearby shockwaves for brief durations, with most PCD energy acquisition occurring during bubble collapse. To assess energy variations during impact, baseline normalization was employed to enhance the visualization of broadband signals, including those potentially associated with bubble growth. All head models effectively detected shockwaves, which is attributed to their broadband spectral characteristics. The most pronounced signals were observed in the contrecoup region, primarily due to concentrated pressure differentials, followed by notable activity near the central sulcus and ventricles. To further evaluate the feasibility of PCD, the cavitation response was mapped to confirm the occurrence of cavitation.

### Effect of Artificial Dampeners on Cavitation Behavior During Blunt Impact

A head model incorporating artificial biological dampeners was developed as a proof-of-concept approach to more accurately simulate and visualize cavitation bubble dynamics during blunt trauma scenarios, aimed at a realistic PCD application. To enhance anatomical realism, the model included uneven surfaces and varying skull thicknesses, eliminating the symmetrical geometry of previous models and more accurately representing the human skull [37, 60, 61]. The meninges were partially replicated, specifically the dura mater, together with the falx cerebri, both of which are known to help mitigate brain injury during impact [62, 63]. A scalp layer was also fabricated with mechanical properties similar to those of the human scalp, and its thickness was selected to fall within the median age range of military personnel, aiming to better simulate impact absorption [42, 64]. To identify how each dampener influences cavitation, each component was tested individually. When the skull was filled with DI water alone (Supplementary Video 7), cavitation was still observed in the contrecoup region, where bubbles and shockwaves are localized in the grooves. These grooves are prominent sites for bubble nuclei, similar to those involved in acoustic kidney stone detection, where microsized bubble nuclei reside in grooves [65]. This contrasts with earlier models, where smoother internal geometries led to more uniform shockwave reflection, suggesting that local energy differentials are likely to be present under blunt trauma. Adding the scalp layer (Supplementary Video 8) preserved cavitation in the contrecoup region but resulted in reduced radial bubble expansion and faster collapse times. This suggests that the scalp dampens pressure differentials, limiting bubble growth without eliminating cavitation. The dura mater mimic exhibited unique behavior (Supplementary Video 9), with cavitation developing primarily along the dura surface rather than in the surrounding DI water. This behavior may be attributed to the hydrophobic nature of the tin-cured silicone rubber, which does not fully replicate the properties of biological extracellular dura mater but may mimic similar responses under blunt impact conditions. When both the scalp and dura mater layers were combined (Supplementary Video 10), cavitation was significantly reduced. Only small bubbles appeared on the dura surface, and they failed to collapse with sufficient force to generate shockwaves.

Mapping of the cavitation response was conducted using PCD as a transcranial method for cavitation detection. It is essential to note that the mapping was simplified to spatial approximations based on the Verasonics hardware, which approximated the receive sequence according to the desired user-defined acquisition frame rate. Consequently, no TOF corrections were applied based on the skull geometry dimensions or the approximate locations of bubbles. Nonetheless, acoustic transmission through the PLA-based human skull continues to pose significant challenges for acoustic imaging, which is why PCD was used in this scenario instead of PWI [58, 59]. Although the mapping employed thresholds (Table 1) designed for an acoustic window with symmetrical geometry, cavitation was still successfully detected, though to a limited extent, using a modified head model incorporating asymmetrical surfaces, artificial dampeners, and an ANGUS phantom. While the head model remains simplistic and the linear array transducer is mounted externally on the skull geometry, the results demonstrate that structural modifications can make the model viable for more realistic experimentation. Additionally, it is important to recognize that, due to the model’s simplified nature and lack of a human neck replica, it does not allow for capturing standard kinematic injury metrics, such as linear or rotational acceleration, making it difficult to translate acoustic findings to other injury metrics presented in more complex models. Nevertheless, the timing of cavitation dynamics observed here aligns well with kinematic events reported in similar models, indicating a potential relationship between cavitation activity and biomechanically relevant injury phases [16]. This highlights the need for future studies to combine acoustic cavitation detection with direct kinematic measurements to better validate cavitation as an injury biomarker.

In this context, acoustic PCD provides a versatile platform for implementation in in vitro models, offering insights that can inform and refine protocols in contact sports and military training. With further experimentation, it may also be adapted into wearable systems, aligning with current helmet modifications where sensors are being integrated for both civilian and military use [66, 67]. This includes helmets for sports such as American football, rugby, and ice hockey, where brain injuries are common, as well as protective equipment for activities that do not traditionally employ helmets [68]. Effective translation to real-world scenarios, however, requires addressing challenges such as skull-induced acoustic attenuation, limited acoustic windows, the need for compact and conformal transducer arrays, and user comfort. Strategies such as employing lower-frequency ultrasound for improved skull penetration, as in transcranial Doppler for cerebral blood flow monitoring [69, 70], developing advanced signal processing approaches for aberration correction, and ensuring reliable transducer coupling in dynamic environments represent critical areas for further development. Additional research is also needed to optimize cavitation detection thresholds across different blunt TBI scenarios and impact orientations, particularly under field conditions where consistent coupling is essential. Ultimately, PCD could provide valuable insights into how helmets and other protective gear may be designed to better deflect external impacts while minimizing intracranial pressure differentials that drive cavitation and brain injury.

## Supplementary Information

Below is the link to the electronic supplementary material.

Supplementary Video 1 (MOV 13157 KB)

Supplementary Video 2 (MOV 18832 KB)

Supplementary Video 3 (MOV 30214 KB)

Supplementary Video 4 (MOV 29057 KB)

Supplementary Video 5 (MOV 12451 KB)

Supplementary Video 6 (MOV 25772 KB)

Supplementary Video 7 (MOV 16554 KB)

Supplementary Video 8 (MOV 21808 KB)

Supplementary Video 9 (MOV 30115 KB)

Supplementary Video 10 (MOV 12185 KB)

Supplementary Video 11 (MOV 22409 KB)

Supplementary file1 (PDF 507398 kb)

## References

[1] Dewan, M. C., et al. Estimating the global incidence of traumatic brain injury. *J. Neurosurg.* 130(4):1080–1097, 2019. 10.3171/2017.10.JNS17352. 29701556 10.3171/2017.10.JNS17352

[2] Guan, B., D. B. Anderson, L. Chen, S. Feng, and H. Zhou. Global, regional and national burden of traumatic brain injury and spinal cord injury, 1990–2019: a systematic analysis for the Global Burden of Disease Study 2019. *BMJ Open*. 2023. 10.1136/bmjopen-2023-075049. 37802626 10.1136/bmjopen-2023-075049 PMC10565269

[3] Lindberg, M. A., E. M. Moy Martin, and D. W. Marion. Military traumatic brain injury: the history, impact, and future. *J. Neurotrauma*. 39(17–18):1133–1145, 2022. 35451333 10.1089/neu.2022.0103 PMC9422790

[4] Agimi, Y., L. Earyes, T. Deressa, and K. Stout. Estimating repeat traumatic brain injury in the U.S. Military, 2015–2017. *Mil. Med.* 187(3–4):E360–E367, 2022. 10.1093/milmed/usab041. 33591307 10.1093/milmed/usab041

[5] Swanson, T. M., B. M. Isaacson, C. M. Cyborski, L. M. French, J. W. Tsao, and P. F. Pasquina. Traumatic brain injury incidence, clinical overview, and policies in the US military health system since 2000. *Public Health Rep.* 132(2):251–259, 2017. 10.1177/0033354916687748. 28135424 10.1177/0033354916687748 PMC5349478

[6] Bass, C. R., M. B. Panzer, K. A. Rafaels, G. Wood, J. Shridharani, and B. Capehart. Brain injuries from blast. *Ann. Biomed. Eng.* 40(1):185–202, 2012. 10.1007/s10439-011-0424-0. 22012085 10.1007/s10439-011-0424-0

[7] Wallace, D. Improvised explosive devices and traumatic brain injury: the military experience in Iraq and Afghanistan. *Australas. Psychiatry*. 17(3):218–224, 2009. 10.1080/10398560902878679. 19404818 10.1080/10398560902878679

[8] Lindquist, L. K., H. C. Love, and E. B. Elbogen. Traumatic brain injury in Iraq and Afghanistan veterans: new results from a national random sample study. *J. Neuropsychiatry Clin. Neurosci.* 29(3):254–259, 2017. 10.1176/appi.neuropsych.16050100. 28121256 10.1176/appi.neuropsych.16050100 PMC5501743

[9] Warden, D. Military TBI during the Iraq and Afghanistan wars. *J. Head Trauma Rehabil.* 21(5):398, 2006. 16983225 10.1097/00001199-200609000-00004

[10] Bryden, D. W., J. I. Tilghman, and S. R. Hinds II. Blast-related traumatic brain injury: current concepts and research considerations. *J. Exp. Neurosci.* 13:1179069519872213, 2019. 10.1177/1179069519872213. 31548796 10.1177/1179069519872213 PMC6743194

[11] Shively, S. B., I. Horkayne-Szakaly, R. V. Jones, J. P. Kelly, R. C. Armstrong, and D. P. Perl. Characterisation of interface astroglial scarring in the human brain after blast exposure: a post-mortem case series. *Lancet Neurol.* 15(9):944–953, 2016. 10.1016/S1474-4422(16)30057-6. 27291520 10.1016/S1474-4422(16)30057-6

[12] Marsh, J. L., and S. A. Bentil. Cerebrospinal fluid cavitation as a mechanism of blast-induced traumatic brain injury: a review of current debates, methods, and findings. *Front. Neurol.* 2021. 10.3389/fneur.2021.626393. 33776887 10.3389/fneur.2021.626393 PMC7994250

[13] Finan, J. D., T. E. Vogt, and Y. Samei. Cavitation in blunt impact traumatic brain injury. *Exp. Fluids*. 65(8):114, 2024. 10.1007/s00348-024-03853-6. 39036013 10.1007/s00348-024-03853-6 PMC11255084

[14] Salzar, R. S., D. Treichler, A. Wardlaw, G. Weiss, and J. Goeller. Experimental investigation of cavitation as a possible damage mechanism in blast-induced traumatic brain injury in post-mortem human subject heads. *J. Neurotrauma*. 34(8):1589–1602, 2017. 10.1089/neu.2016.4600. 27855566 10.1089/neu.2016.4600

[15] Haniff, S., and P. A. Taylor. In silico investigation of blast-induced intracranial fluid cavitation as it potentially leads to traumatic brain injury. *Shock Waves*. 27(6):929–945, 2017. 10.1007/s00193-017-0765-1.

[16] Kerwin, J., et al. Sulcal cavitation in linear head acceleration: possible correlation with chronic traumatic encephalopathy. *Front. Neurol.* 2022. 10.3389/fneur.2022.832370. 35295830 10.3389/fneur.2022.832370 PMC8918564

[17] Nakagawa, A., et al. Mechanisms of primary blast-induced traumatic brain injury: Insights from shock-wave research. *J. Neurotrauma*. 28(6):1101–1119, 2011. 10.1089/neu.2010.1442. 21332411 10.1089/neu.2010.1442

[18] (郎骥) Lang, J., R. Nathan, D. (周东) Zhou, X. (张雪薇) Zhang, B. (李波) Li, and Q. (吴千红) Wu. Cavitation causes brain injury. *Phys. Fluids*. 33(3):031908, 2021. 10.1063/5.0041139.

[19] Miller, S. T., C. F. Cooper, P. Elsbernd, J. Kerwin, R. Mejia-Alvarez, and A. M. Willis. Localizing clinical patterns of blast traumatic brain injury through computational modeling and simulation. *Front. Neurol.* 2021. 10.3389/fneur.2021.547655. 34093380 10.3389/fneur.2021.547655 PMC8173077

[20] Herbert, E., S. Balibar, and F. Caupin. Cavitation pressure in water. *Phys. Rev. E*. 74(4):41603, 2006. 10.1103/PhysRevE.74.041603. 10.1103/PhysRevE.74.041603 17155066

[21] Panzer, M. B., B. S. Myers, B. P. Capehart, and C. R. Bass. Development of a finite element model for blast brain injury and the effects of CSF cavitation. *Ann. Biomed. Eng.* 40(7):1530–1544, 2012. 10.1007/s10439-012-0519-2. 22298329 10.1007/s10439-012-0519-2

[22] Monson, K. L., M. I. Converse, and G. T. Manley. Cerebral blood vessel damage in traumatic brain injury. *Clin. Biomech.* 64:98–113, 2019. 10.1016/j.clinbiomech.2018.02.011. 10.1016/j.clinbiomech.2018.02.011 29478776

[23] Shively, S. B., and D. P. Perl. Traumatic brain injury, shell shock, and posttraumatic stress disorder in the military-past, present, and future. *J. Head Trauma Rehabil.* 2012. 10.1097/HTR.0b013e318250e9dd. 22573042 10.1097/HTR.0b013e318250e9dd

[24] Elliott, J., and J. C. Simon. Histotripsy bubble dynamics in elastic, anisotropic tissue-mimicking phantoms. *Ultrasound Med. Biol.* 49(3):853–865, 2023. 10.1016/j.ultrasmedbio.2022.11.012. 36577567 10.1016/j.ultrasmedbio.2022.11.012 PMC9908827

[25] Luo, J., and Z. Niu. Jet and shock wave from collapse of two cavitation bubbles. *Sci. Rep.* 2019. 10.1038/s41598-018-37868-x. 30718594 10.1038/s41598-018-37868-x PMC6362243

[26] Kang, W., A. Adnan, T. O’Shaughnessy, and A. Bagchi. Cavitation nucleation in gelatin: experiment and mechanism. *Acta Biomater.* 67:295–306, 2018. 10.1016/j.actbio.2017.11.030. 29191509 10.1016/j.actbio.2017.11.030

[27] Bader, K. B., E. Vlaisavljevich, and A. D. Maxwell. For Whom the Bubble Grows: Physical Principles of Bubble Nucleation and Dynamics in Histotripsy Ultrasound Therapy. Amsterdam: Elsevier, 2019. 10.1016/j.ultrasmedbio.2018.10.035. 10.1016/j.ultrasmedbio.2018.10.035 PMC6524960 30922619

[28] Izadifar, Z., P. Babyn, and D. Chapman. Ultrasound Cavitation/Microbubble Detection and Medical Applications. Berlin: Springer, 2019. 10.1007/s40846-018-0391-0.

[29] Settles, G. S., and M. J. Hargather. A Review of Recent Developments in Schlieren and Shadowgraph Techniques. Bristol: Institute of Physics Publishing, 2017. 10.1088/1361-6501/aa5748.

[30] Song, J. H., K. Johansen, and P. Prentice. An analysis of the acoustic cavitation noise spectrum: the role of periodic shock waves. *J. Acoust. Soc. Am.* 140(4):2494–2505, 2016. 10.1121/1.4964633. 27794293 10.1121/1.4964633

[31] Galindo, E. J., R. R. Flores, R. Mejia-Alvarez, A. M. Willis, and M. S. Tartis. Simultaneous high-frame-rate acoustic plane-wave and optical imaging of intracranial cavitation in polyacrylamide brain phantoms during blunt force impact. *Bioengineering*. 2024. 10.3390/bioengineering11020132. 38391618 10.3390/bioengineering11020132 PMC11605226

[32] Yang, Y., et al. Cavitation dose painting for focused ultrasound-induced blood-brain barrier disruption. *Sci. Rep.* 2019. 10.1038/s41598-019-39090-9. 30808897 10.1038/s41598-019-39090-9 PMC6391404

[33] Haworth, K. J., K. B. Bader, K. T. Rich, C. K. Holland, and T. D. Mast. Quantitative frequency-domain passive cavitation imaging. *IEEE Trans. Ultrason. Ferroelectr. Freq. Control*. 64(1):177–191, 2017. 10.1109/TUFFC.2016.2620492. 27992331 10.1109/TUFFC.2016.2620492 PMC5344809

[34] Tanter, M., and M. Fink. Ultrafast imaging in biomedical ultrasound. *IEEE Trans. Ultrason. Ferroelectr. Freq. Control*. 61(1):102–119, 2014. 10.1109/TUFFC.2014.2882. 24402899 10.1109/TUFFC.2014.6689779

[35] Montaldo, G., M. Tanter, J. Bercoff, N. Benech, and M. Fink. Coherent plane-wave compounding for very high frame rate ultrasonography and transient elastography. *IEEE Trans. Ultrason. Ferroelectr. Freq. Control*. 56(3):489–506, 2009. 10.1109/TUFFC.2009.1067. 19411209 10.1109/TUFFC.2009.1067

[36] Xu, Z., T. L. Hall, E. Vlaisavljevich, and F. T. Lee Jr. Histotripsy: the first noninvasive, non-ionizing, non-thermal ablation technique based on ultrasound. *Int. J. Hyperthermia*. 38(1):561–575, 2021. 10.1080/02656736.2021.1905189. 33827375 10.1080/02656736.2021.1905189 PMC9404673

[37] E. J. Galindo, Acoustic and Optical Imaging of Intracranial Cavitation in Polyacrylamide Human Head Models for Blunt Impact Traumatic Brain Injury Studies. New Mexico Institute of Mining and Technology ProQuest Dissertations & Theses, 2024. 31637777, Socorro, NM, USA, 2024.

[38] Wermer, A., J. Kerwin, K. Welsh, R. Mejia-Alvarez, M. Tartis, and A. Willis. Materials characterization of cranial simulants for blast-induced traumatic brain injury. *Mil. Med.* 2020. 10.1093/milmed/usz228. 32074306 10.1093/milmed/usz228

[39] Knutsen, A. K., et al. Characterization of material properties and deformation in the ANGUS phantom during mild head impacts using MRI. *J. Mech. Behav. Biomed. Mater.*138:105586, 2023. 10.1016/j.jmbbm.2022.105586. 36516544 10.1016/j.jmbbm.2022.105586 PMC10169236

[40] Baker, A. J. A., et al. Mechanical characterization data of polyacrylamide hydrogel formulations and 3D printed PLA for application in human head phantoms. *Data Br.* 48(109114):1–14, 2023. 10.1016/j.dib.2023.109114. 10.1016/j.dib.2023.109114 PMC10130753 37122918

[41] Deuflhard, P., O. Dössel, A. K. Louis, and S. Zachow. More mathematics into medicine! In: Production Factor Mathematics, Berlin: Springer, 2010, pp. 357–378. 10.1007/978-3-642-11248-5_19.

[42] Li, Y., et al. Influence of surrogate scalp material and thickness on head impact responses: toward a biofidelic head-brain physical model. *J. Mech. Behav. Biomed. Mater.*142:105859, 2023. 10.1016/j.jmbbm.2023.105859. 37071964 10.1016/j.jmbbm.2023.105859

[43] Chatelin, S., A. Constantinesco, and R. Willinger. Fifty years of brain tissue mechanical testing: from in vitro to in vivo investigations. *Biorheology*. 2010. 10.3233/BIR-2010-0576. 21403381 10.3233/BIR-2010-0576

[44] Forte, A. E., S. M. Gentleman, and D. Dini. On the characterization of the heterogeneous mechanical response of human brain tissue. *Biomech. Model. Mechanobiol.* 16(3):907–920, 2017. 10.1007/s10237-016-0860-8. 27933417 10.1007/s10237-016-0860-8 PMC5422507

[45] Su, L., et al. Distinguishing poroelasticity and viscoelasticity of brain tissue with time scale. *Acta Biomater.* 155:423–435, 2023. 10.1016/j.actbio.2022.11.009. 36372152 10.1016/j.actbio.2022.11.009

[46] Greiner, A., et al. Poro-viscoelastic effects during biomechanical testing of human brain tissue. *Front. Mech. Eng.* 2021. 10.3389/fmech.2021.708350.

[47] Eckstein, K. N., et al. Mechanically anisotropic phantoms for magnetic resonance elastography. *Magn. Reson. Med.* 2024. 10.1002/mrm.30394. 39627953 10.1002/mrm.30394 PMC11893262

[48] Williams, L. T., Z. Cao, A. H. Lateef, M. D. J. McGarry, E. A. Corbin, and C. L. Johnson. Viscoelastic polyacrylamide MR elastography phantoms with tunable damping ratio independent of shear stiffness. *J. Mech. Behav. Biomed. Mater.*154:106522, 2024. 10.1016/j.jmbbm.2024.106522. 38537609 10.1016/j.jmbbm.2024.106522 PMC11023745

[49] Yang, B., et al. Frequency spectrum of the human head–neck to mechanical vibrations. *J. Low Freq. Noise Vib. Act. Control*. 37(3):611–618, 2017. 10.1177/1461348417747179.

[50] Mantecón, R., M. Marco, A. Muñoz-Sanchez, G. Youssef, J. Díaz-Álvarez, and H. Miguélez. Additive manufacturing and mechanical characterization of PLA-based skull surrogates. *Polymers*. 2023. 10.3390/polym15010058. 10.3390/polym15010058 PMC9824150 36616407

[51] Leighton, T. G. What is ultrasound? *Prog. Biophys. Mol. Biol.* 93(1–3):1–81, 2007. 10.1016/j.pbiomolbio.2006.07.026. 10.1016/j.pbiomolbio.2006.07.026 17045633

[52] Hughes, S. Medical ultrasound imaging. *Phys. Educ.* 36(6):468–475, 2001. 10.1088/0031-9120/36/6/304.

[53] Cooper, P. W. Explosives Engineering. Hoboken: Wiley, 1996.

[54] Požar, T., V. Agrež, and R. Petkovšek. Laser-induced cavitation bubbles and shock waves in water near a concave surface. *Ultrason. Sonochem.* 2021. 10.1016/j.ultsonch.2020.105456. 33517094 10.1016/j.ultsonch.2020.105456 PMC7844577

[55] Skews, B. W., and H. Kleine. Shock wave interaction with convex circular cylindrical surfaces. *J. Fluid Mech.* 654:195–205, 2010. 10.1017/S0022112010001151.

[56] Evensen, K. B., and P. K. Eide. Measuring intracranial pressure by invasive, less invasive or non-invasive means: limitations and avenues for improvement. *Fluids Barriers CNS*. 2020. 10.1186/s12987-020-00195-3. 32375853 10.1186/s12987-020-00195-3 PMC7201553

[57] De Moraes, F. M., and G. S. Silva. Noninvasive intracranial pressure monitoring methods: a critical review. *Associacao Arquivos de Neuro-Psiquiatria*. 2021. 10.1590/0004-282X-ANP-2020-0300. 10.1590/0004-282X-ANP-2020-0300 PMC9394557 34161530

[58] Riis, T. S., T. D. Webb, and J. Kubanek. Acoustic properties across the human skull. *Ultrasonics*.119:106591, 2022. 10.1016/j.ultras.2021.106591. 34717144 10.1016/j.ultras.2021.106591 PMC8642838

[59] Pinton, G., J.-F. Aubry, E. Bossy, M. Muller, M. Pernot, and M. Tanter. Attenuation, scattering, and absorption of ultrasound in the skull bone. *Med. Phys.* 39(1):299–307, 2012. 10.1118/1.3668316. 22225300 10.1118/1.3668316

[60] Alexander, S. L., K. Rafaels, C. A. Gunnarsson, and T. Weerasooriya. Structural analysis of the frontal and parietal bones of the human skull. *J. Mech. Behav. Biomed. Mater.* 90:689–701, 2019. 10.1016/j.jmbbm.2018.10.035. 30530225 10.1016/j.jmbbm.2018.10.035

[61] McElhaney, J. H., J. L. Fogle, J. W. Melvin, R. R. Haynes, V. L. Roberts, and N. M. Alem. Mechanical properties of cranial bone. *J. Biomech.* 3(5):495–511, 1970. 10.1016/0021-9290(70)90059-X. 5000416 10.1016/0021-9290(70)90059-x

[62] Dasgupta, K., and J. Jeong. Developmental biology of the meninges. *Genesis*. 57(5):e23288, 2019. 10.1002/dvg.23288. 30801905 10.1002/dvg.23288 PMC6520190

[63] Gu, L., M. S. Chafi, S. Ganpule, and N. Chandra. The influence of heterogeneous meninges on the brain mechanics under primary blast loading. *Compos. B Eng.* 43(8):3160–3166, 2012. 10.1016/j.compositesb.2012.04.014.

[64] Hori, H., G. Moretti, A. Rebora, and F. Crovato. The thickness of human scalp: normal and bald. *J. Investig. Dermatol.* 58(6):396–399, 1972. 10.1111/1523-1747.ep12540633. 5030661 10.1111/1523-1747.ep12540633

[65] Simon, J. C., O. A. Sapozhnikov, W. Kreider, M. Breshock, J. C. Williams, and M. R. Bailey. The role of trapped bubbles in kidney stone detection with the color Doppler ultrasound twinkling artifact. *Phys. Med. Biol.* 2018. 10.1088/1361-6560/aa9a2f. 29131810 10.1088/1361-6560/aa9a2f PMC5791757

[66] Seshadri, D. R., et al. Wearable sensors for monitoring the internal and external workload of the athlete. *NPJ Dig. Med.* 2019. 10.1038/s41746-019-0149-2. 10.1038/s41746-019-0149-2 PMC6662809 31372506

[67] Hu, Z., et al. 3-D transcranial microbubble cavitation localization by four sensors. *IEEE Trans. Ultrason. Ferroelectr. Freq. Control*. 68(11):3336–3346, 2021. 10.1109/TUFFC.2021.3091950. 34166187 10.1109/TUFFC.2021.3091950 PMC8808337

[68] Sahler, C. S., and B. D. Greenwald. Traumatic brain injury in sports: a review. *Rehabil. Res. Pract.* 2012:1–10, 2012. 10.1155/2012/659652. 10.1155/2012/659652 PMC3400421 22848836

[69] Naqvi, J., K. H. Yap, G. Ahmad, and J. Ghosh. Transcranial Doppler ultrasound: a review of the physical principles and major applications in critical care. *Int. J. Vasc. Med.* 2013. 10.1155/2013/629378. 24455270 10.1155/2013/629378 PMC3876587

[70] Pan, Y., W. Wan, M. Xiang, and Y. Guan. Transcranial Doppler ultrasonography as a diagnostic tool for cerebrovascular disorders. *Front. Media S.A.* 2022. 10.3389/fnhum.2022.841809. 10.3389/fnhum.2022.841809 PMC9101315 35572008

