# Supplementary material for: Acoustic Detection of Intracranial Cavitation Induced by Blunt Impacts in Polyacrylamide Human Head Models Across Varying Orientations
Source: Ann Biomed Eng. 2025 Nov 23;54(2):621–39. doi: 10.1007/s10439-025-03895-9 (PMC12852168; doi:10.1007/s10439-025-03895-9)
Supplement: Supplementary file 12 — Supplementary file1 (PDF 507398 kb) [file 10439_2025_3895_MOESM12_ESM.pdf]

Supplementary Document

Acoustic Detection of Intracranial Cavitation Induced by Blunt Impacts in Polyacrylamide Human Head Models Across Varying Orientations

Eric J. Galindo[1] and Michaelann S. Tartis[1]

[1]Department of Chemical Engineering, New Mexico Institute of Mining and Technology, Socorro, NM 87801, USA

Supplementary Video 1
Shadowgraph imaging video of a head model filled with DI water, where the acoustic transducer is positioned to observe cavitation events at the contrecoup.

Supplementary Video 2
Shadowgraph imaging video of a head model filled with DI water, where the acoustic transducer is positioned to observe cavitation events at the approximate location of the ventricles.

Supplementary Video 3
Shadowgraph imaging video of the head model filled with DI water and rotated 90 degrees, where the acoustic transducer is positioned to image down the approximate depth of the central sulcus.

Supplementary Video 4
Shadowgraph imaging video of a head model filled with an ANGUS brain phantom, where the acoustic transducer is positioned to observe cavitation events at the contrecoup.

Supplementary Video 5
Shadowgraph imaging video of a head model filled with an ANGUS brain phantom, where the acoustic transducer is positioned to observe cavitation events at the ventricles.

Supplementary Video 6
Shadowgraph imaging video of the head model filled with an ANGUS brain phantom and rotated 90 degrees, where the acoustic transducer is positioned to image down the depth of the central sulcus.

Supplementary Video 7
Shadowgraph imaging video of a head model with asymmetrical skull geometry, filled with DI water.

Supplementary Video 8
Shadowgraph imaging video of a head model with asymmetrical skull geometry, filled with DI water and covered by a scalp layer.

Supplementary Video 9
Shadowgraph imaging video of a head model with asymmetrical skull geometry, filled with DI water and containing a dura mater mimic.

Supplementary Video 10
Shadowgraph imaging video of a head model with asymmetrical skull geometry, filled with DI water, and incorporating both a scalp layer and a dura mater mimic.

Supplementary Video 11
Shadowgraph imaging video of a head model with asymmetrical skull geometry, featuring a thin temporal bone region, filled with DI water and incorporating a scalp layer and dura mater mimic.

Contrecoup

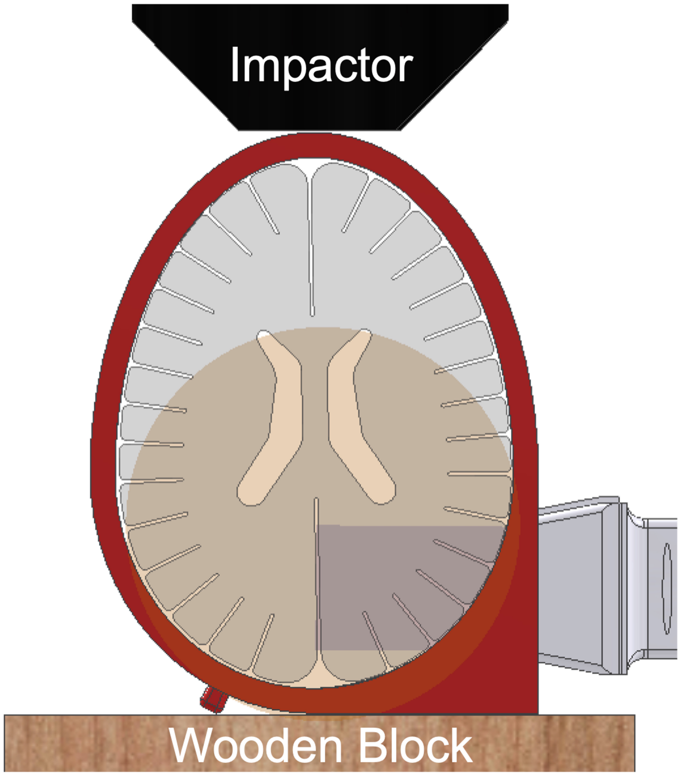

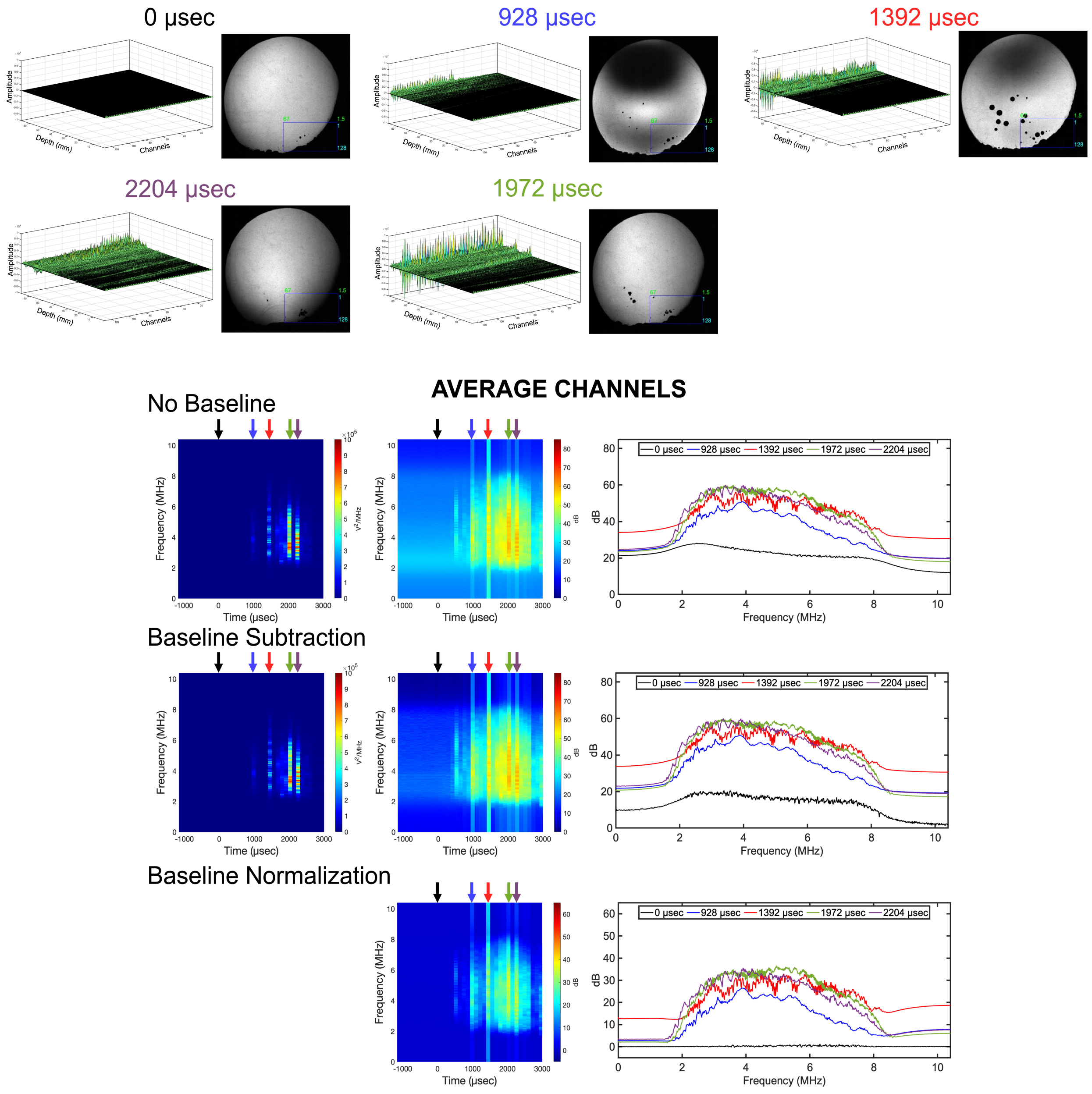

Figure 1: Comparison of averaged received channel signal processing operations on the acoustic power spectra from the first test of a head model filled with DI water, using passive cavitation detection (PCD) as the acquisition method while observing the contrecoup region. The top section presents the RF data at different time points in a 3D representation, along with the corresponding shadowgraph frames. The acoustic field of view is shown as a blue box, with channels (1–128) and axial depth (1.5–67 mm) labeled. Following this are the linear and logarithmic power spectrograms, categorized by signal processing method: no baseline correction, baseline subtraction, and baseline normalization.

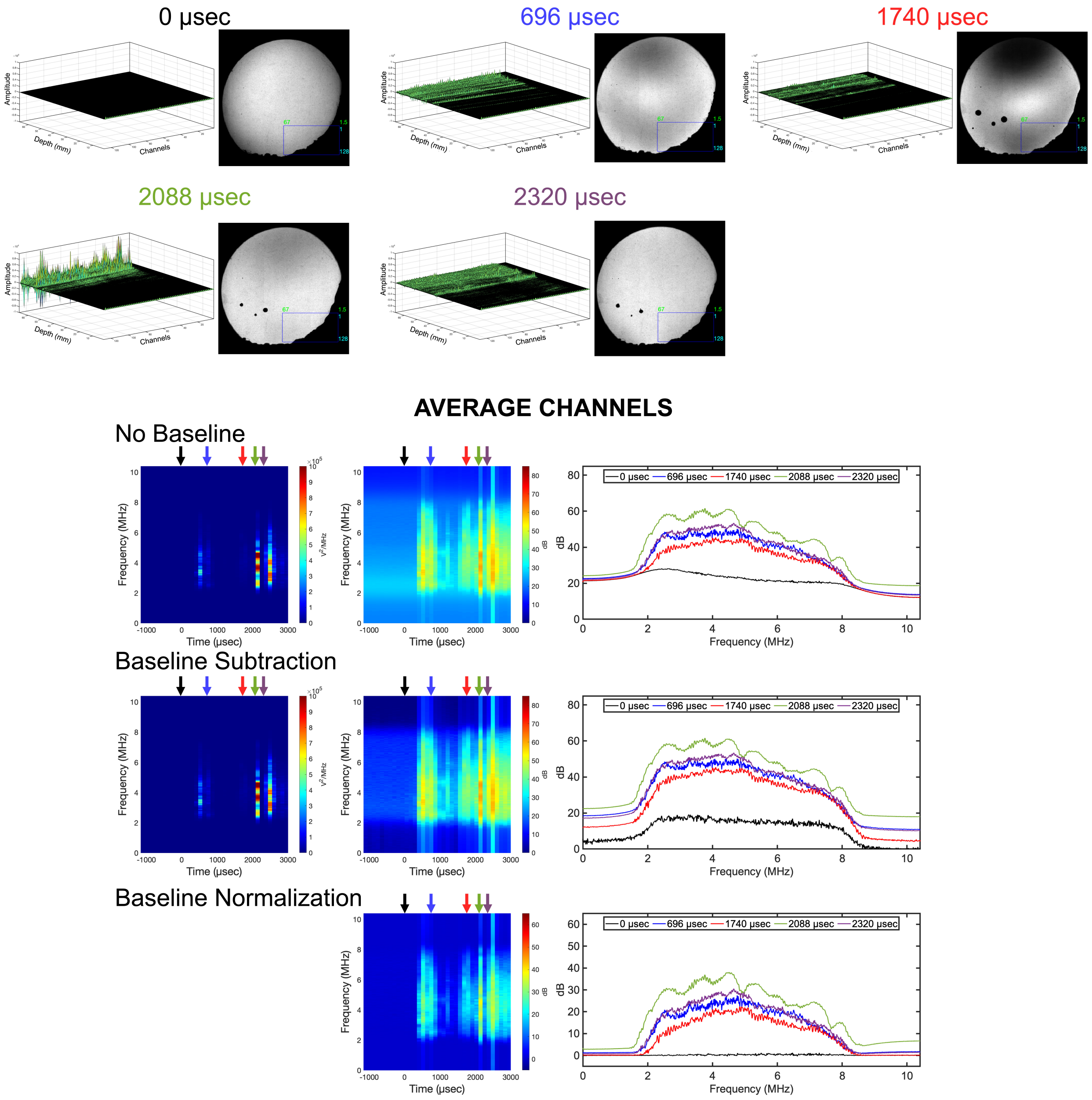

Figure 2: Comparison of averaged received channel signal processing operations on the acoustic power spectra from the second test of a head model filled with DI water, using passive cavitation detection (PCD) as the acquisition method while observing the contrecoup region. The top section presents the RF data at different time points in a 3D representation, along with the corresponding shadowgraph frames. The acoustic field of view is shown as a blue box, with channels (1–128) and axial depth (1.5–67 mm) labeled. Following this are the linear and logarithmic power spectrograms, categorized by signal processing method: no baseline correction, baseline subtraction, and baseline normalization.

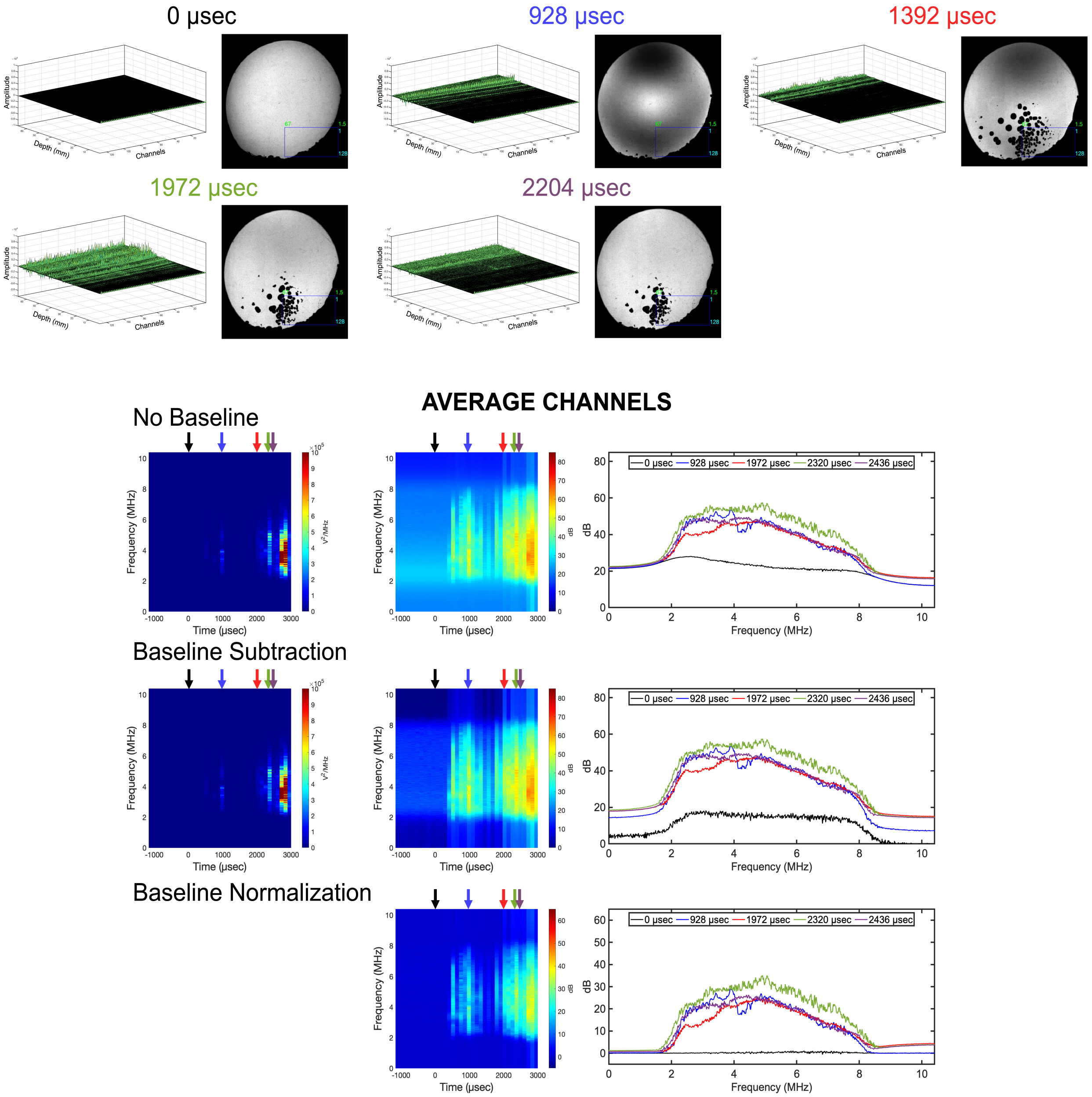

Figure 3: Comparison of averaged received channel signal processing operations on the acoustic power spectra from the third test of a head model filled with DI water, using passive cavitation detection (PCD) as the acquisition method while observing the contrecoup region. The top section presents the RF data at different time points in a 3D representation, along with the corresponding shadowgraph frames. The acoustic field of view is shown as a blue box, with channels (1–128) and axial depth (1.5–67 mm) labeled. Following this are the linear and logarithmic power spectrograms, categorized by signal processing method: no baseline correction, baseline subtraction, and baseline normalization.

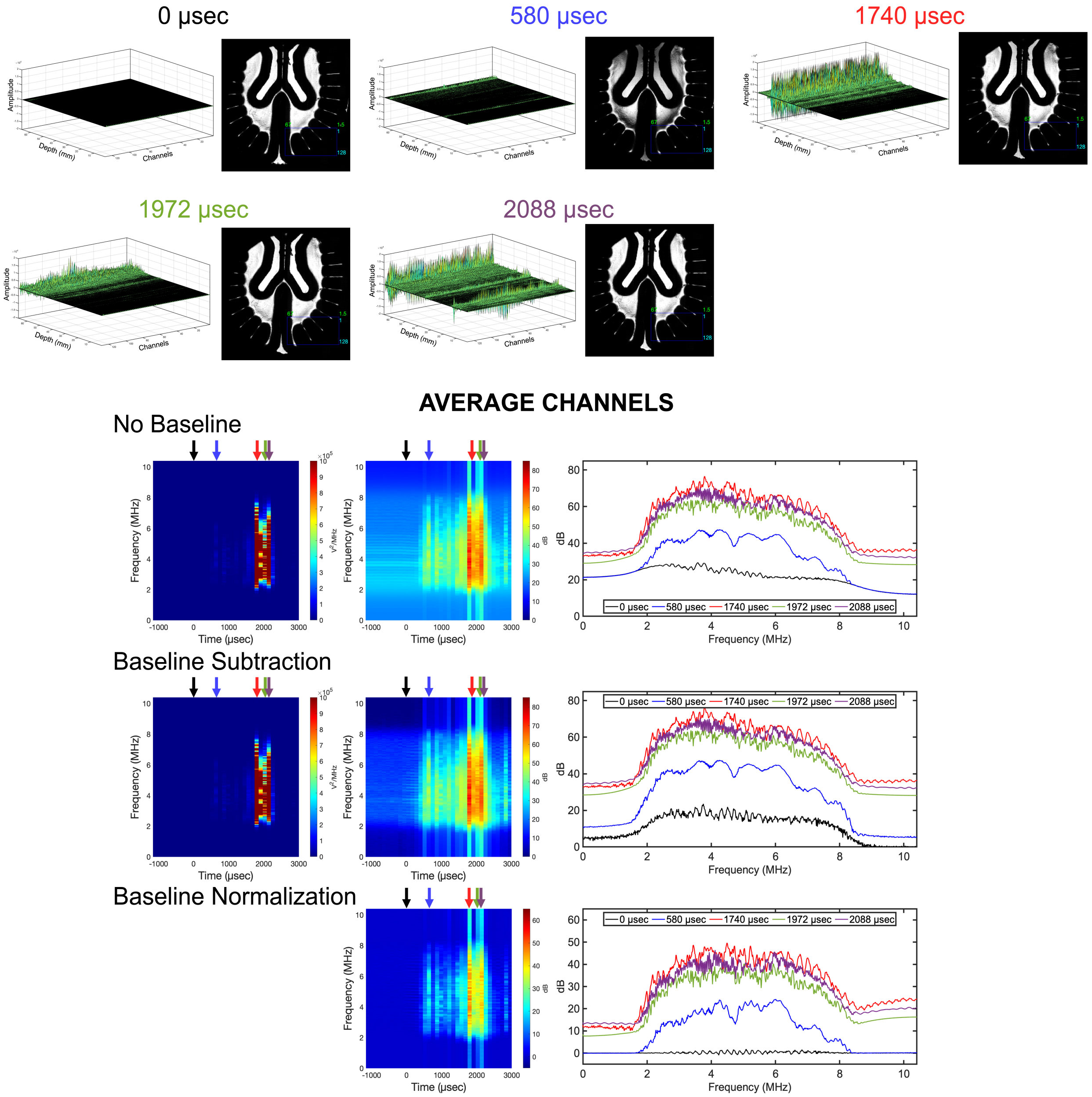

Figure 4: Comparison of averaged received channel signal processing operations on the acoustic power spectra from the first test of a head model filled with an ANGUS brain phantom, using passive cavitation detection (PCD) as the acquisition method while observing the contrecoup region. The top section presents the RF data at different time points in a 3D representation, along with the corresponding shadowgraph frames. The acoustic field of view is shown as a blue box, with channels (1–128) and axial depth (1.5–67 mm) labeled. Following this are the linear and logarithmic power spectrograms, categorized by signal processing method: no baseline correction, baseline subtraction, and baseline normalization.

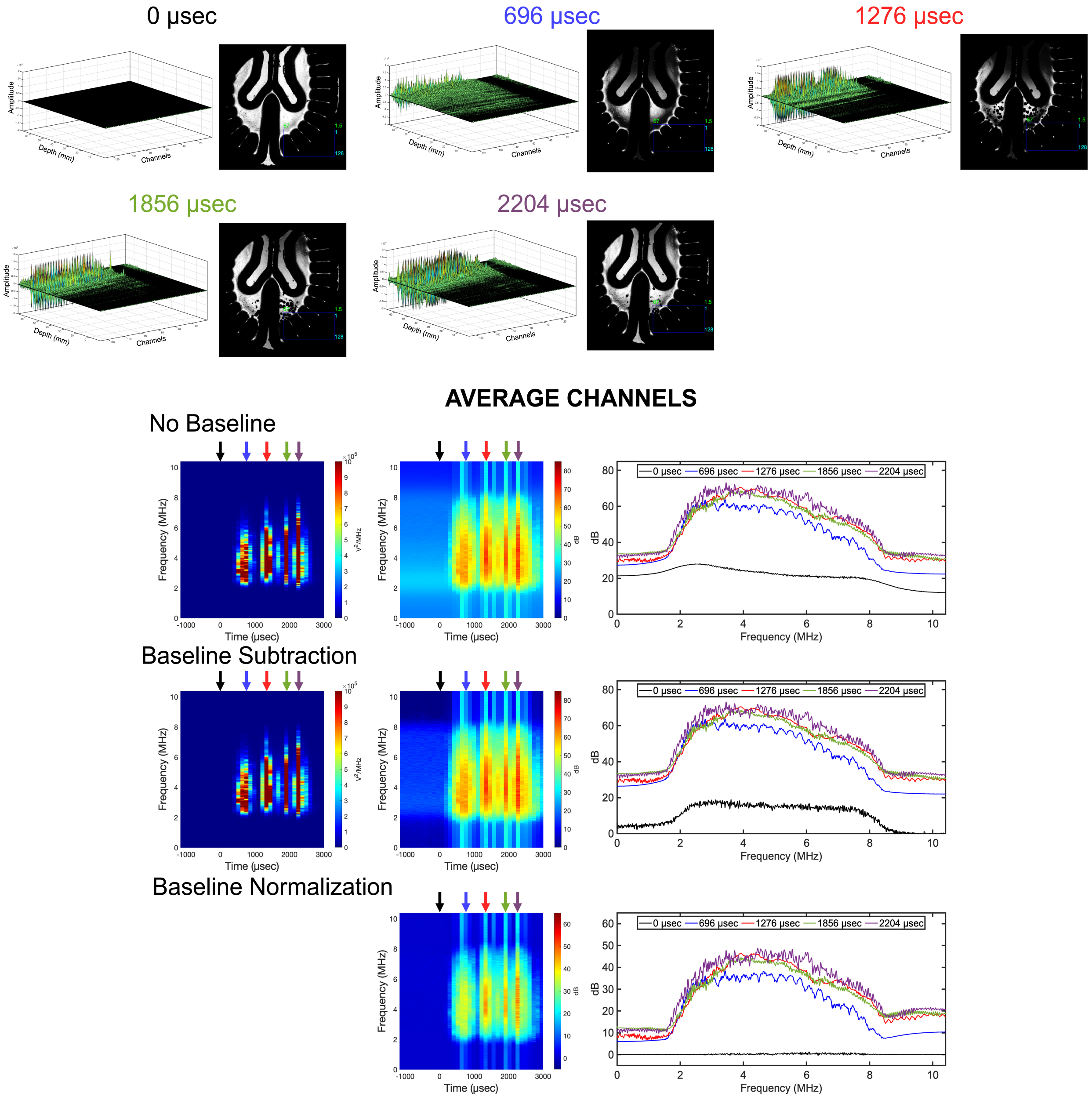

Figure 5: Comparison of averaged received channel signal processing operations on the acoustic power spectra from the second test of a head model filled with an ANGUS brain phantom, using passive cavitation detection (PCD) as the acquisition method while observing the contrecoup region. The top section presents the RF data at different time points in a 3D representation, along with the corresponding shadowgraph frames. The acoustic field of view is shown as a blue box, with channels (1–128) and axial depth (1.5–67 mm) labeled. Following this are the linear and logarithmic power spectrograms, categorized by signal processing method: no baseline correction, baseline subtraction, and baseline normalization.

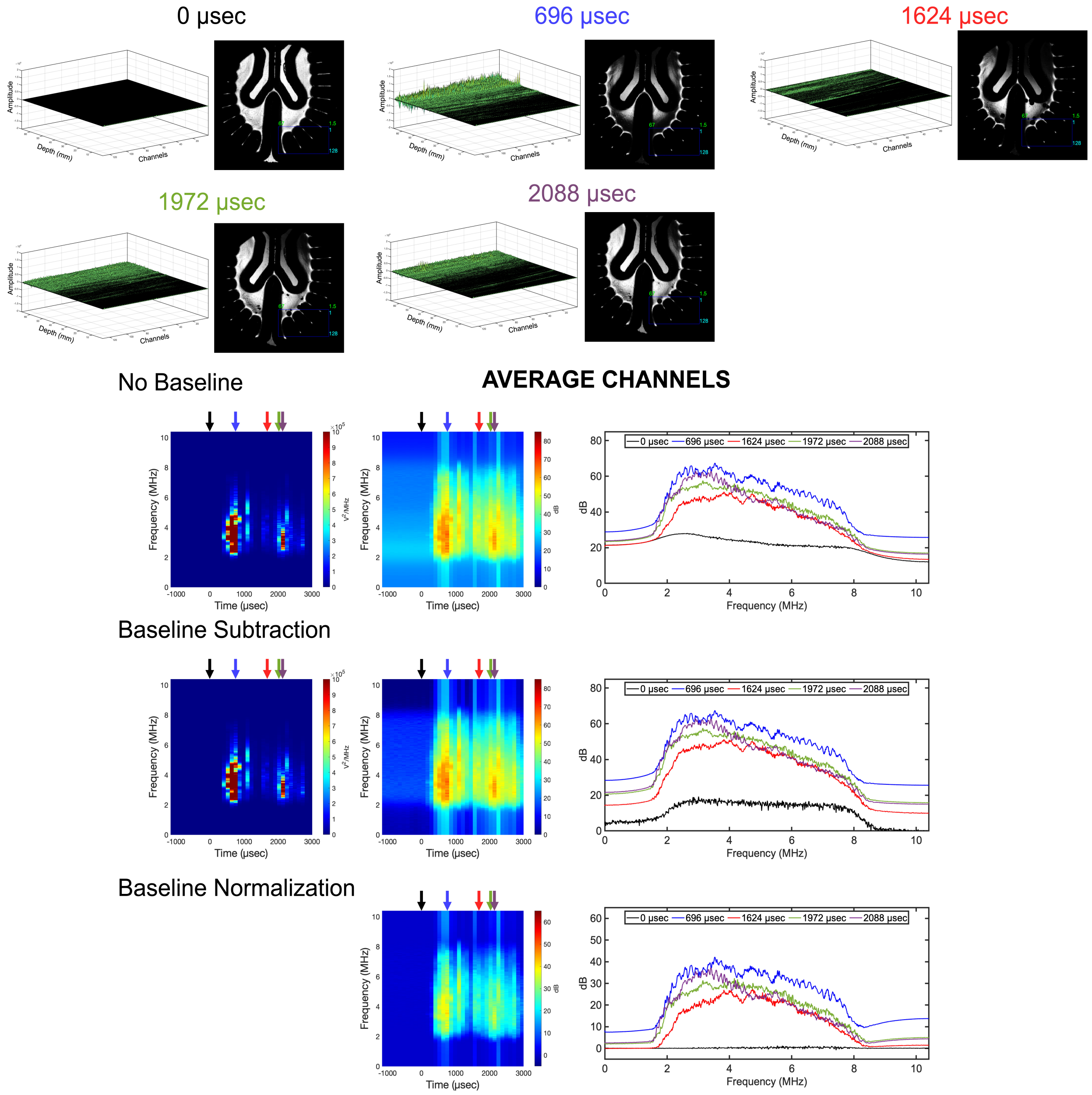

Figure 6: Comparison of averaged received channel signal processing operations on the acoustic power spectra from the third test of a head model filled with an ANGUS brain phantom, using passive cavitation detection (PCD) as the acquisition method while observing the contrecoup region. The top section presents the RF data at different time points in a 3D representation, along with the corresponding shadowgraph frames. The acoustic field of view is shown as a blue box, with channels (1–128) and axial depth (1.5–67 mm) labeled. Following this are the linear and logarithmic power spectrograms, categorized by signal processing method: no baseline correction, baseline subtraction, and baseline normalization.

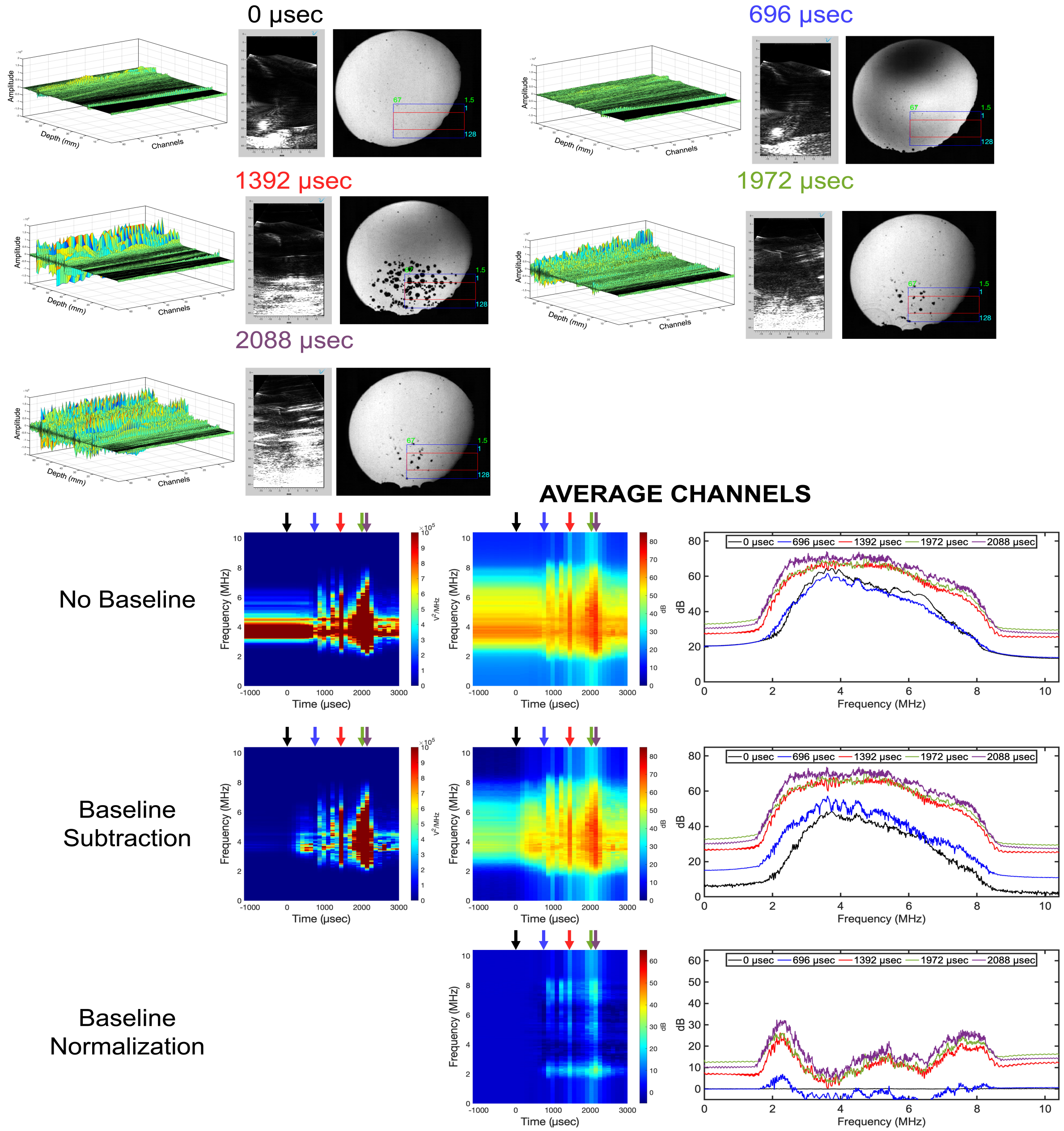

Figure 7: Comparison of averaged received channel signal processing operations applied to the acoustic power spectra from the first test of a head model filled with DI water, using plane wave imaging (PWI) as the acquisition method and observing the contrecoup region. The top section presents 3D representations of RF data at different time points, along with corresponding shadowgraph frames. The acoustic transmit field of view is indicated by a blue box (channels 1–128), and the receive channels (red box) span 32–96. Channel mapping is such that receive channels 1–64 correspond to channels 32–96, respectively. The axial depth ranges from 1.5 to 67 mm, with all channels labeled accordingly. Following this, linear and logarithmic power spectrograms are presented, categorized by signal processing method: no baseline correction, baseline subtraction, and baseline normalization.

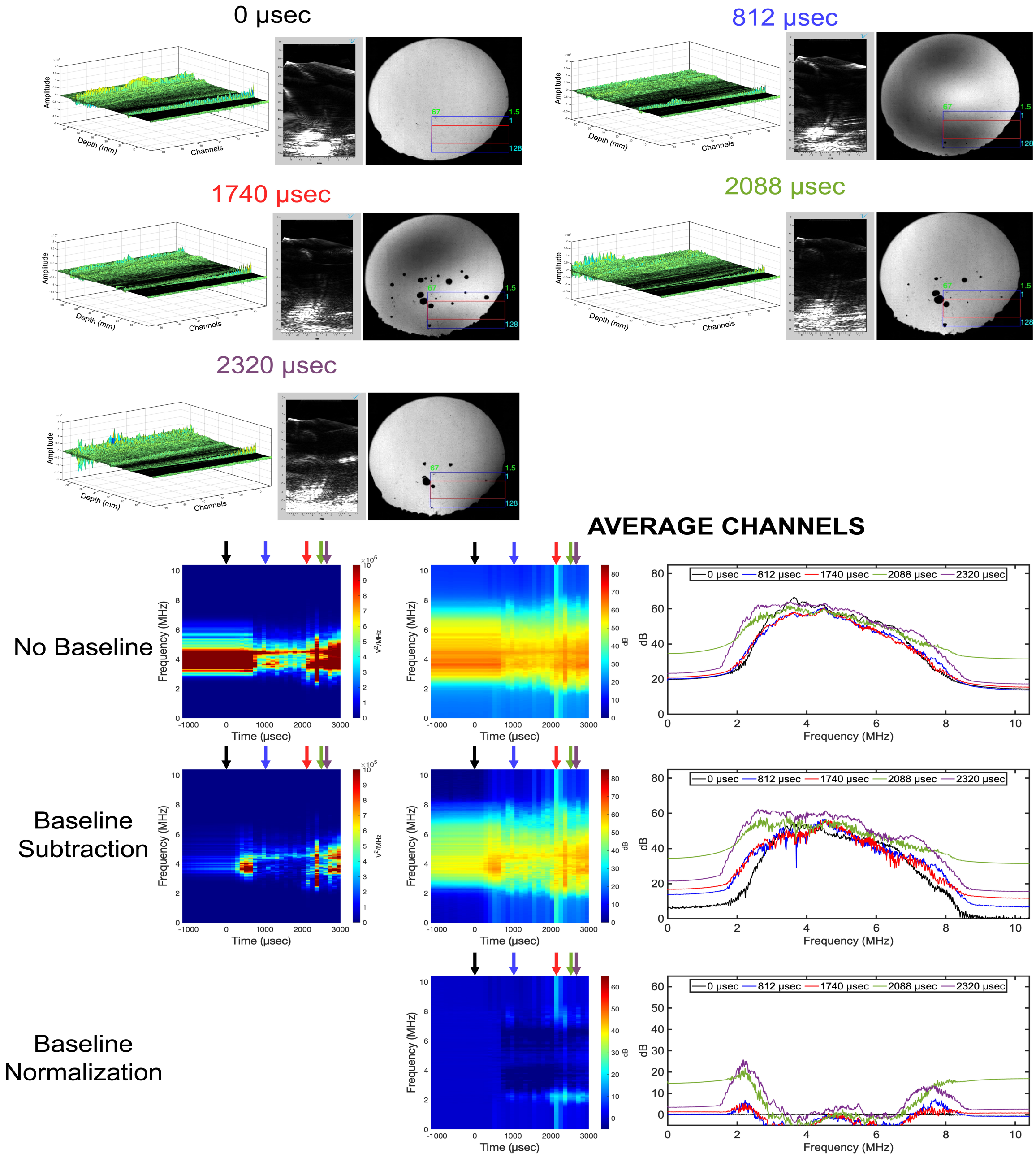

Figure 8: Comparison of averaged received channel signal processing operations applied to the acoustic power spectra from the second test of a head model filled with DI water, using plane wave imaging (PWI) as the acquisition method and observing the contrecoup region. The top section presents 3D representations of RF data at different time points, along with corresponding shadowgraph frames. The acoustic transmit field of view is indicated by a blue box (channels 1–128), and the receive channels (red box) span 32–96. Channel mapping is such that receive channels 1–64 correspond to channels 32–96, respectively. The axial depth ranges from 1.5 to 67 mm, with all channels labeled accordingly. Following this, linear and logarithmic power spectrograms are presented, categorized by signal processing method: no baseline correction, baseline subtraction, and baseline normalization.

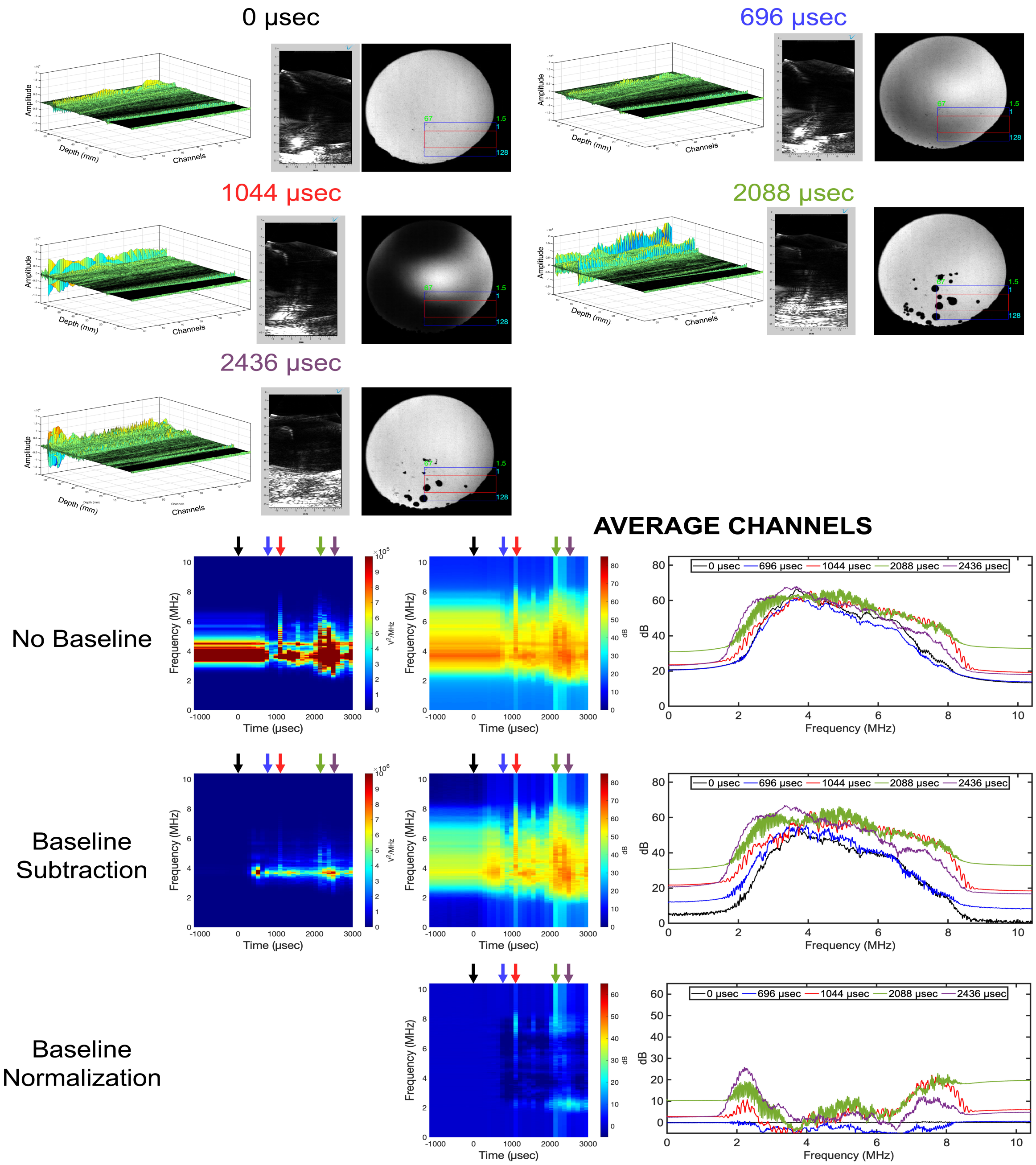

Figure 9: Comparison of averaged received channel signal processing operations applied to the acoustic power spectra from the third test of a head model filled with DI water, using plane wave imaging (PWI) as the acquisition method and observing the contrecoup region. The top section presents 3D representations of RF data at different time points, along with corresponding shadowgraph frames. The acoustic transmit field of view is indicated by a blue box (channels 1–128), and the receive channels (red box) span 32–96. Channel mapping is such that receive channels 1–64 correspond to channels 32–96, respectively. The axial depth ranges from 1.5 to 67 mm, with all channels labeled accordingly. Following this, linear and logarithmic power spectrograms are presented, categorized by signal processing method: no baseline correction, baseline subtraction, and baseline normalization.

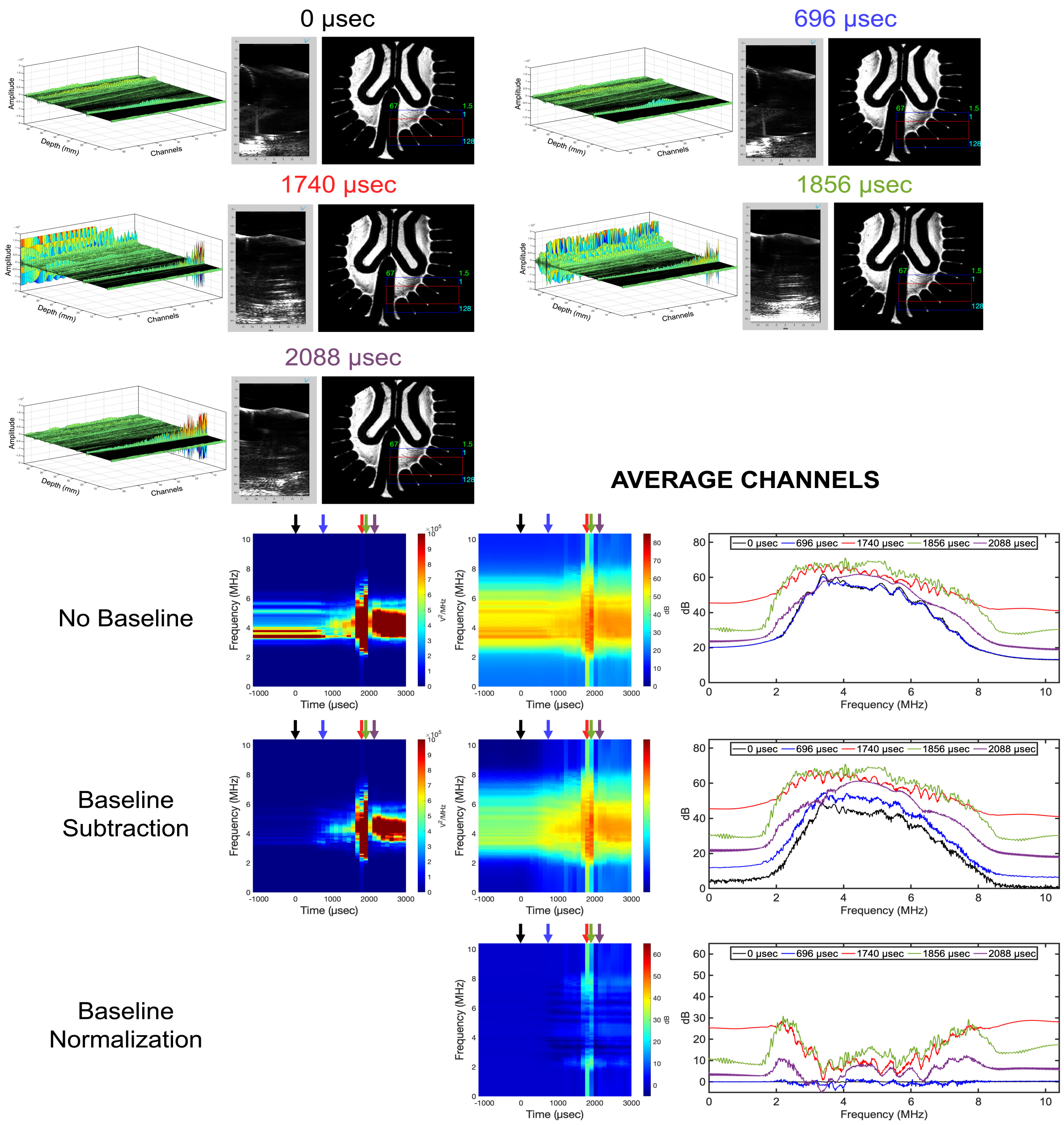

Figure 10: Comparison of averaged received channel signal processing operations applied to the acoustic power spectra from the first test of a head model filled with an ANGUS brain phantom, using plane wave imaging (PWI) as the acquisition method and observing the contrecoup region. The top section presents 3D representations of RF data at different time points, along with corresponding shadowgraph frames. The acoustic transmit field of view is indicated by a blue box (channels 1–128), and the receive channels (red box) span 32–96. Channel mapping is such that receive channels 1–64 correspond to channels 32–96, respectively. The axial depth ranges from 1.5 to 67 mm, with all channels labeled accordingly. Following this, linear and logarithmic power spectrograms are presented, categorized by signal processing method: no baseline correction, baseline subtraction, and baseline normalization.

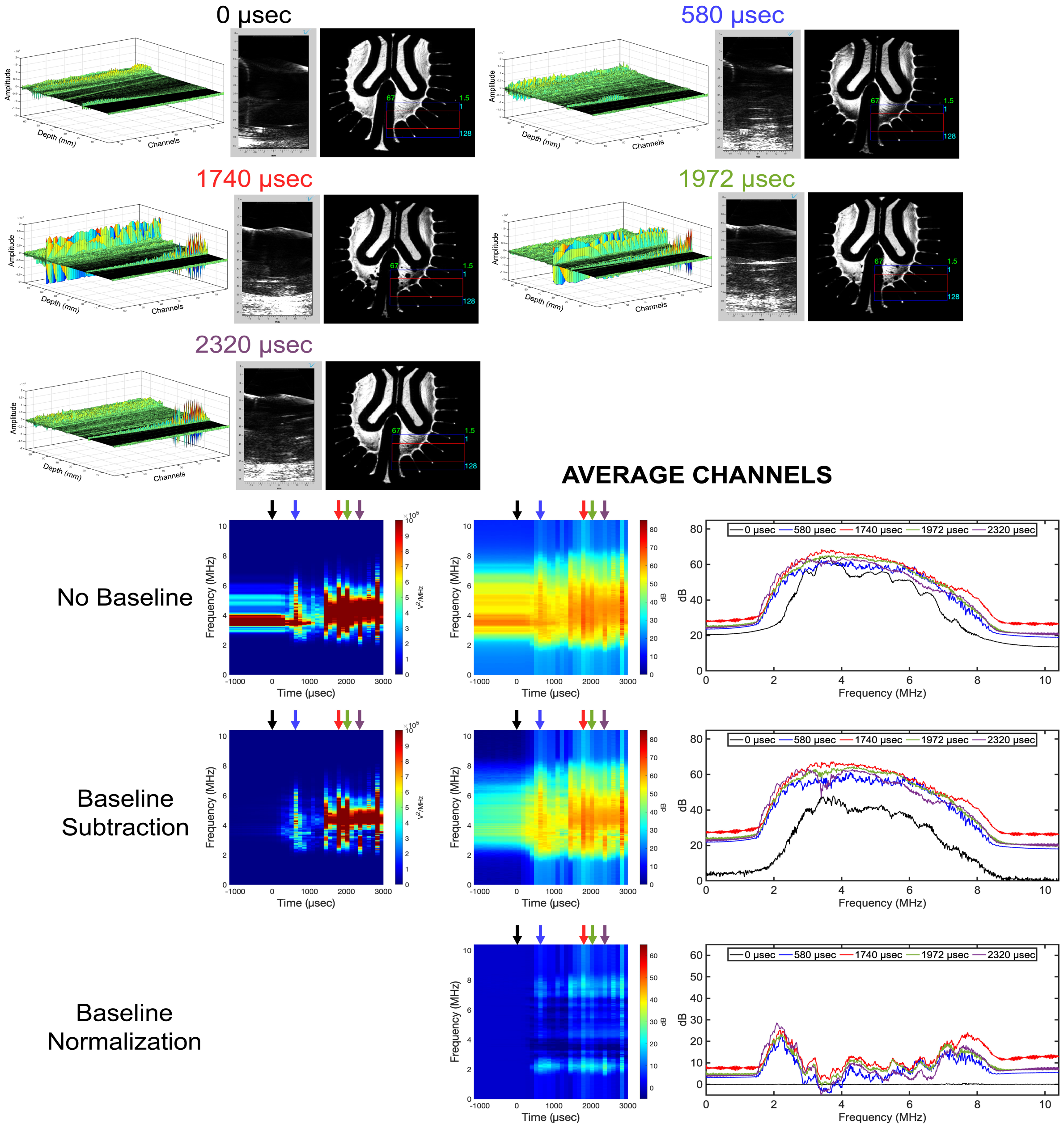

Figure 11: Comparison of averaged received channel signal processing operations applied to the acoustic power spectra from the second test of a head model filled with an ANGUS brain phantom, using plane wave imaging (PWI) as the acquisition method and observing the contrecoup region. The top section presents 3D representations of RF data at different time points, along with corresponding shadowgraph frames. The acoustic transmit field of view is indicated by a blue box (channels 1–128), and the receive channels (red box) span 32–96. Channel mapping is such that receive channels 1–64 correspond to channels 32–96, respectively. The axial depth ranges from 1.5 to 67 mm, with all channels labeled accordingly. Following this, linear and logarithmic power spectrograms are presented, categorized by signal processing method: no baseline correction, baseline subtraction, and baseline normalization.

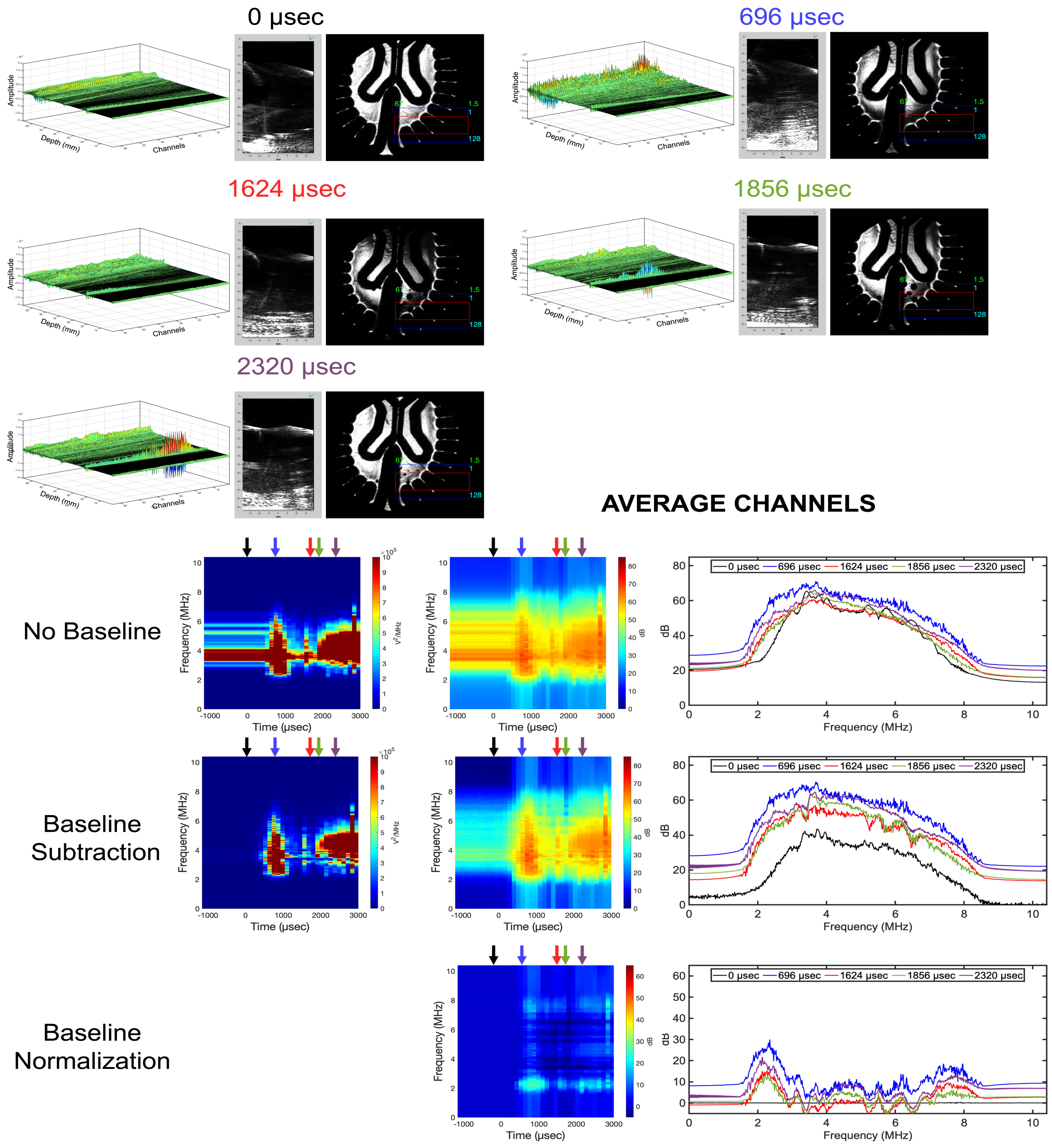

Figure 12: Comparison of averaged received channel signal processing operations applied to the acoustic power spectra from the third test of a head model filled with an ANGUS brain phantom, using plane wave imaging (PWI) as the acquisition method and observing the contrecoup region. The top section presents 3D representations of RF data at different time points, along with corresponding shadowgraph frames. The acoustic transmit field of view is indicated by a blue box (channels 1–128), and the receive channels (red box) span 32–96. Channel mapping is such that receive channels 1–64 correspond to channels 32–96, respectively. The axial depth ranges from 1.5 to 67 mm, with all channels labeled accordingly. Following this, linear and logarithmic power spectrograms are presented, categorized by signal processing method: no baseline correction, baseline subtraction, and baseline normalization.

Ventricles

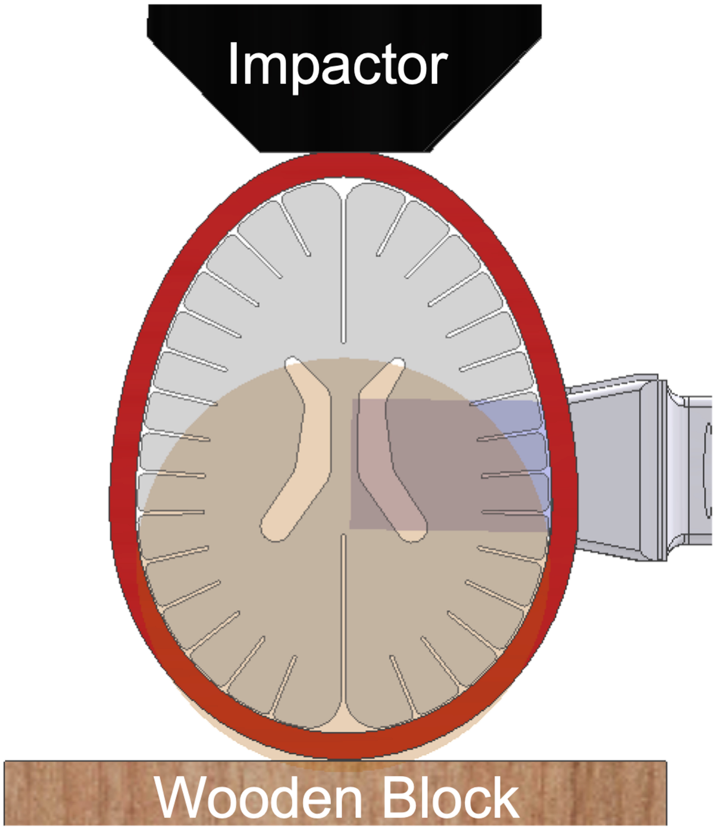

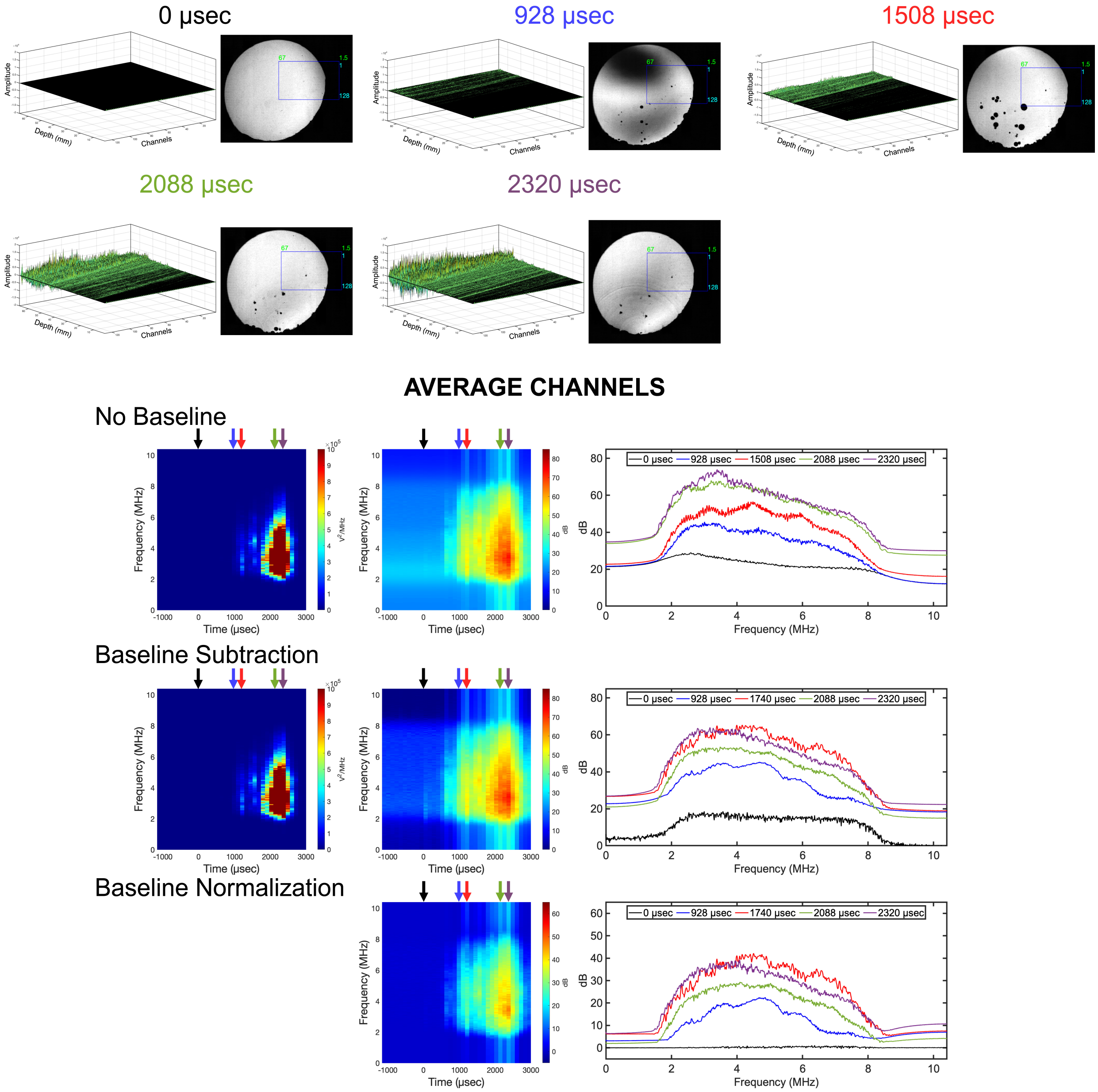

Figure 13: Comparison of averaged received channel signal processing operations on the acoustic power spectra from the first test of a head model filled with DI water, using passive cavitation detection (PCD) as the acquisition method while observing the ventricular region. The top section presents the RF data at different time points in a 3D representation, along with the corresponding shadowgraph frames. The acoustic field of view is shown as a blue box, with channels (1–128) and axial depth (1.5–67 mm) labeled. Following this are the linear and logarithmic power spectrograms, categorized by signal processing method: no baseline correction, baseline subtraction, and baseline normalization.

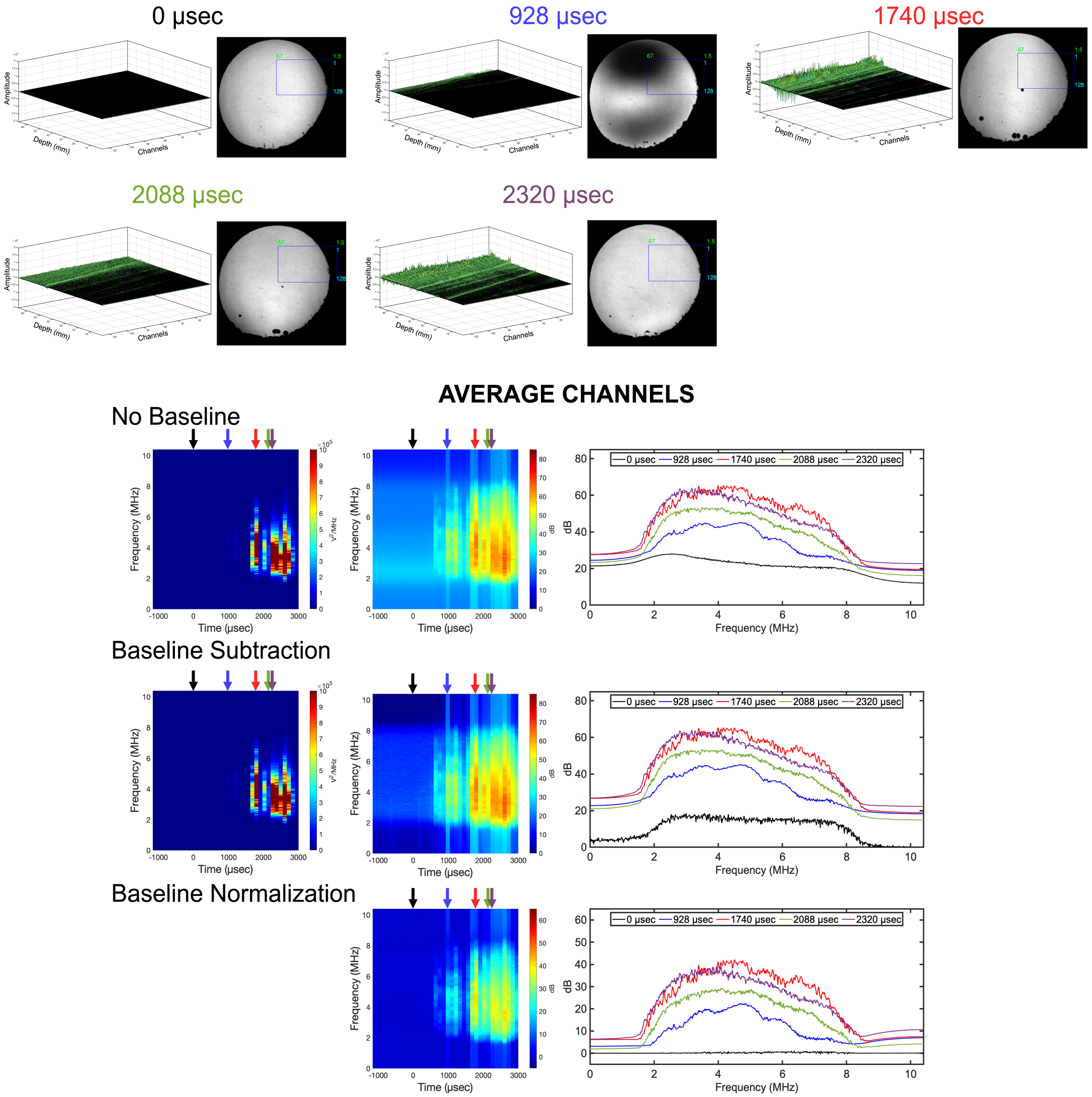

Figure 14: Comparison of averaged received channel signal processing operations on the acoustic power spectra from the second test of a head model filled with DI water, using passive cavitation detection (PCD) as the acquisition method while observing the ventricular region. The top section presents the RF data at different time points in a 3D representation, along with the corresponding shadowgraph frames. The acoustic field of view is shown as a blue box, with channels (1–128) and axial depth (1.5–67 mm) labeled. Following this are the linear and logarithmic power spectrograms, categorized by signal processing method: no baseline correction, baseline subtraction, and baseline normalization.

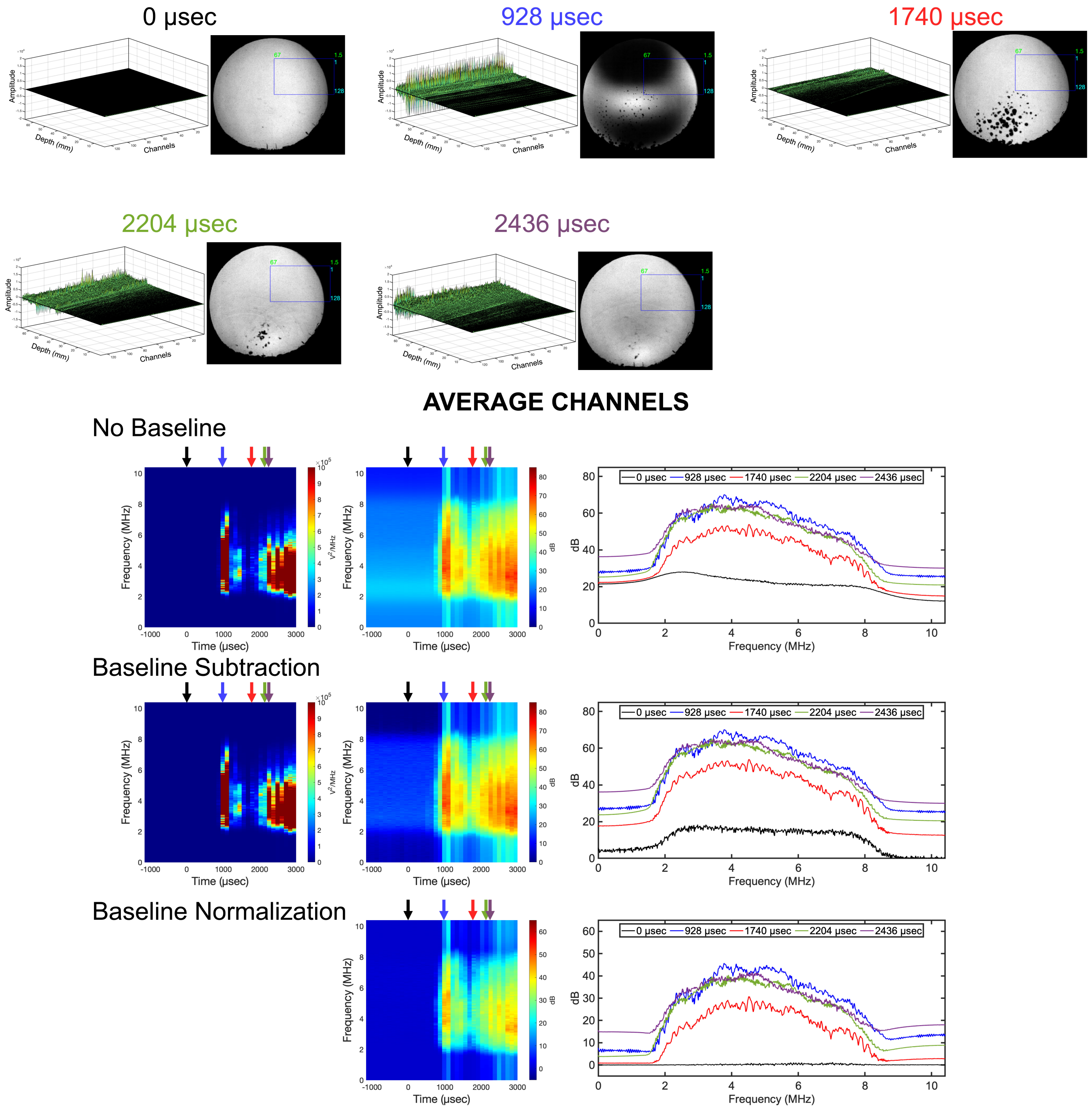

Figure 15: Comparison of averaged received channel signal processing operations on the acoustic power spectra from the third test of a head model filled with DI water, using passive cavitation detection (PCD) as the acquisition method while observing the ventricular region. The top section presents the RF data at different time points in a 3D representation, along with the corresponding shadowgraph frames. The acoustic field of view is shown as a blue box, with channels (1–128) and axial depth (1.5–67 mm) labeled. Following this are the linear and logarithmic power spectrograms, categorized by signal processing method: no baseline correction, baseline subtraction, and baseline normalization.

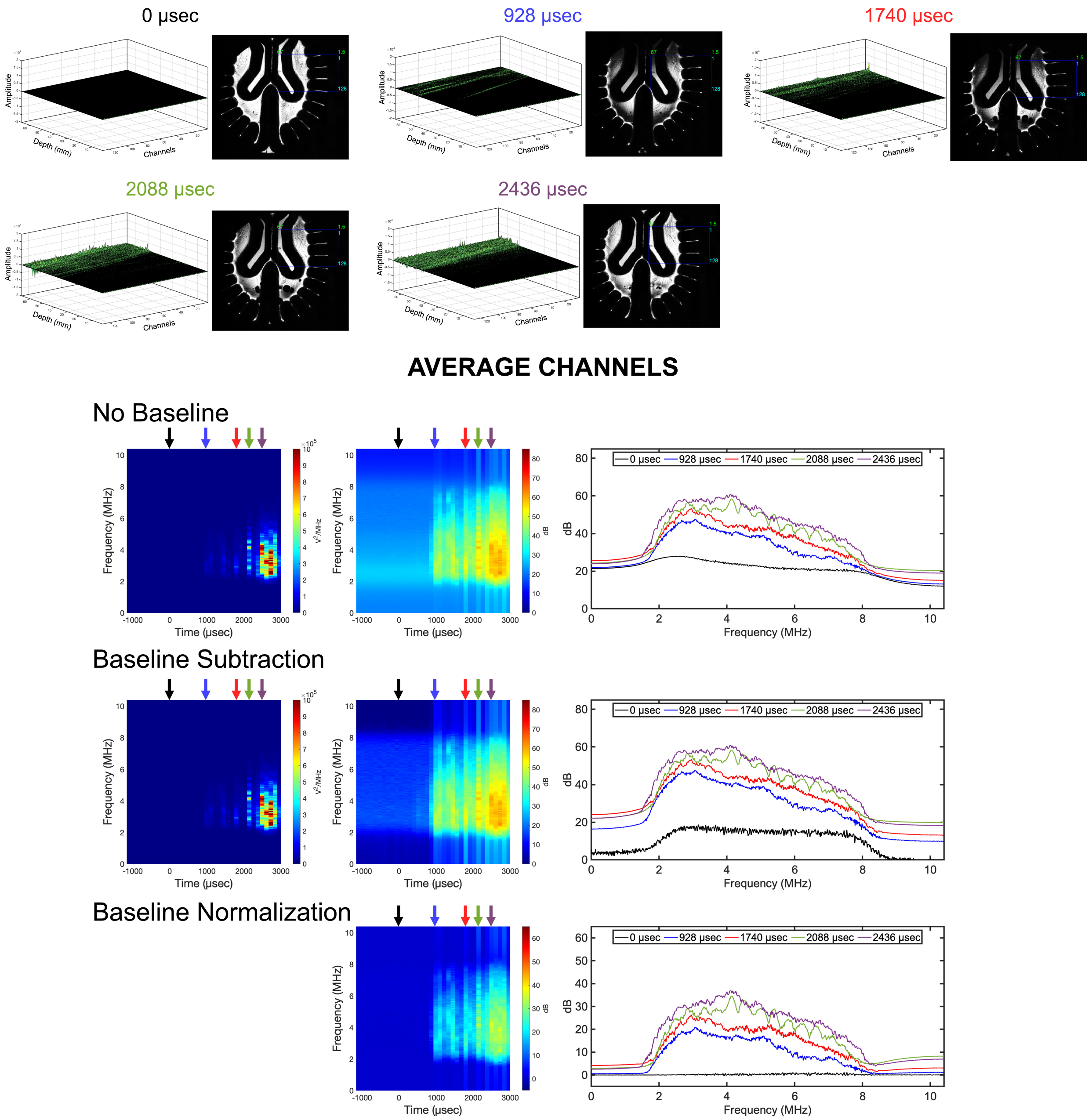

Figure 16: Comparison of averaged received channel signal processing operations on the acoustic power spectra from the first test of a head model filled with an ANGUS brain phantom, using passive cavitation detection (PCD) as the acquisition method while observing the ventricular region. The top section presents the RF data at different time points in a 3D representation, along with the corresponding shadowgraph frames. The acoustic field of view is shown as a blue box, with channels (1–128) and axial depth (1.5–67 mm) labeled. Following this are the linear and logarithmic power spectrograms, categorized by signal processing method: no baseline correction, baseline subtraction, and baseline normalization.

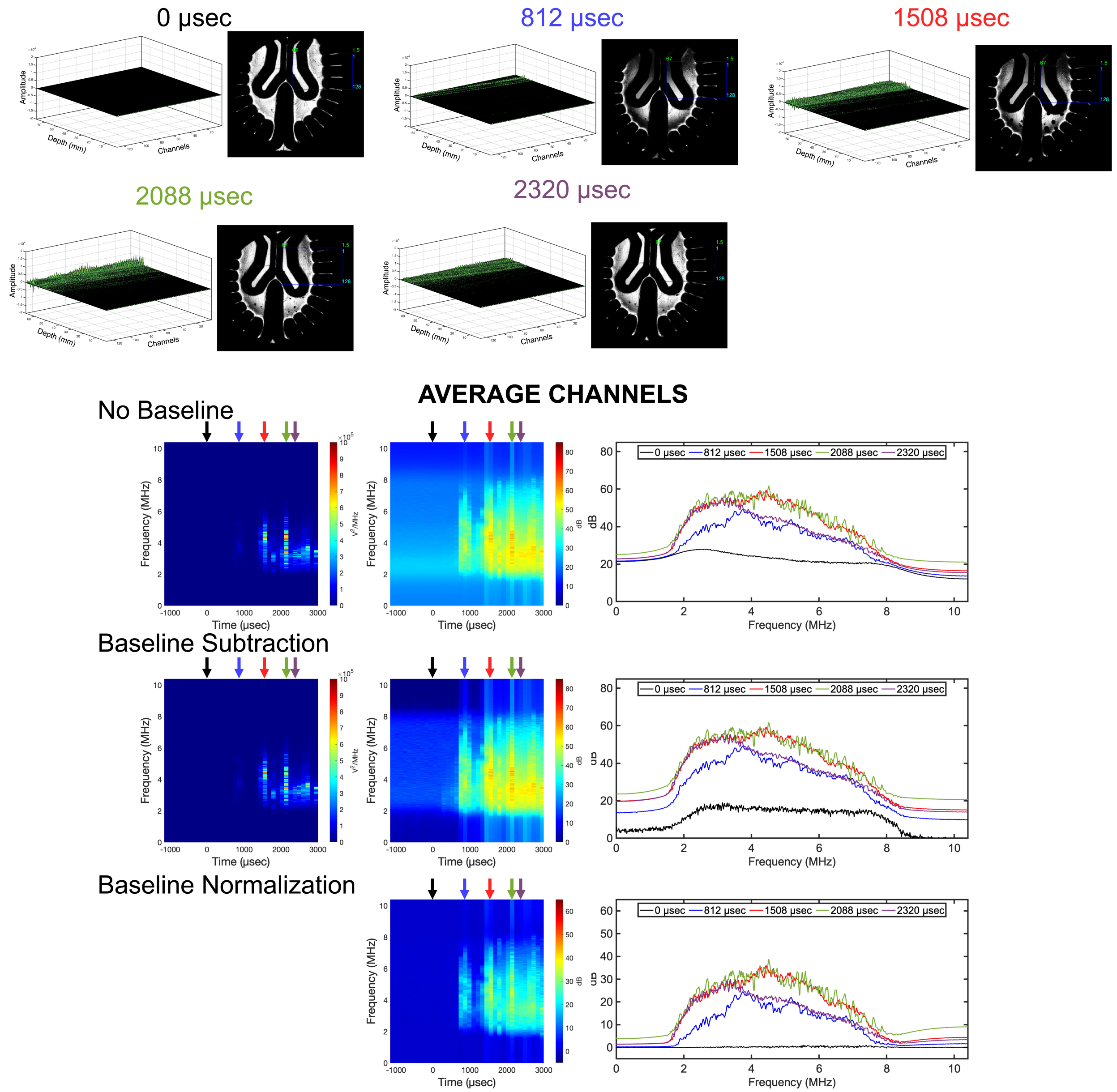

Figure 17: Comparison of averaged received channel signal processing operations on the acoustic power spectra from the second test of a head model filled with an ANGUS brain phantom, using passive cavitation detection (PCD) as the acquisition method while observing the ventricular region. The top section presents the RF data at different time points in a 3D representation, along with the corresponding shadowgraph frames. The acoustic field of view is shown as a blue box, with channels (1–128) and axial depth (1.5–67 mm) labeled. Following this are the linear and logarithmic power spectrograms, categorized by signal processing method: no baseline correction, baseline subtraction, and baseline normalization.

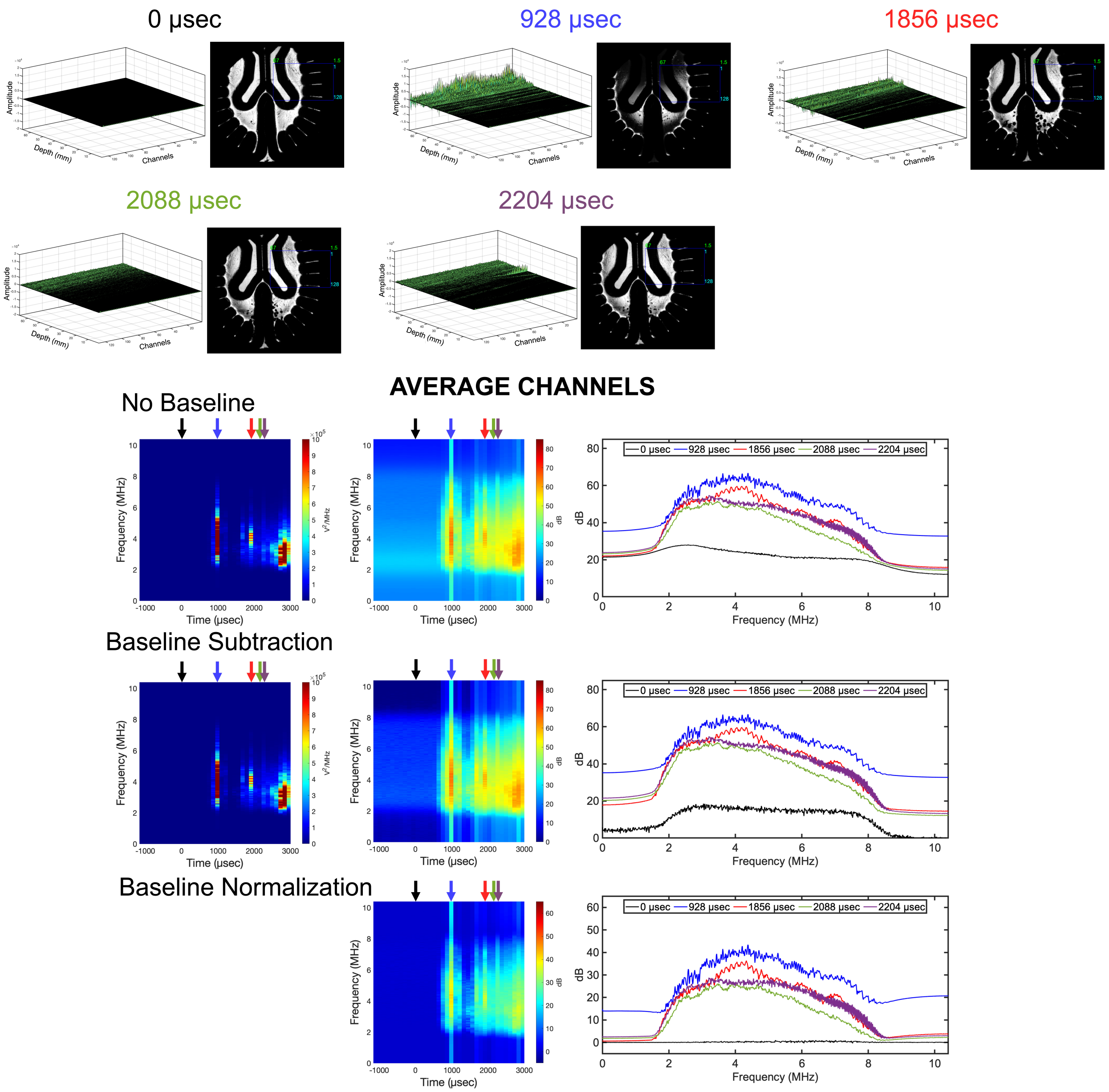

Figure 18: Comparison of averaged received channel signal processing operations on the acoustic power spectra from the third test of a head model filled with an ANGUS brain phantom, using passive cavitation detection (PCD) as the acquisition method while observing the ventricular region. The top section presents the RF data at different time points in a 3D representation, along with the corresponding shadowgraph frames. The acoustic field of view is shown as a blue box, with channels (1–128) and axial depth (1.5–67 mm) labeled. Following this are the linear and logarithmic power spectrograms, categorized by signal processing method: no baseline correction, baseline subtraction, and baseline normalization.

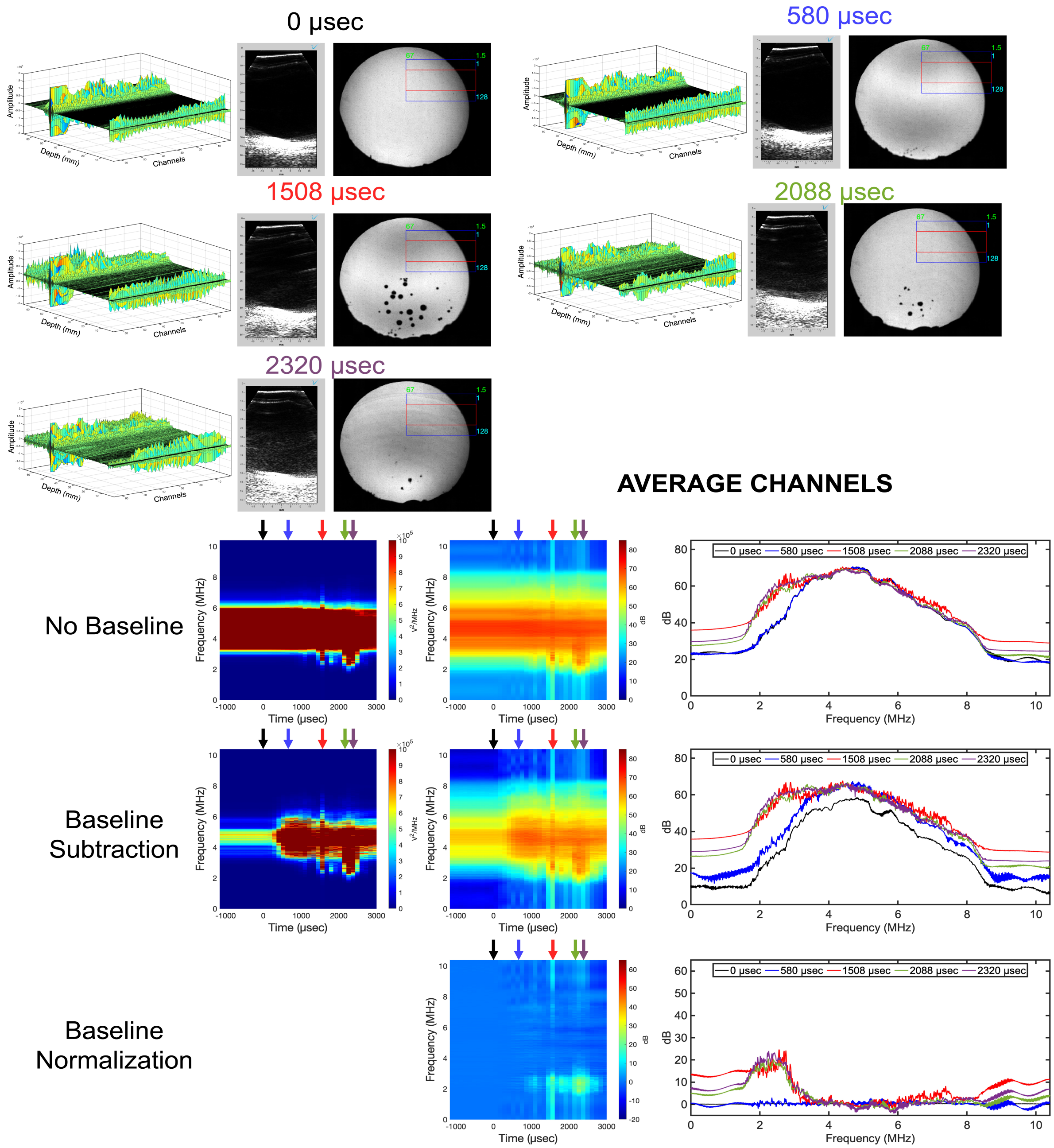

Figure 19: Comparison of averaged received channel signal processing operations applied to the acoustic power spectra from the first test of a head model filled with DI water, using plane wave imaging (PWI) as the acquisition method and observing the ventricular region. The top section presents 3D representations of RF data at different time points, along with corresponding shadowgraph frames. The acoustic transmit field of view is indicated by a blue box (channels 1–128), and the receive channels (red box) span 32–96. Channel mapping is such that receive channels 1–64 correspond to channels 32–96, respectively. The axial depth ranges from 1.5 to 67 mm, with all channels labeled accordingly. Following this, linear and logarithmic power spectrograms are presented, categorized by signal processing method: no baseline correction, baseline subtraction, and baseline normalization.

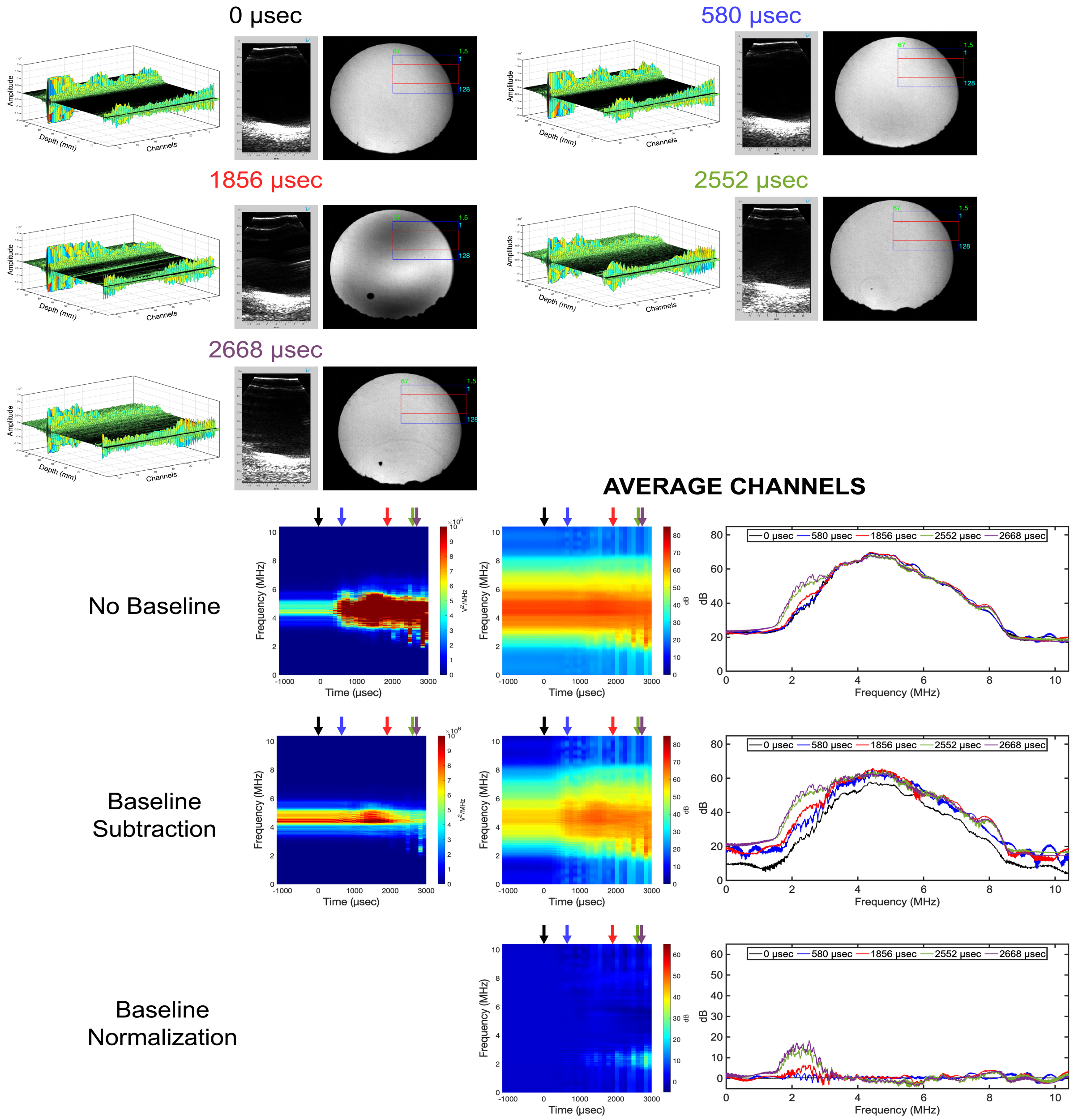

Figure 20: Comparison of averaged received channel signal processing operations applied to the acoustic power spectra from the second test of a head model filled with DI water, using plane wave imaging (PWI) as the acquisition method and observing the ventricular region. The top section presents 3D representations of RF data at different time points, along with corresponding shadowgraph frames. The acoustic transmit field of view is indicated by a blue box (channels 1–128), and the receive channels (red box) span 32–96. Channel mapping is such that receive channels 1–64 correspond to channels 32–96, respectively. The axial depth ranges from 1.5 to 67 mm, with all channels labeled accordingly. Following this, linear and logarithmic power spectrograms are presented, categorized by signal processing method: no baseline correction, baseline subtraction, and baseline normalization.

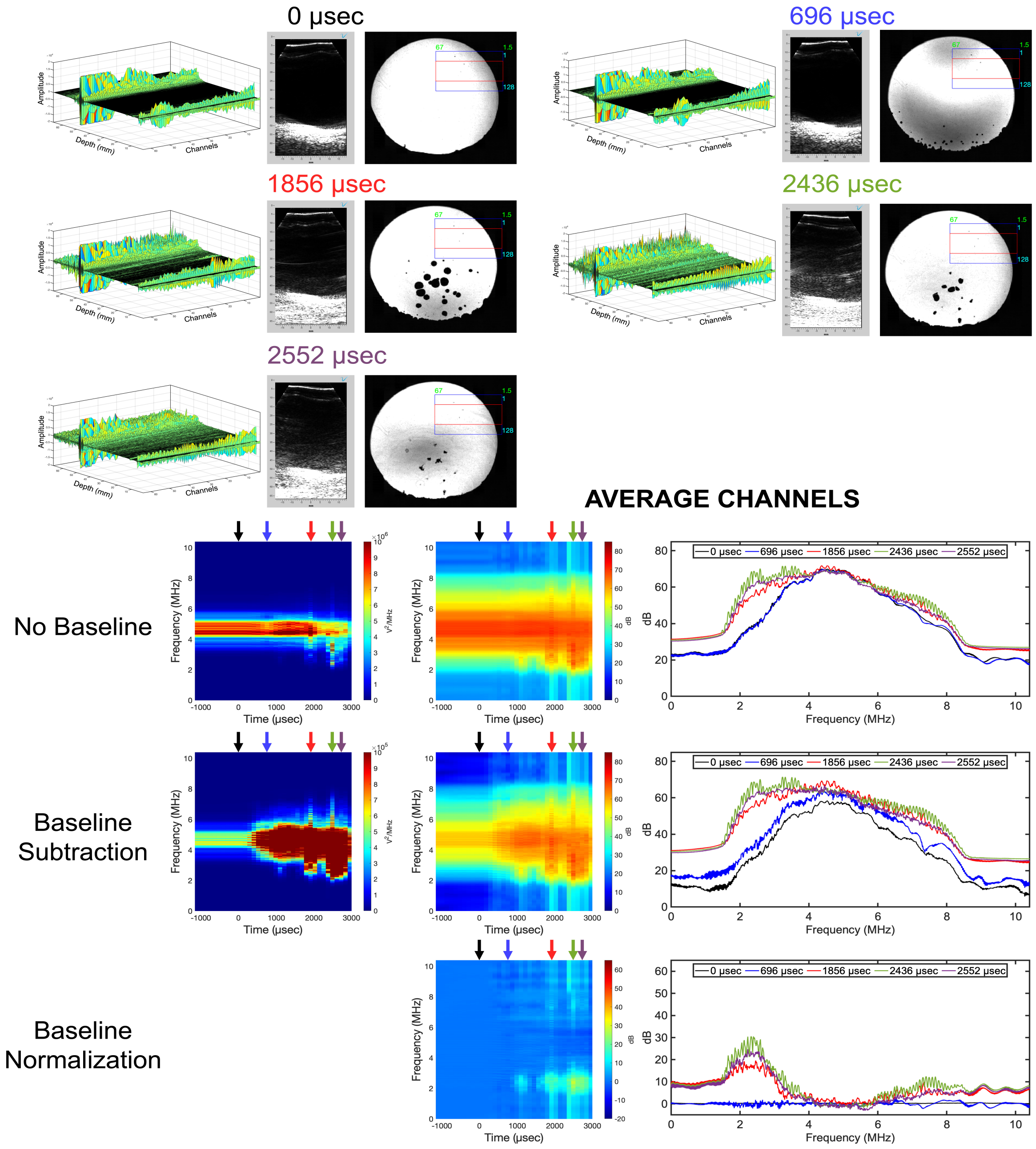

Figure 21: Comparison of averaged received channel signal processing operations applied to the acoustic power spectra from the third test of a head model filled with DI water, using plane wave imaging (PWI) as the acquisition method and observing the ventricular region. The top section presents 3D representations of RF data at different time points, along with corresponding shadowgraph frames. The acoustic transmit field of view is indicated by a blue box (channels 1–128), and the receive channels (red box) span 32–96. Channel mapping is such that receive channels 1–64 correspond to channels 32–96, respectively. The axial depth ranges from 1.5 to 67 mm, with all channels labeled accordingly. Following this, linear and logarithmic power spectrograms are presented, categorized by signal processing method: no baseline correction, baseline subtraction, and baseline normalization.

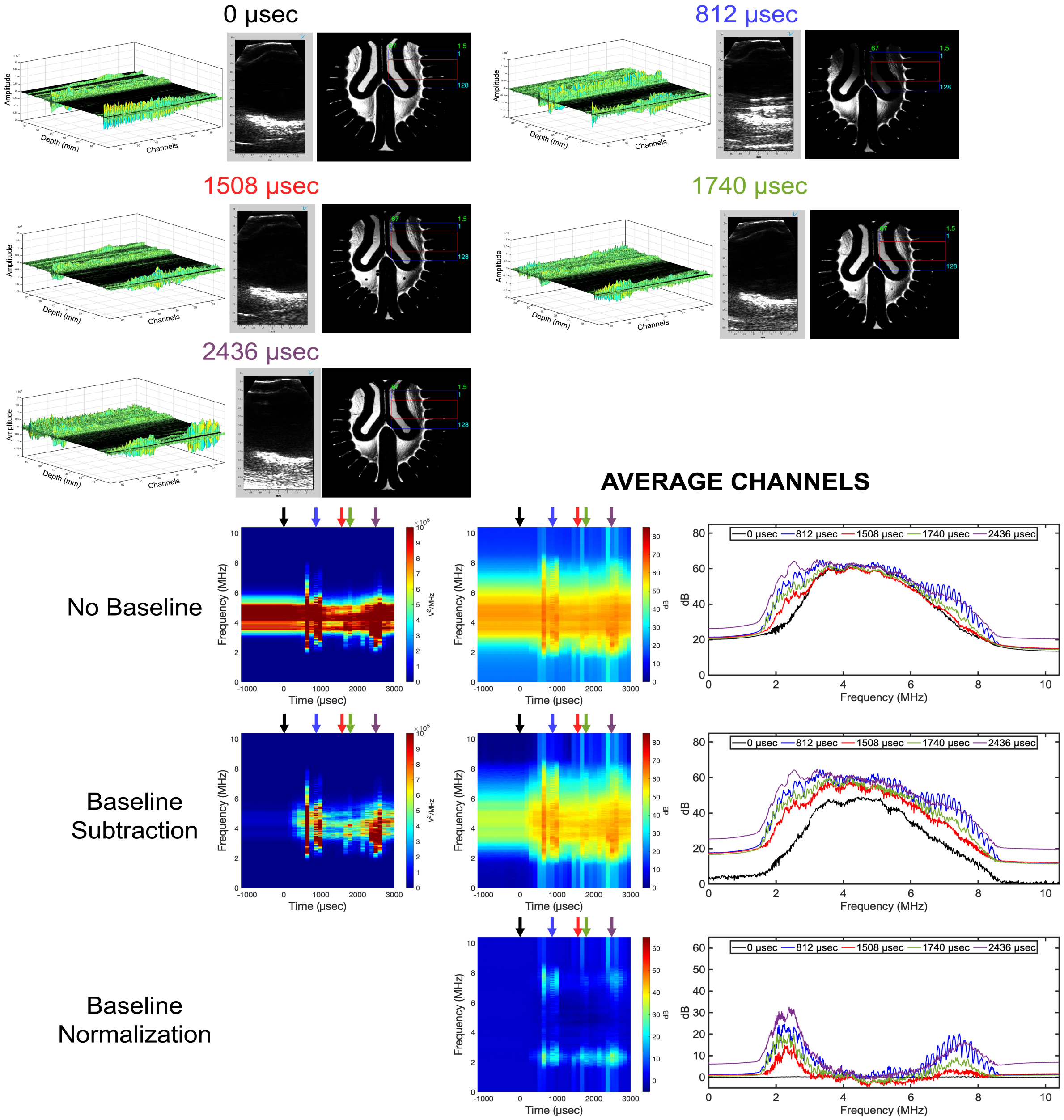

Figure 22: Comparison of averaged received channel signal processing operations applied to the acoustic power spectra from the first test of a head model filled with an ANGUS brain phantom, using plane wave imaging (PWI) as the acquisition method and observing the ventricular region. The top section presents 3D representations of RF data at different time points, along with corresponding shadowgraph frames. The acoustic transmit field of view is indicated by a blue box (channels 1–128), and the receive channels (red box) span 32–96. Channel mapping is such that receive channels 1–64 correspond to channels 32–96, respectively. The axial depth ranges from 1.5 to 67 mm, with all channels labeled accordingly. Following this, linear and logarithmic power spectrograms are presented, categorized by signal processing method: no baseline correction, baseline subtraction, and baseline normalization.

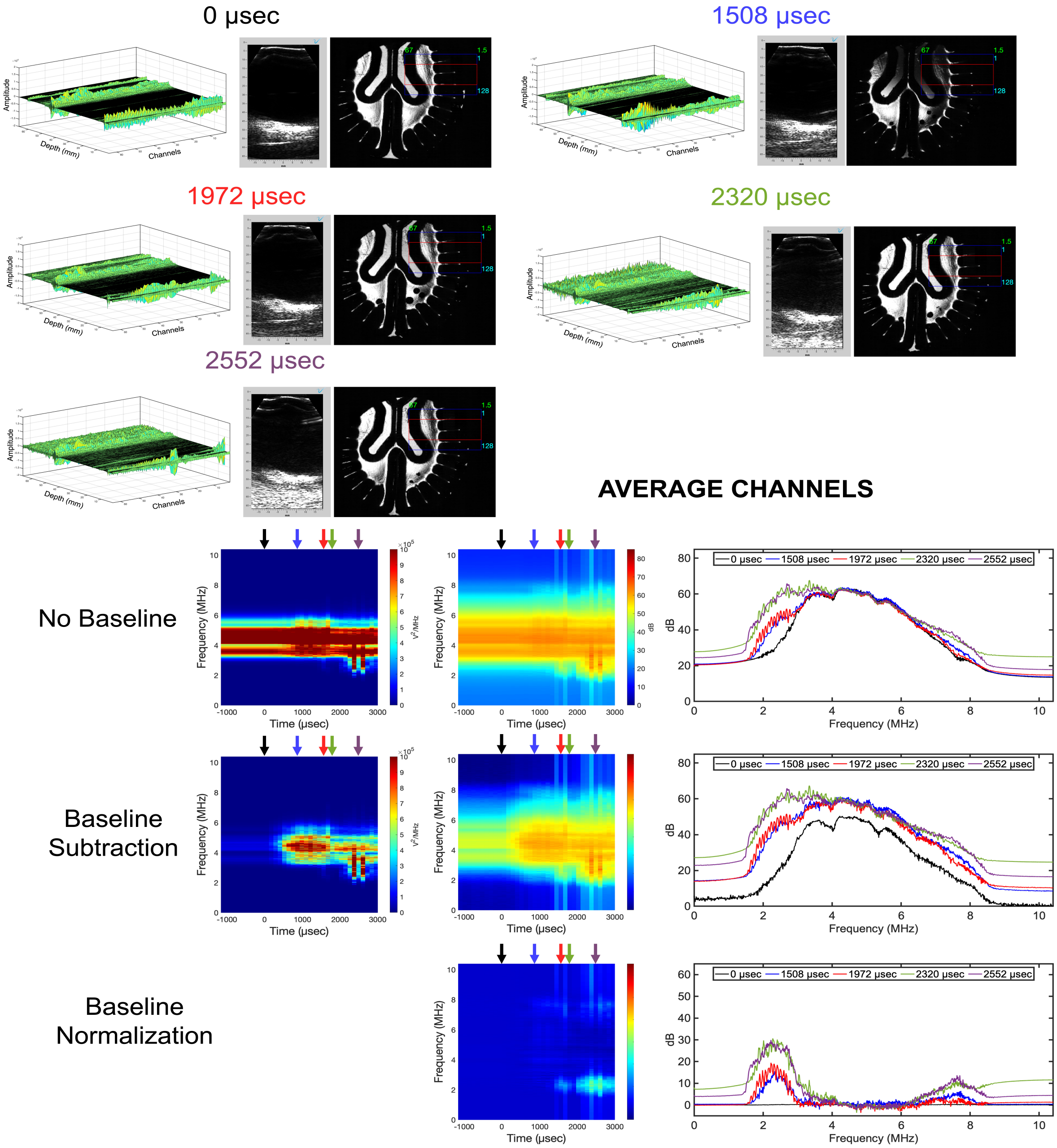

Figure 23: Comparison of averaged received channel signal processing operations applied to the acoustic power spectra from the second test of a head model filled with an ANGUS brain phantom, using plane wave imaging (PWI) as the acquisition method and observing the ventricular region. The top section presents 3D representations of RF data at different time points, along with corresponding shadowgraph frames. The acoustic transmit field of view is indicated by a blue box (channels 1–128), and the receive channels (red box) span 32–96. Channel mapping is such that receive channels 1–64 correspond to channels 32–96, respectively. The axial depth ranges from 1.5 to 67 mm, with all channels labeled accordingly. Following this, linear and logarithmic power spectrograms are presented, categorized by signal processing method: no baseline correction, baseline subtraction, and baseline normalization.

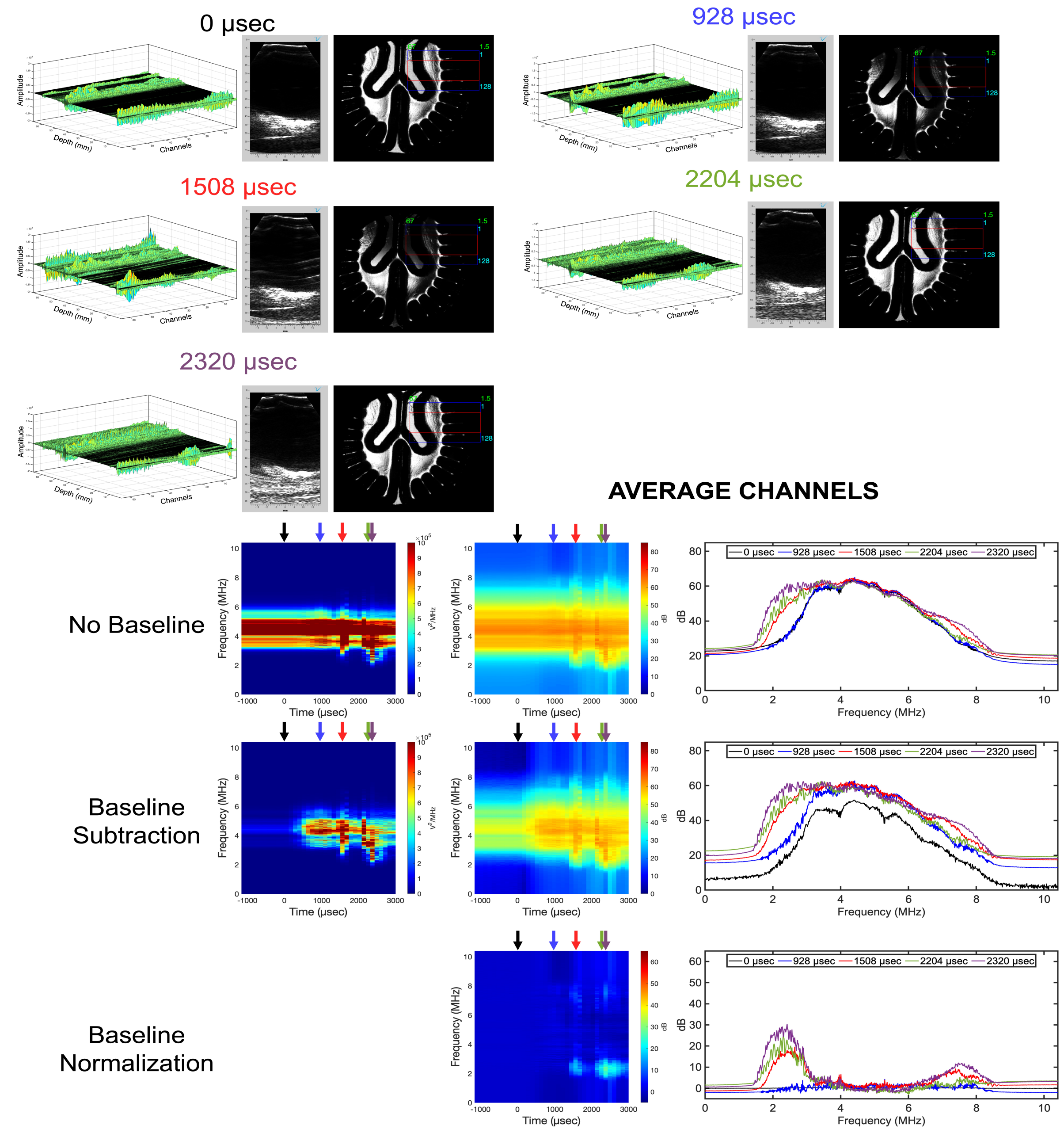

Figure 24: Comparison of averaged received channel signal processing operations applied to the acoustic power spectra from the third test of a head model filled with an ANGUS brain phantom, using plane wave imaging (PWI) as the acquisition method and observing the ventricular region. The top section presents 3D representations of RF data at different time points, along with corresponding shadowgraph frames. The acoustic transmit field of view is indicated by a blue box (channels 1–128), and the receive channels (red box) span 32–96. Channel mapping is such that receive channels 1–64 correspond to channels 32–96, respectively. The axial depth ranges from 1.5 to 67 mm, with all channels labeled accordingly. Following this, linear and logarithmic power spectrograms are presented, categorized by signal processing method: no baseline correction, baseline subtraction, and baseline normalization.

Central Sulcus

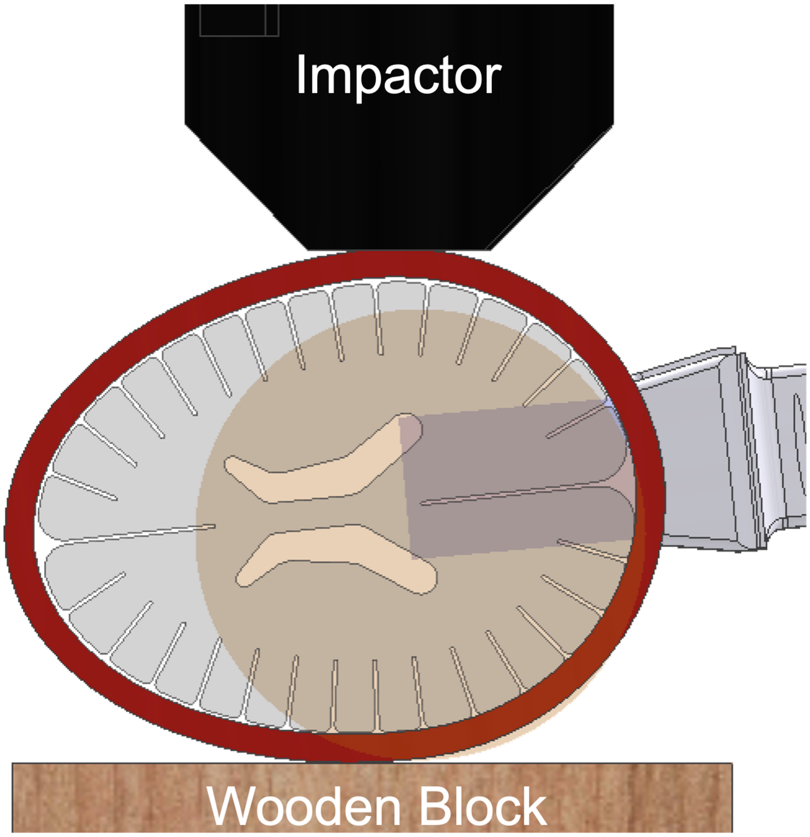

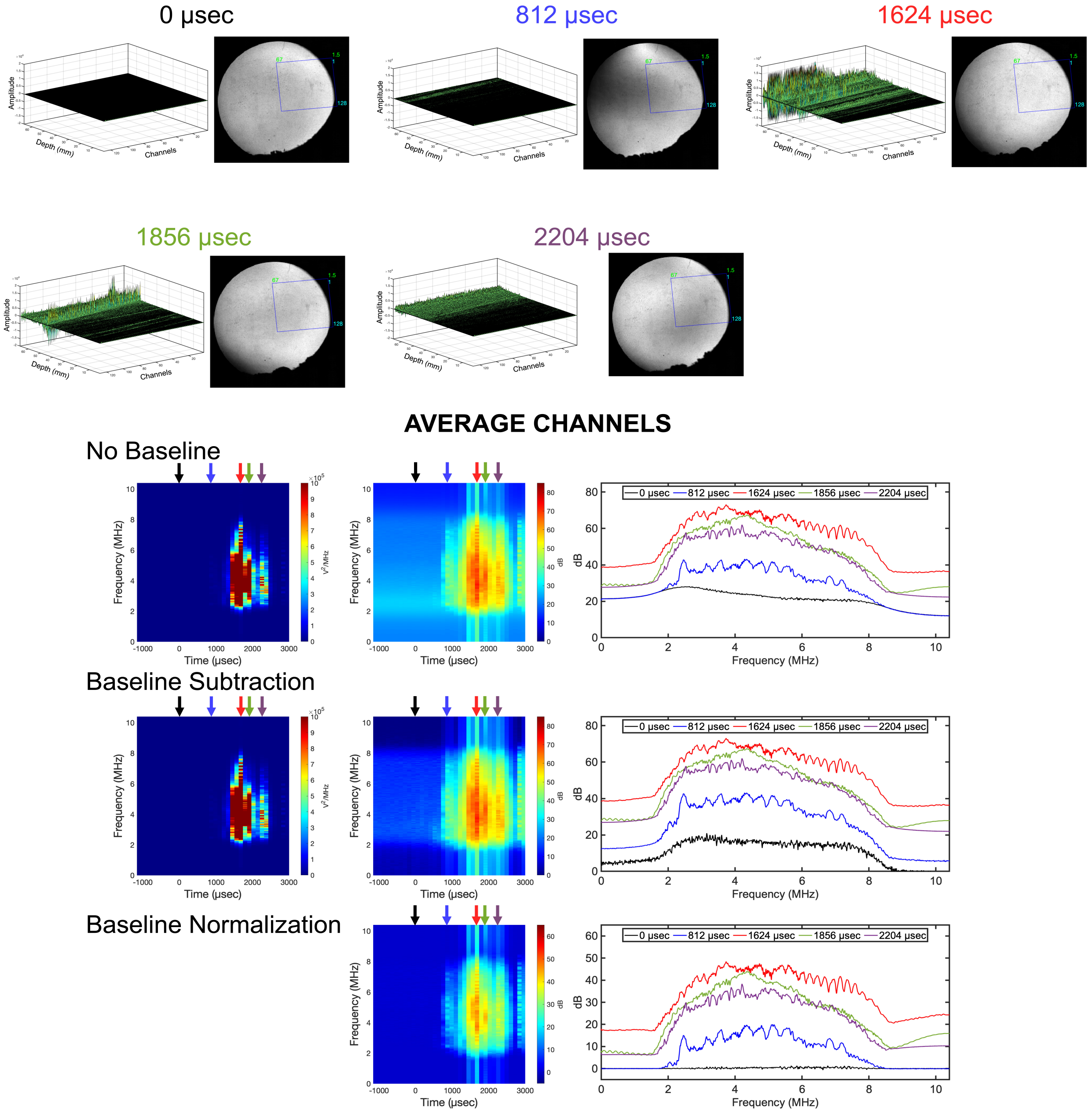

Figure 25: Comparison of averaged received channel signal processing operations on the acoustic power spectra from the first test of a head model filled with DI water, using passive cavitation detection (PCD) as the acquisition method while observing along the central sulcus. The top section presents the RF data at different time points in a 3D representation, along with the corresponding shadowgraph frames. The acoustic field of view is shown as a blue box, with channels (1–128) and axial depth (1.5–67 mm) labeled. Following this are the linear and logarithmic power spectrograms, categorized by signal processing method: no baseline correction, baseline subtraction, and baseline normalization.

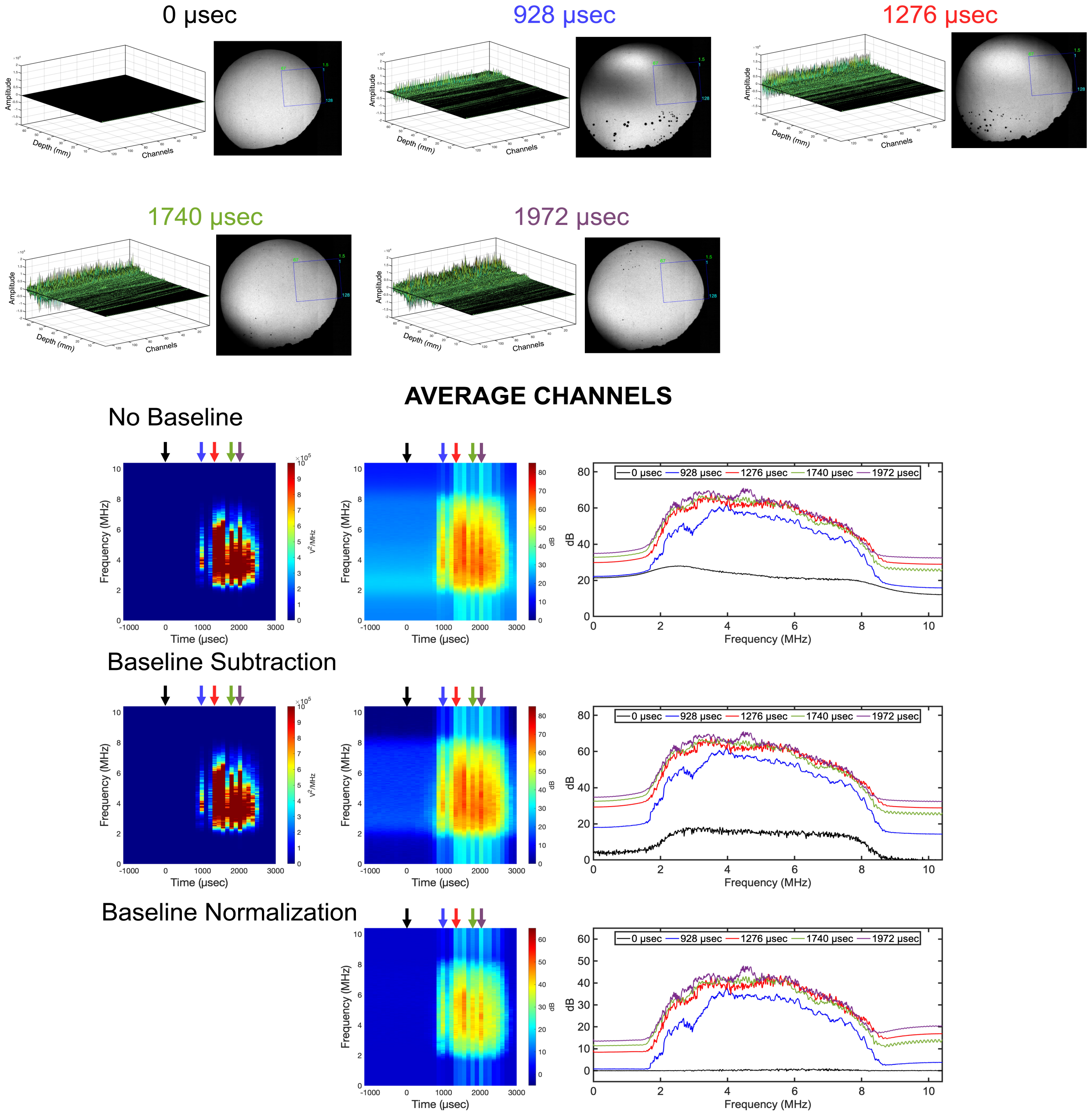

Figure 26: Comparison of averaged received channel signal processing operations on the acoustic power spectra from the second test of a head model filled with DI water, using passive cavitation detection (PCD) as the acquisition method while observing along the central sulcus. The top section presents the RF data at different time points in a 3D representation, along with the corresponding shadowgraph frames. The acoustic field of view is shown as a blue box, with channels (1–128) and axial depth (1.5–67 mm) labeled. Following this are the linear and logarithmic power spectrograms, categorized by signal processing method: no baseline correction, baseline subtraction, and baseline normalization.

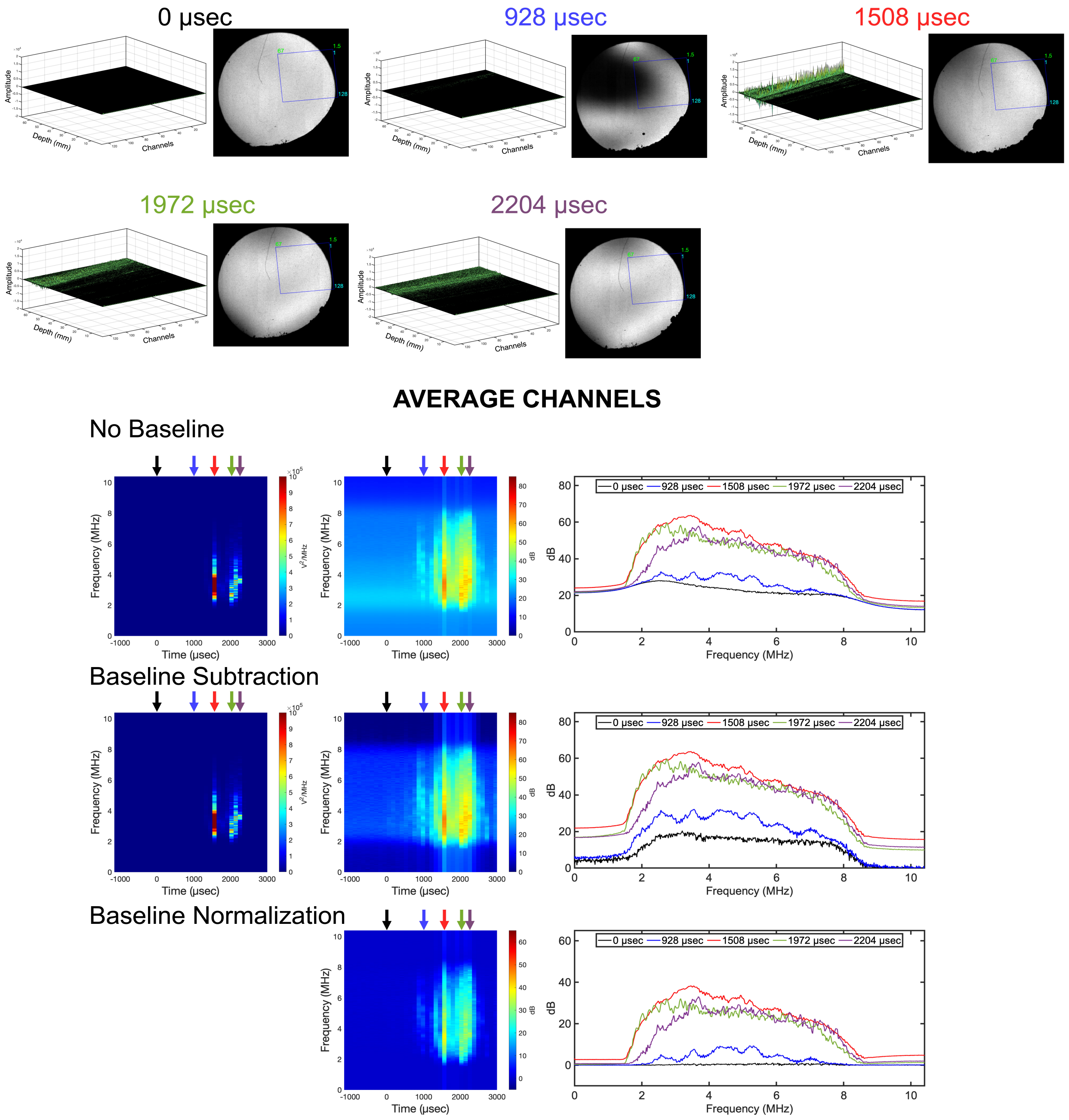

Figure 27: Comparison of averaged received channel signal processing operations on the acoustic power spectra from the third test of a head model filled with DI water, using passive cavitation detection (PCD) as the acquisition method while observing along the central sulcus. The top section presents the RF data at different time points in a 3D representation, along with the corresponding shadowgraph frames. The acoustic field of view is shown as a blue box, with channels (1–128) and axial depth (1.5–67 mm) labeled. Following this are the linear and logarithmic power spectrograms, categorized by signal processing method: no baseline correction, baseline subtraction, and baseline normalization.

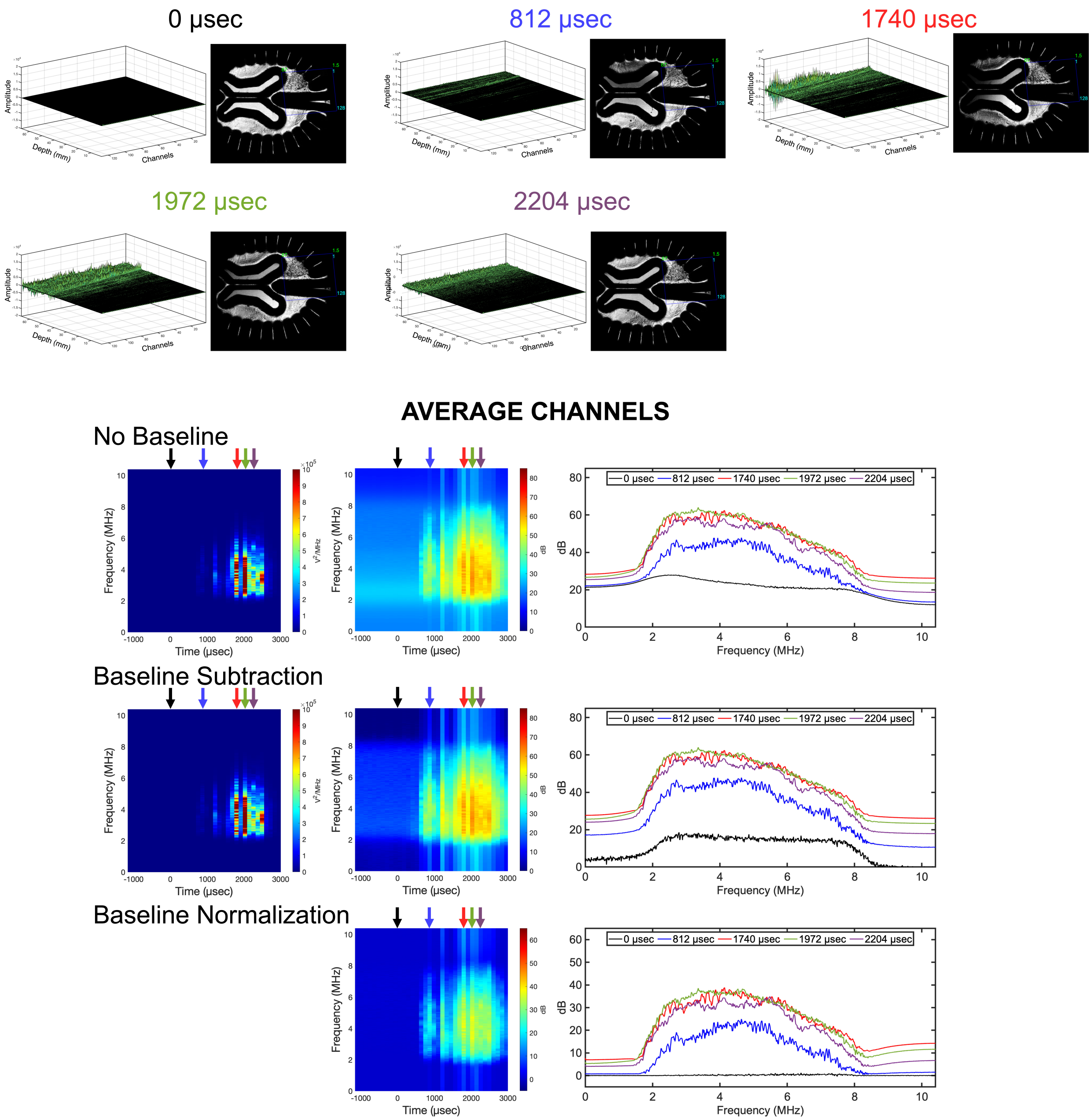

Figure 28: Comparison of averaged received channel signal processing operations on the acoustic power spectra from the first test of a head model filled with an ANGUS brain phantom, using passive cavitation detection (PCD) as the acquisition method while observing along the central sulcus. The top section presents the RF data at different time points in a 3D representation, along with the corresponding shadowgraph frames. The acoustic field of view is shown as a blue box, with channels (1–128) and axial depth (1.5–67 mm) labeled. Following this are the linear and logarithmic power spectrograms, categorized by signal processing method: no baseline correction, baseline subtraction, and baseline normalization.

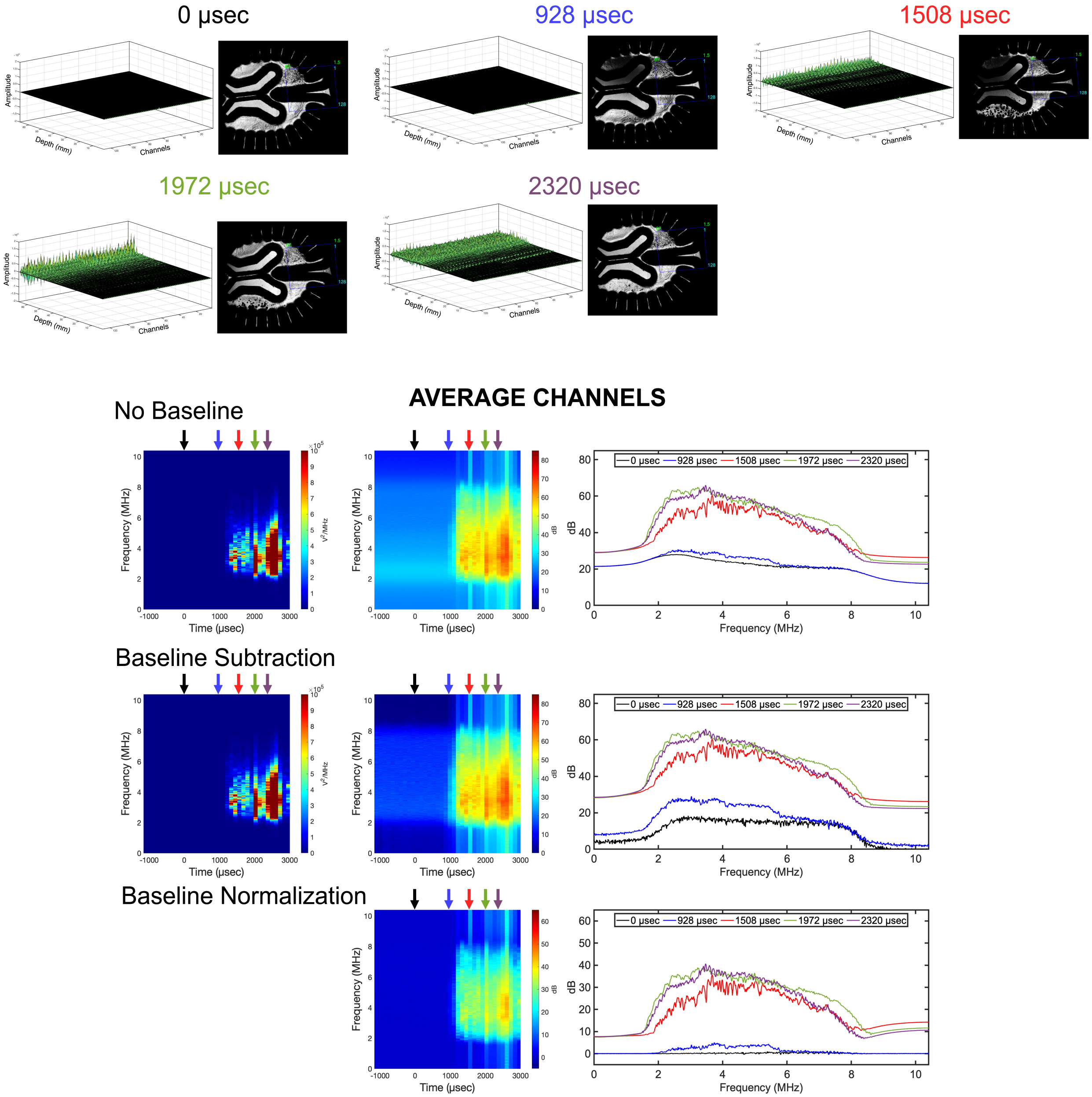

Figure 29: Comparison of averaged received channel signal processing operations on the acoustic power spectra from the second test of a head model filled with an ANGUS brain phantom, using passive cavitation detection (PCD) as the acquisition method while observing along the central sulcus. The top section presents the RF data at different time points in a 3D representation, along with the corresponding shadowgraph frames. The acoustic field of view is shown as a blue box, with channels (1–128) and axial depth (1.5–67 mm) labeled. Following this are the linear and logarithmic power spectrograms, categorized by signal processing method: no baseline correction, baseline subtraction, and baseline normalization.

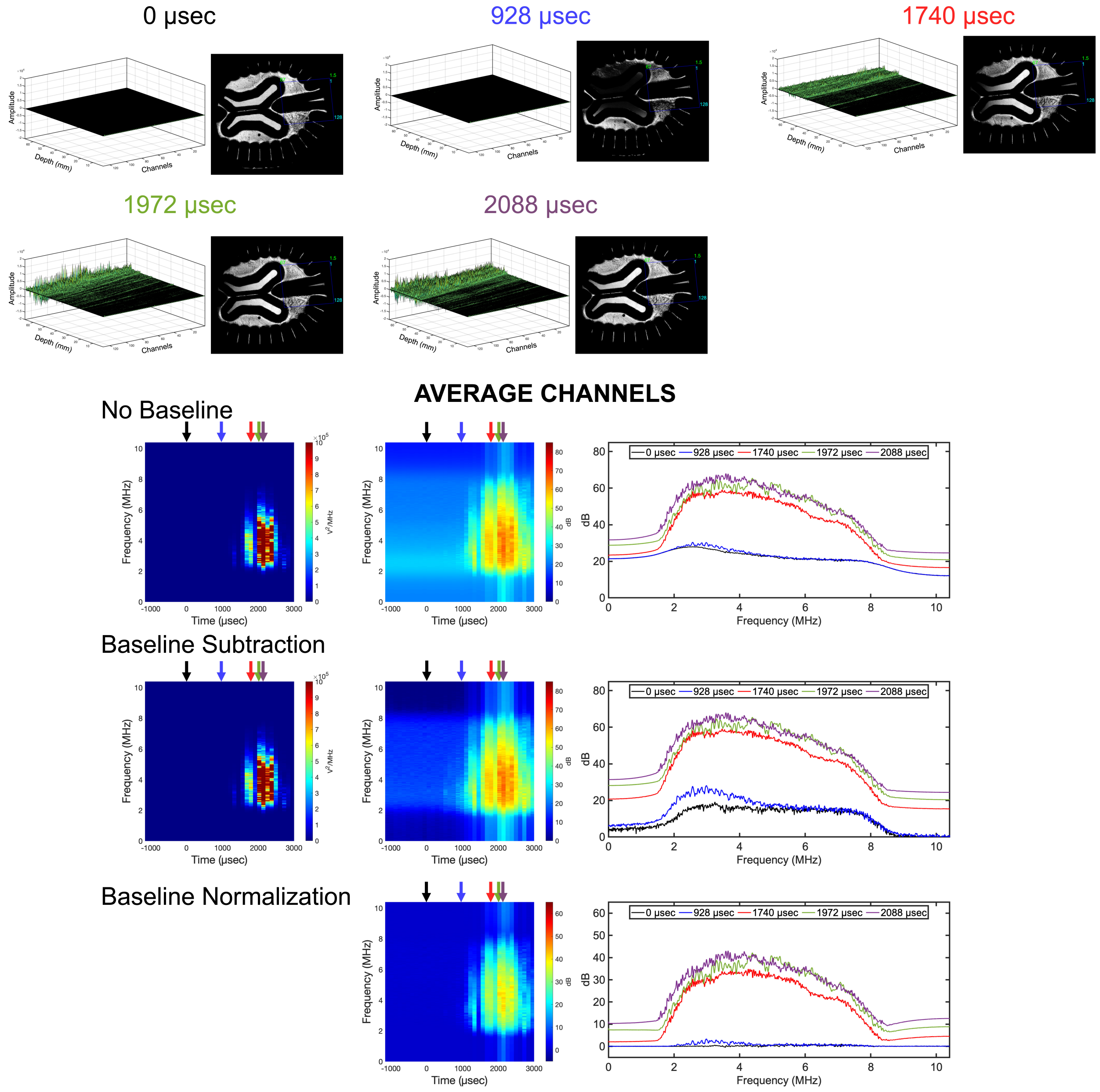

Figure 30: Comparison of averaged received channel signal processing operations on the acoustic power spectra from the third test of a head model filled with an ANGUS brain phantom, using passive cavitation detection (PCD) as the acquisition method while observing along the central sulcus. The top section presents the RF data at different time points in a 3D representation, along with the corresponding shadowgraph frames. The acoustic field of view is shown as a blue box, with channels (1–128) and axial depth (1.5–67 mm) labeled. Following this are the linear and logarithmic power spectrograms, categorized by signal processing method: no baseline correction, baseline subtraction, and baseline normalization.

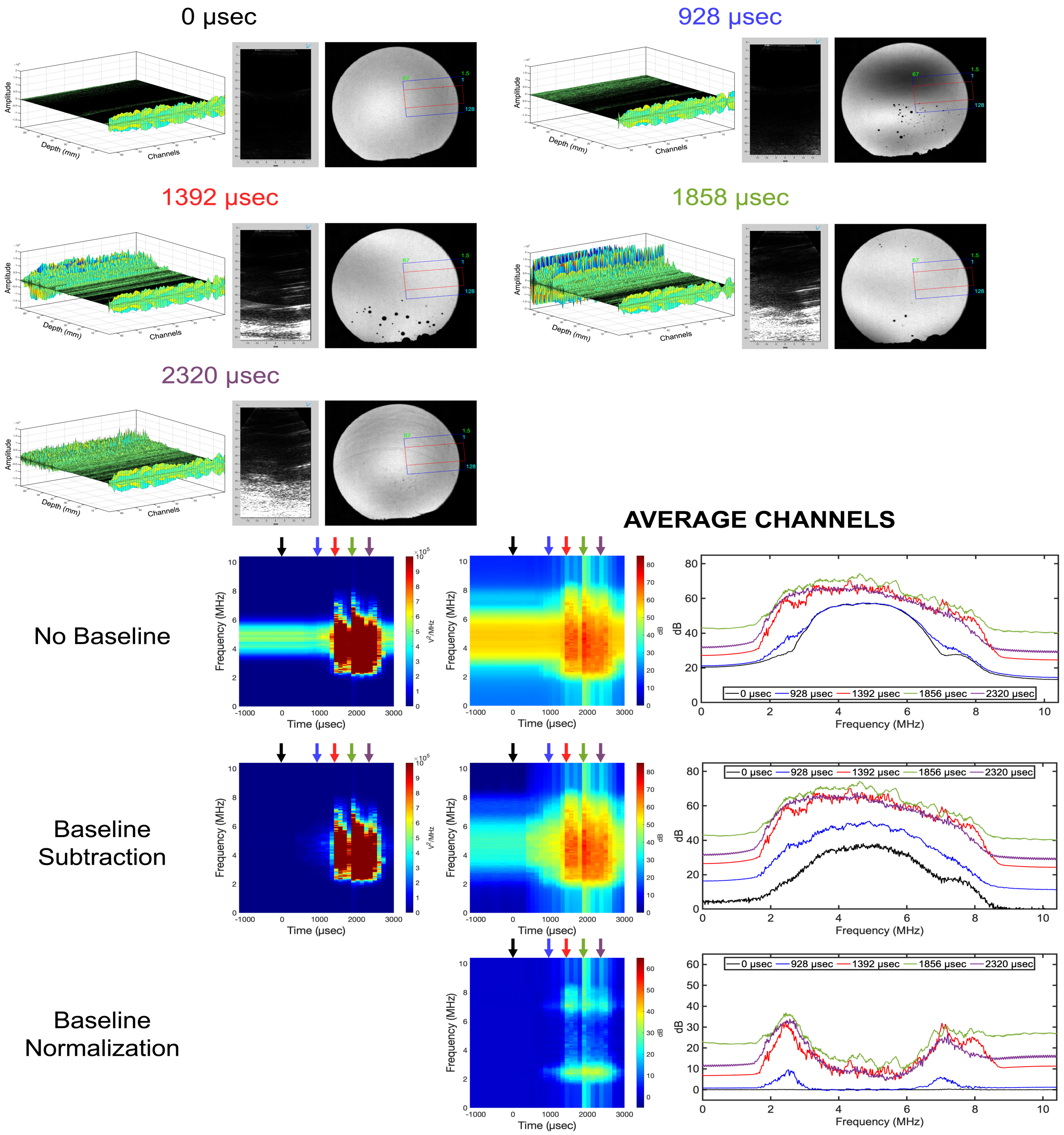

Figure 31: Comparison of averaged received channel signal processing operations applied to the acoustic power spectra from the first test of a head model filled with DI water, using plane wave imaging (PWI) as the acquisition method and observing along the central sulcus. The top section presents 3D representations of RF data at different time points, along with corresponding shadowgraph frames. The acoustic transmit field of view is indicated by a blue box (channels 1–128), and the receive channels (red box) span 32–96. Channel mapping is such that receive channels 1–64 correspond to channels 32–96, respectively. The axial depth ranges from 1.5 to 67 mm, with all channels labeled accordingly. Following this, linear and logarithmic power spectrograms are presented, categorized by signal processing method: no baseline correction, baseline subtraction, and baseline normalization.

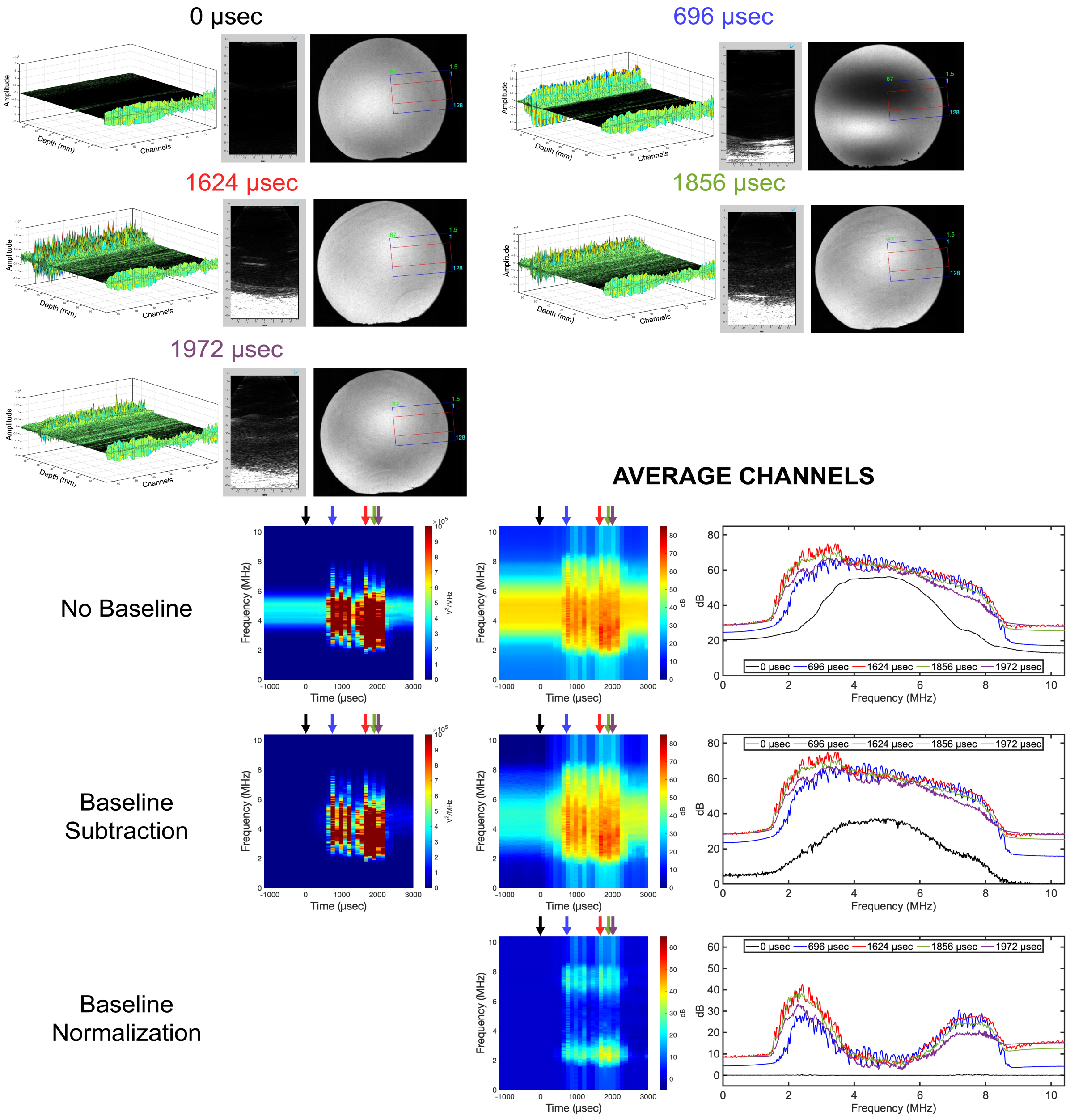

Figure 32: Comparison of averaged received channel signal processing operations applied to the acoustic power spectra from the second test of a head model filled with DI water, using plane wave imaging (PWI) as the acquisition method and observing along the central sulcus. The top section presents 3D representations of RF data at different time points, along with corresponding shadowgraph frames. The acoustic transmit field of view is indicated by a blue box (channels 1–128), and the receive channels (red box) span 32–96. Channel mapping is such that receive channels 1–64 correspond to channels 32–96, respectively. The axial depth ranges from 1.5 to 67 mm, with all channels labeled accordingly. Following this, linear and logarithmic power spectrograms are presented, categorized by signal processing method: no baseline correction, baseline subtraction, and baseline normalization.

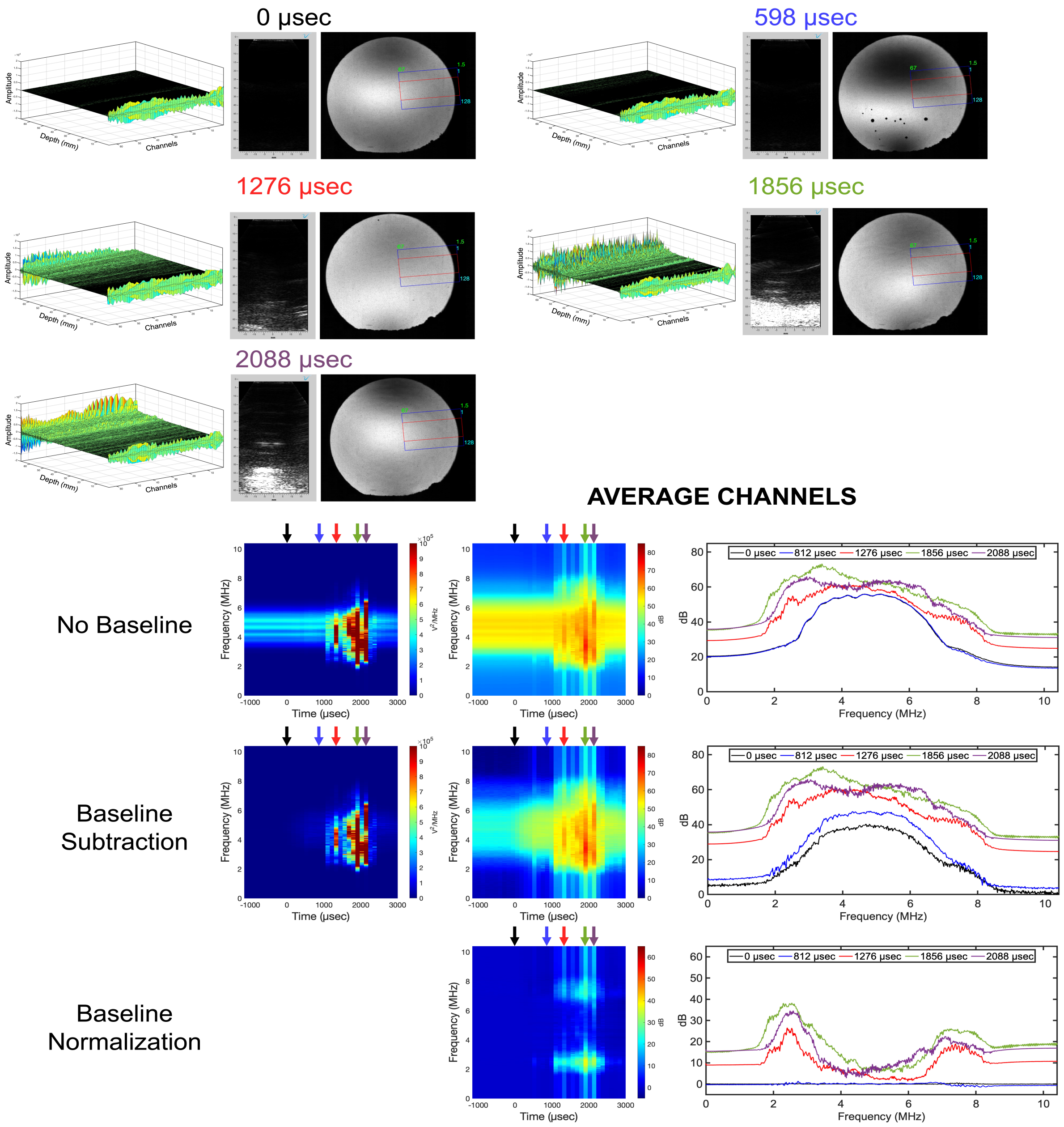

Figure 33: Comparison of averaged received channel signal processing operations applied to the acoustic power spectra from the third test of a head model filled with DI water, using plane wave imaging (PWI) as the acquisition method and observing along the central sulcus. The top section presents 3D representations of RF data at different time points, along with corresponding shadowgraph frames. The acoustic transmit field of view is indicated by a blue box (channels 1–128), and the receive channels (red box) span 32–96. Channel mapping is such that receive channels 1–64 correspond to channels 32–96, respectively. The axial depth ranges from 1.5 to 67 mm, with all channels labeled accordingly. Following this, linear and logarithmic power spectrograms are presented, categorized by signal processing method: no baseline correction, baseline subtraction, and baseline normalization.

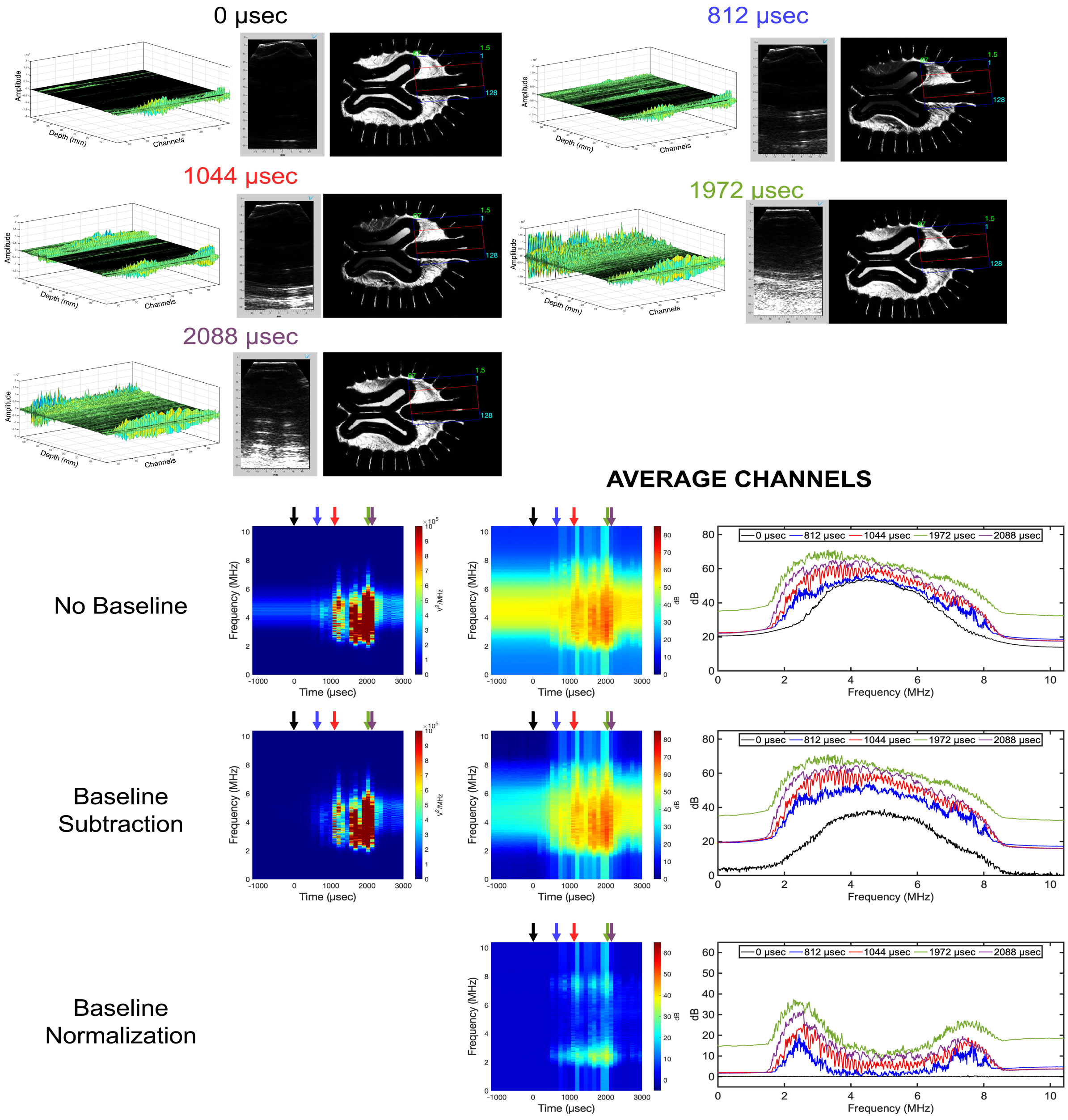

Figure 34: Comparison of averaged received channel signal processing operations applied to the acoustic power spectra from the first test of a head model filled with an ANGUS brain phantom, using plane wave imaging (PWI) as the acquisition method and observing along the central sulcus. The top section presents 3D representations of RF data at different time points, along with corresponding shadowgraph frames. The acoustic transmit field of view is indicated by a blue box (channels 1–128), and the receive channels (red box) span 32–96. Channel mapping is such that receive channels 1–64 correspond to channels 32–96, respectively. The axial depth ranges from 1.5 to 67 mm, with all channels labeled accordingly. Following this, linear and logarithmic power spectrograms are presented, categorized by signal processing method: no baseline correction, baseline subtraction, and baseline normalization.

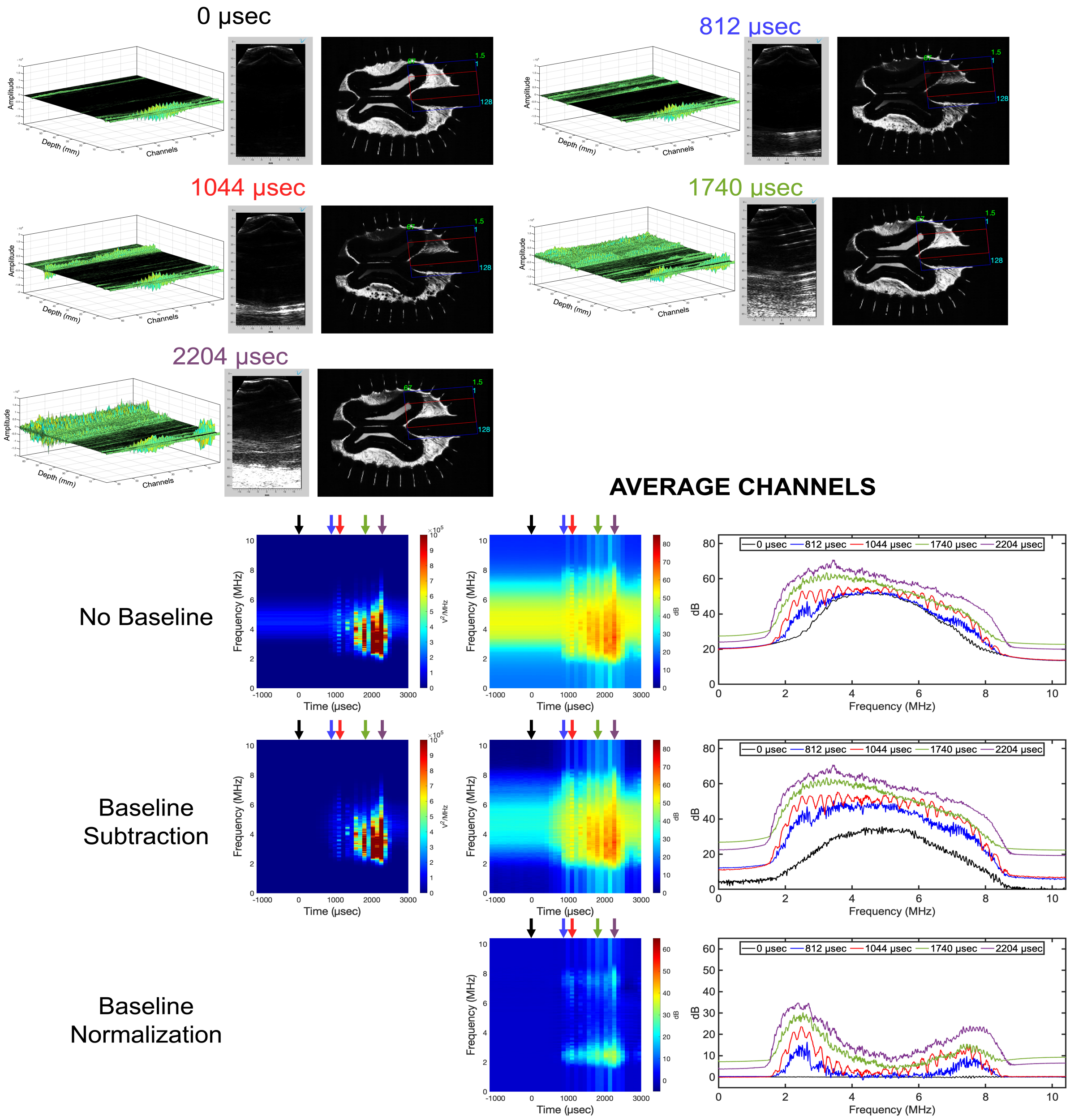

Figure 35: Comparison of averaged received channel signal processing operations applied to the acoustic power spectra from the second test of a head model filled with an ANGUS brain phantom, using plane wave imaging (PWI) as the acquisition method and observing along the central sulcus. The top section presents 3D representations of RF data at different time points, along with corresponding shadowgraph frames. The acoustic transmit field of view is indicated by a blue box (channels 1–128), and the receive channels (red box) span 32–96. Channel mapping is such that receive channels 1–64 correspond to channels 32–96, respectively. The axial depth ranges from 1.5 to 67 mm, with all channels labeled accordingly. Following this, linear and logarithmic power spectrograms are presented, categorized by signal processing method: no baseline correction, baseline subtraction, and baseline normalization.

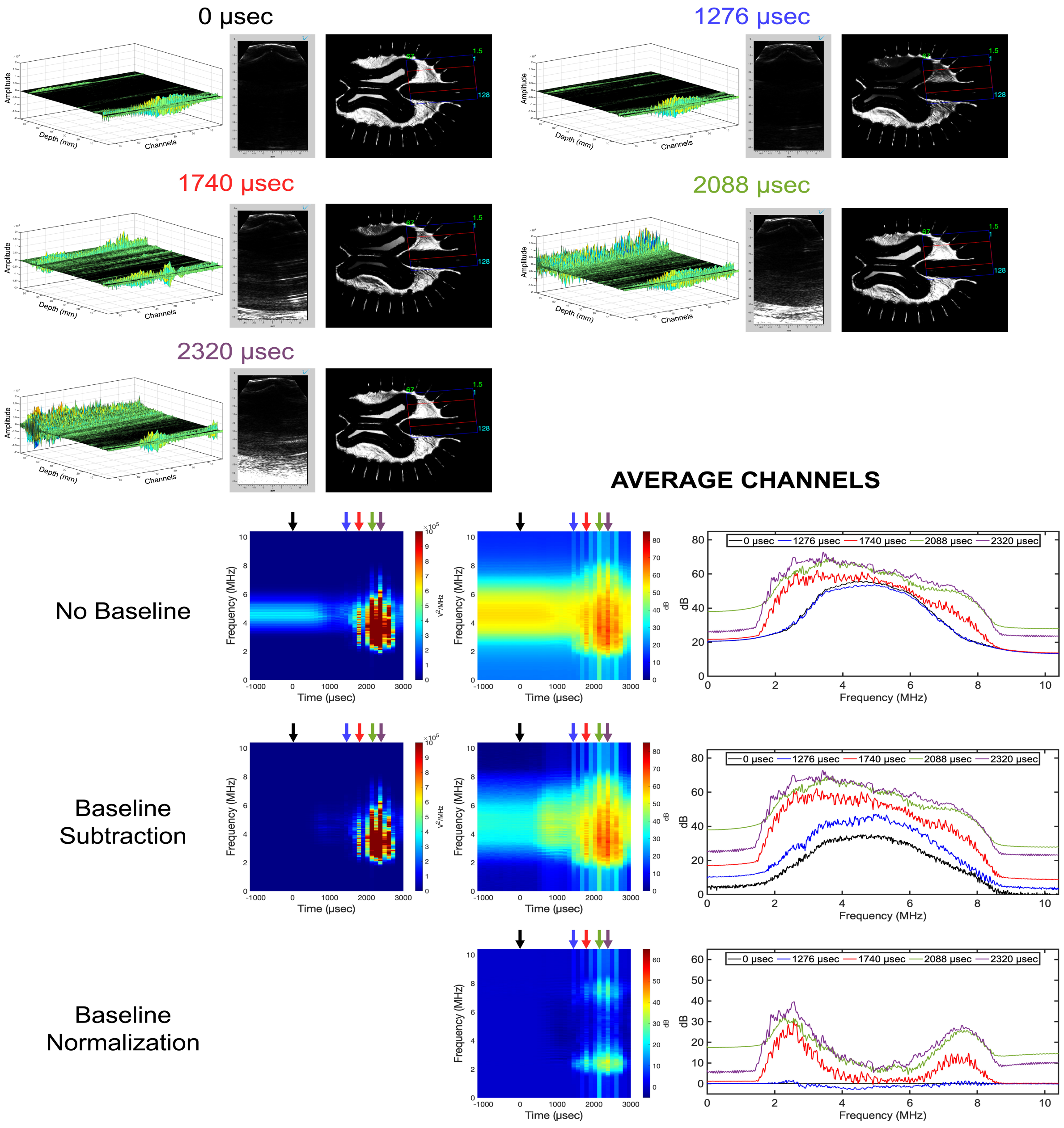

Figure 36: Comparison of averaged received channel signal processing operations applied to the acoustic power spectra from the third test of a head model filled with an ANGUS brain phantom, using plane wave imaging (PWI) as the acquisition method and observing along the central sulcus. The top section presents 3D representations of RF data at different time points, along with corresponding shadowgraph frames. The acoustic transmit field of view is indicated by a blue box (channels 1–128), and the receive channels (red box) span 32–96. Channel mapping is such that receive channels 1–64 correspond to channels 32–96, respectively. The axial depth ranges from 1.5 to 67 mm, with all channels labeled accordingly. Following this, linear and logarithmic power spectrograms are presented, categorized by signal processing method: no baseline correction, baseline subtraction, and baseline normalization.

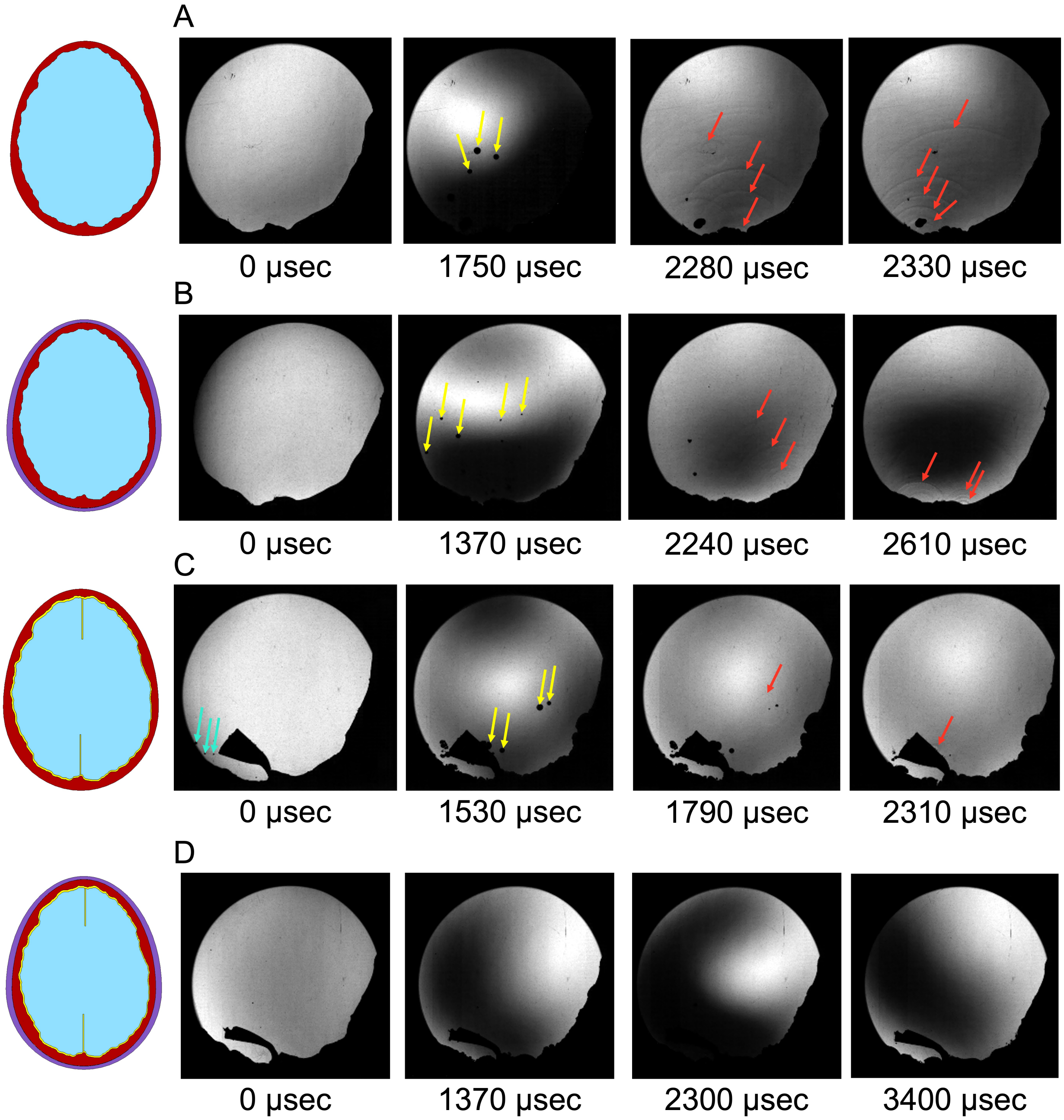

Figure 37: Shadowgraph imaging of an asymmetrical head model was conducted under blunt impact testing, using a 4 kg mass dropped from a height of 60 cm based on conditions known to induce cavitation reliably. The impacts involved: **(A)** the asymmetrical skull alone, **(B)** the skull with a scalp layer, **(C)** the skull with a dura mater layer, and **(D)** the skull with both scalp and dura mater layers. In all cases, the head models were filled with deionized (DI) water. Cyan arrows indicate pre-existing bubbles before impact, yellow arrows highlight cavitation bubble growth in the contrecoup region of the head model, and red arrows identify areas of shock wave activity, either within the DI water medium or localized within the grooves of the skull geometry. Adapted from Galindo et al. [37].

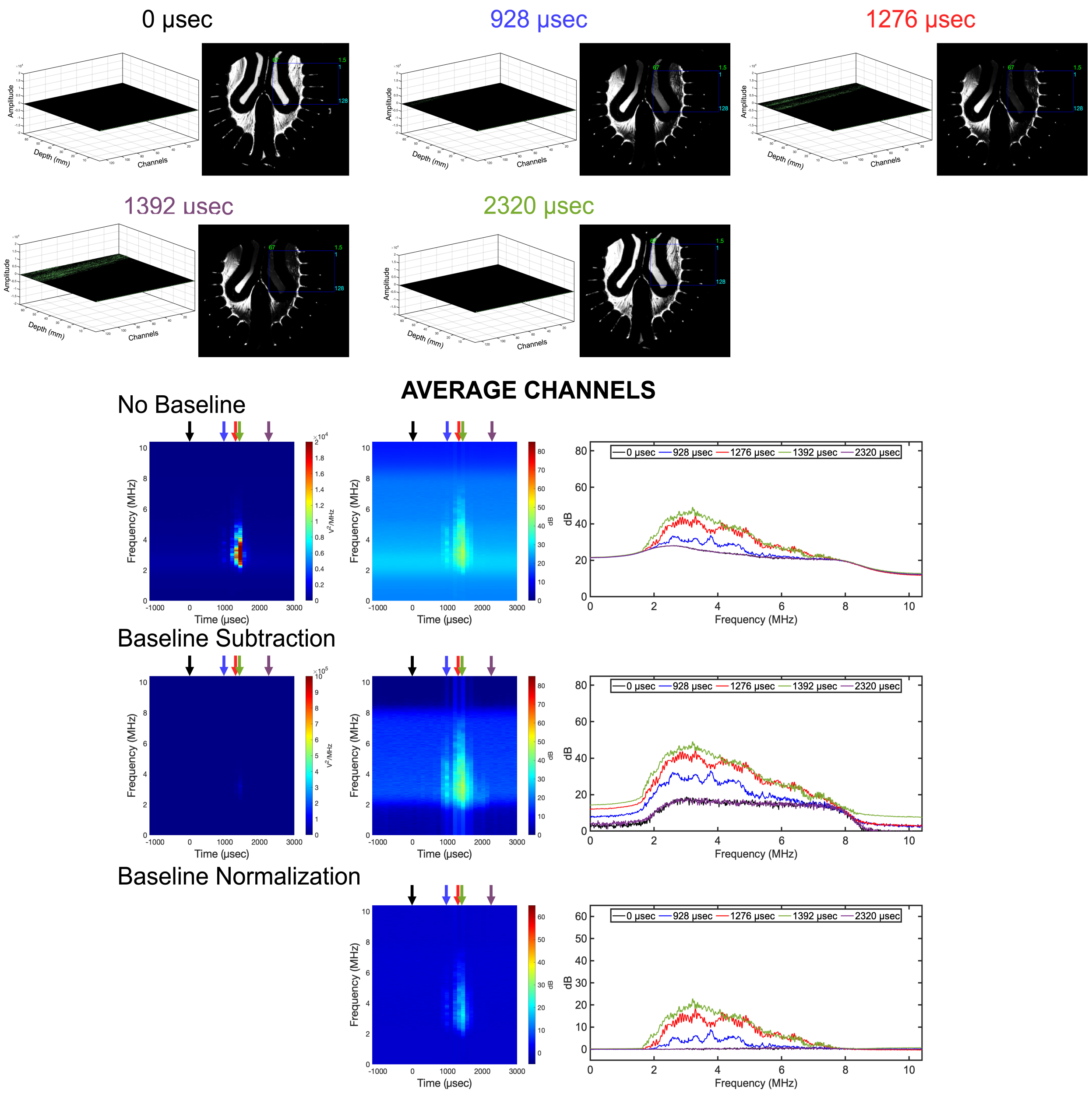

Figure 38: Comparison of averaged received channel signal processing methods on acoustic power spectra acquired from a head model featuring temporal bone skull geometry, artificial dampeners, and an ANGUS brain phantom where PCD was used to monitor acoustic emissions within the ventricular region. The top section presents the RF data at different time points in a 3D representation, along with the corresponding shadowgraph frames. The acoustic field of view is shown as a blue box, with channels (1–128) and axial depth (1.5–67 mm) labeled. Following this are the linear and logarithmic power spectrograms, categorized by signal processing method: no baseline correction, baseline subtraction, and baseline normalization.

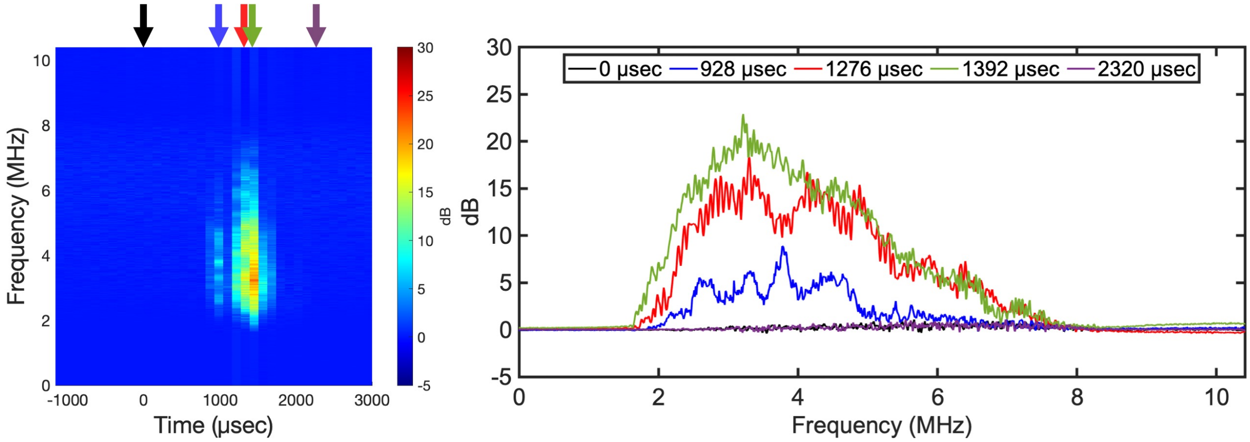

Figure 39: Rescaling of baseline-normalized spectrograms and spectra from the temporal bone configuration with integrated artificial dampeners and an ANGUS brain phantom, as shown in Figure 38.
